# Insights into the bacterial fraction of sediments from the South China Sea: physiological, chemotaxonomic, and genomic characterization of seven novel members of the *Bacillaceae* family

**DOI:** 10.3389/fmicb.2025.1553679

**Published:** 2025-07-04

**Authors:** Shu-Tong Ge, Hong Cheng, Ying-Wen Zhong, Jia-Ling Tang, Xin-Yue Huang, Bin Wei, Yue-Hong Wu, Chun-Sheng Wang

**Affiliations:** ^1^State Key Laboratory of Submarine Geoscience, Second Institute of Oceanography, Ministry of Natural Resources, Hangzhou, China; ^2^Key Laboratory of Marine Ecosystem Dynamics, Ministry of Natural Resources & Second Institute of Oceanography, Ministry of Natural Resources, Hangzhou, China; ^3^Southern Marine Science and Engineering Guangdong Laboratory (Zhuhai), Zhuhai, China; ^4^School of Oceanography, Shanghai Jiao Tong University, Shanghai, China; ^5^College of Pharmaceutical Science & Collaborative Innovation Center of Yangtze River Delta Region Green Pharmaceuticals, Zhejiang University of Technology, Hangzhou, China

**Keywords:** *Bacillaceae*, novel species, comparative genomic, CAZymes, BGCs

## Abstract

The family *Bacillaceae* is phenotypically and phylogenetically heterogeneous group of bacteria, which has vast metabolic capability in carbohydrates degradation and secondary metabolite production. Deep marine sediments harbor highly diverse microorganisms, playing important roles in ecosystem. Here, we investigated the cultivable fraction of bacteria associated with the sediments of South China Sea (*n* = 152). After obtaining candidate novel strains, the morphological and physiological characteristics analysis were conducted for polyphasic taxonomy. Additionally, the whole genome sequencing, annotation and comparative genomic analysis were performed for their specific metabolic characteristics. As a result, seven novel members of the family *Bacillaceae* have been established: *Pseudalkalibacillus nanhaiensis* sp. nov. (Strain SCS-8^T^), *Paraperibacillus marinus* sp. nov. (Strain SCS-26^T^), *Neobacillus oceani* sp. nov. (Strain SCS-31^T^), *Paraperibacillus esterisolvens* sp. nov. (Strain SCS-37^T^), *Nanhaiella sioensis* gen. nov., sp. nov. (Strain SCS-151^T^), *Rossellomorea sedimentorum* sp. nov. (Strain SCS-153A^T^) and *Peribacillus sedimenti* sp. nov. (Strain SCS-155^T^). These novel srains display smaller genome sizes and distinctive characteristics. The annotation of Cluster of Orthologous Genes (COG) revealed a higher specific gene abundance in these strains in the carbohydrate transport and metabolism (COG-G), secondary metabolites processes (COG-Q), and the cell membrane-related functions (COG-M). These *Bacillaceae* species isolated from sediment have different capability to degrade carbohydrates and produce biosynthetic products compared to other reference strains, revealing that they have unique adaptation strategies to the deep marine sediments.

## 1 Introduction

Marine sediments harbor the largest reservoir of organic and inorganic materials and contain highly diverse microbial ecosystems. Almost 2.9 × 10^29^ cells are living at and below the seafloor, which is roughly equal to previous estimates of total microbial abundance in seawater and soil (Kallmeyer et al., 2012). Sediment microorganisms contribute to supporting almost half of the global primary productivity, driving oceanic biogeochemical cycles, and metabolizing foreign compounds (Swaathy et al., 2014). Due to the difficulty of subseafloor sediments sampling, there are still many undiscovered microbes that contain abundant biological resources.

The family *Bacillaceae* (phylum *Bacillota*; class *Bacilli*; order *Caryophanales*) is a globally dispersed and phenotypically heterogeneous group of bacteria, characterized mainly by Gram-positive, aerobic heterotrophs and endospores forming (Ciccarelli et al., 2006; Harirchi et al., 2022). As a dormant life form, an endospore can increase resilience to stressors in harsh environmental conditions, including nutrient limitation, extreme heat, ultraviolet radiation (UV), chemicals, desiccation, and other stresses (Beskrovnaya et al., 2021; Setlow, 2014). Due to the low microbial activity of the endospore, some researchers reported that viable spores have been isolated from dried plant samples dating from 1,640 onward, suggesting microbial biomass turnover times of hundreds to thousands of years (Lomstein et al., 2012; Sneath, 1962). Besides, the endospore can be randomly dispersed by ocean currents and hydrothermal plumes, and distributed across marine subseafloor sediments eventually (Gittins et al., 2022; Hubert et al., 2009; Müller et al., 2014). Compared to dormant endospores, the vegetative cells have various metabolic capabilities. Based on laboratory studies of species in the pure culture, it was found that *Bacillaceae* has a wide range of carbohydrate enzymes, including those with the ability to degrade various polysaccharides such as starch, plant cellulose, and lignin (Fajardo-Cavazos et al., 2014). It has also been observed that there is a capability to release secondary metabolites to provide resistance and protect the host cell. The *Bacillaceae* from the marine environment produced diverse and different metabolites, including a substrate-degrading enzyme, such as a new glucanase (Okami et al., 1980) and antibacterial secondary metabolites (Barzkar et al., 2024) like cyclic acylpeptides (Trischman et al., 1994). From the studies of functional annotation and comparative genomics, the members of *Bacillaceae* show few core genes and a wide range of accessory genes, which highlights the diverse environments encountered by *Bacillaceae* (Alcaraz et al., 2010). The diversity of accessory genes reveals species lineage differentiation and genetic differences, thereby increasing the complexity of phylogenetic trees.

The basal position of the *Bacillota* in the bacterial tree of life suggests that *Bacillaceae* have shown remarkable phylogenetic diversity over nearly 2 billion years of evolution (Moreno-Letelier et al., 2011). In the past, many attempts were made to resolve the issue of *Bacillaceae* heterogeneity. However, with only 16S ribosomal RNA (rRNA) gene trees to reclassify, many species still face a limitation to reliably distinguish taxa at the species level (Dunlap, 2019; Logan et al., 2009). In 2020, Gupta and Patel used 1,172 core proteins and 87 conserved proteins from the genomes of 352 *Bacillaceae* species to reclassify the *Bacillaceae* family to move one step closer to resolving the polyphyly problem (Gupta et al., 2020; Patel and Gupta, 2020).

In recent years, most of our knowledge of *Bacillaceae* biology facets comes from relatively few members of the family, most notably *Bacillus subtilis* and *Bacillus cereus*. However, much less information has been obtained from other major members of the *Bacillaceae* to understand their biology and genomic facets. In this study, we focused on seven novel *Bacillaceae* strains isolated from deep marine sediment of the South China Sea. We conducted polyphasic identification, analyzed their genomic characteristics and metabolic functions, and explored their diverse phylogenetic positions. Besides, we obtained their complete genome sequences through the Illumina next-generation sequencing (NGS) and Oxford Nanopore Technology (ONT) platforms. Functional genes annotation, carbohydrate-active enzymes (CAZymes) family identification, biosynthetic gene clusters (BGCs) prediction, and comparative genomic analysis were performed to broaden the gene pools and investigate the novel strains' evolutionary relationships and metabolic adaptations.

## 2 Materials and methods

### 2.1 Study sites and sample collection

**Figure 1:**
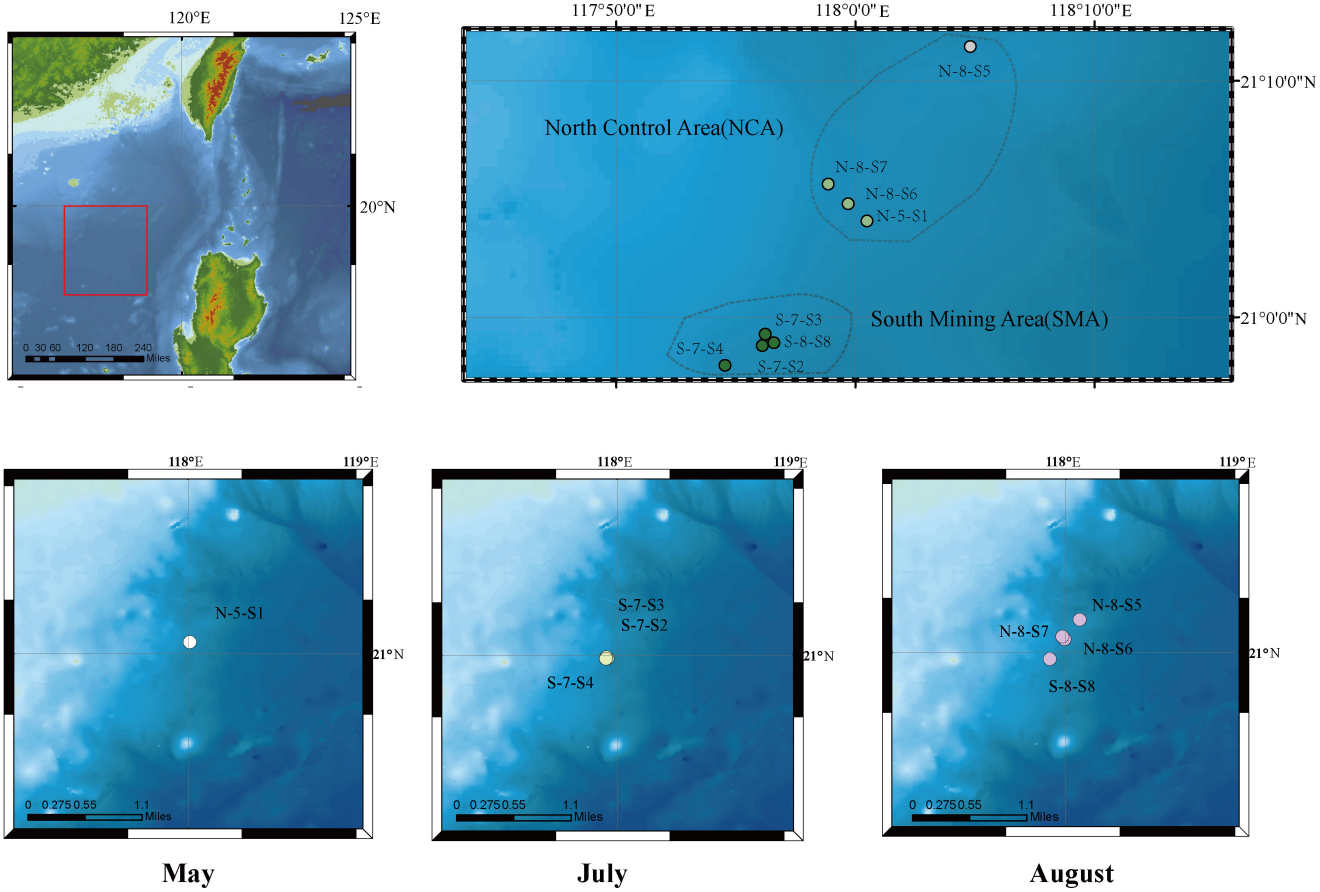
Geographic location of eight sampling sites in the South China Sea (SCS).

The marine sediment samples in this study were collected using a multi-corer from eight sites in the South China Sea in May, July, and August 2021 (Figure 1, Supplementary Table S1). Sediment layers of these cores from 0 to 10 centimeters below the seafloor (cmbsf) were obtained as 33 samples at intervals of 1 cm. Based on their locations, the sampling area was divided into two groups: the Southern Mining Area (SMA) and the Northern Control Area (NCA). The sites were labeled as N-5-S1, S-7-S2, S-7-S3, S-7-S4, N-8-S5, N-8-S6, N-8-S7, and S-8-S8. These samples were stored at −20°C until further analysis.

### 2.2 Amplicon sequencing used for bacterial diversity

Genomic DNA of sediments was prepared and sequenced at Zhejiang Tianke Hi-Tech Development Co., Ltd. (Tianke, Hangzhou, China). Genomic DNA was extracted using PowerSoil^®^ DNA Isolation Kit (MO BIO, Catalog No. 12888) for amplicon sequencing. The 16S rRNA genes targeting the V4 region (515F-806R) were amplified using specific primers with barcodes. PCR reactions were conducted using Phusion^®^ High-Fidelity Polymerase Chain Reaction (PCR) Master Mix (New England Biolabs), and the resulting libraries were sequenced on an Illumina MiSeq PE300 platform (Illumina Inc., CA, USA). The raw amplicon data were subjected to analysis using the QIIME2 software pipeline (version 2022.2) (Hall and Beiko, 2018). The DADA2 automated procession was implemented for splitting, merging, and controlling the qualities of amplicon data (Callahan et al., 2016). The unique combined sequences were aligned to the SILVA version 132, which has been trimmed to the amplified region (Quast et al., 2013).

The detailed QIIME2 commands were as follows: “qiime dada2 denoise-paired” script was used for denoising and generating the feature table; “qiime feature-table summarize”; “qiime feature-table tabulate-seqs”; and “qiime metadata tabulate” scripts were applied to generate the amplified sequence variant (ASV) feature tables; “qiime feature-classifier classify-sklearn” script was utilized to perform the taxonomic classification.

All statistical analyses have been employed via R software (version 4.2.0) (R Core Team, 2022). The Shannon diversity indices were calculated with the vegan package (version 2.6-4) (Oksanen et al., 2022), and results were visualized using the ggplot2 package (version 3.4.0) (Wickham, 2016). All tests for significance were two-tailed tests, and *p* < 0.05 was considered statistically significant.

### 2.3 Isolation of cultivable strains and DNA extraction

Before serial dilution, sediment samples (1 g) were suspended and homogenized in sterile distilled seawater (9 ml). Aliquots (0.1 ml) of appropriately diluted samples (1 × 10^−3^ to 1 × 10^−5^ dilutions) were inoculated and plated on Marine Broth 2216 plates (BD Difco) and low-nutrient seawater medium plates (containing 0.5-g peptone, 0.1-g yeast extract, and 1-L seawater). After incubation at 30°C for 3 days, the colonies were streaked onto fresh medium to obtain pure colonies. Genomic DNA of these 156 bacterial strains was extracted from the liquid culture of isolated pure bacterial strains using FastPure Bacteria DNA Isolation Mini Kit (Vazyme Biotech Co., Ltd., Nanjing, China).

### 2.4 16S rRNA gene sequencing and identification

Amplification of the almost-complete 16S rRNA gene was conducted via PCR using the universal primers 27F (5′-AGAGTTTGATCMTGGCTCAG-3′) and 1492R (5′-TACGGYTACCTTGTTACGACT-3′). PCR reaction was performed in a 25 μl volume, containing 12.5-μl Green Taq™ Mix (Vazyme Biotech Co., Ltd, Nanjing, China), 0.5 μl of forward and reverse primers for each, 0.5-μl template DNA, and 11-μl H_2_O. The amplification program was conducted on PCR machine (ETC811, Eastwin Scientific Equipment, China): denaturation at 95°C for 60 s, followed by 30 cycles of denaturation at 98°C for 60 s, anneal at 55°C for 15 s and extension at 72°C for 90 s, and finally extended at 72°C for 5 min. PCR products were sequenced by an automated DNA sequencer ABI 3730 (Applied Biosystems, USA) in Sangon Biotech Co., Ltd. (Shanghai, China). The species of the isolated strains were identified via the 16S rRNA gene using the EzBioCloud server (https://www.ezbiocloud.net/) (Yoon et al., 2017). The 16S rRNA sequences of closely related strains were retrieved from GenBank, and their similarity to the present isolate was assessed at the nucleotide level.

All the 16S rDNA sequences of all the isolated strains from the sediment samples were aligned with the ClustalW (version 2.1) algorithm of MEGA X software (Kumar et al., 2018). Based on the homology of 16S rDNA sequences, a phylogenetic tree was constructed for culturable bacterial community structure, using MEGA X software to determine the taxonomy of isolates using the maximum-likelihood (ML) algorithms, with the bootstrap values set to 2,000 replicates, which was finally plotted in Interactive Tree of Life (iTOL; https://itol.embl.de/).

### 2.5 Polyphasic characterization

#### 2.5.1 Phylogenetic analysis

The obtained 16S rRNA gene sequences of seven novel strains were compared with available sequences of valid species in the EzBioCloud server (Yoon et al., 2017). The phylogenetic trees were constructed with two methods: Neighbor Joining (NJ) and Maximum Likelihood (ML), with the access of the robustness of 2,000 replicates in the MEGA X software package. Besides, the phylogenomic trees were constructed using the Up-to-date Bacterial Core Gene (UBCG) tool (Na et al., 2018), with the concatenated nucleotide sequences of 92 housekeeping core genes. To assess the taxonomic relationships of seven strains among the *Bacillaceae* family, the phylogenomic tree of 384 genomic assemblies (scaffold and complete genome sequences) downloaded from National Center for Biotechnology Information (NCBI) Genome Database was constructed based on the concatenation of 120 ubiquitous single-copy proteins using GTDB-Tk 2.3.2 software with the default parameters (Chaumeil et al., 2020) (Supplementary Table S2). The visualization was employed with the iTOL version 6 (https://itol.embl.de/).

#### 2.5.2 Morphological, physiological, and chemotaxonomic characterization

The novel species were characterized by diverse methods. Traditionally, morphological and physiological characteristics were used for classification and identification. The temperature range for growth was determined in Marine Broth 2216 (MB) by incubating cultures at 4-50°C (4, 15, 20, 25, 30, 33, 37, 42, 45, and 50°C). Growth at different pH levels (range: pH 5–11, at intervals of 0.5 pH units) were investigated with 2-(N-morpholino)ethanesulfonic (MES) acid (pH 5–6.5), piperazine-1,4-bis(ethanesulfonic) (PIPES) acid (pH 6.5–7), N-(tris(hydroxymethyl)methyl)glycine (Tricine; pH 7–9), N-cyclohexyl-2-aminoethanesulfonic (CHES) acid (pH 9–10), or N-cyclohexyl-3-aminopropanesulfonic (CAPS) acid (pH 10–11) buffers, while two different buffers were verified at the repeat node (Cheng et al., 2015). Their growth under different salt concentrations was tested, adding to the saltless-MB (prepared without NaCl) with varying amounts of NaCl (0%, 0.5%, 1.0%, 1.5%, 2.0%, 2.5%, 3.0%, 5.0%, 8.0%, 10.0%, 12.0%, 15.0%, and 18.0%). After 48 h, the OD600 was measured using the UV-1600PC Spectrophotometer (VWR). Cell motility was examined by stab culture in semi-solid MB medium containing 0.5% agar. Inoculation was performed by puncturing the medium with a straight needle and incubating at 30°C for 3 days. Motile bacteria dispersed and visibly obscured the medium.

Oxidase activity was evaluated by the oxidation of 1% (w/v) N, N, N′, N′-Tetramethyl-1,4-phenylenediamine, and catalase activity was determined by 3% hydrogen peroxide solution. Also, amylase, protease, and lipase activities were evaluated by inoculation on MB agar plates containing 0.2% starch, 0.5% casein, and 0.3% Tween 20, Tween 40, and Tween 80 as substrates, respectively. Other biochemical characteristics were tested using the API ZYM, API 20NE, API 50CHB test strips (bioMérieux), and Biolog GenIII MicroPlate (Biolog, USA) following the manufacturer's instructions. The antibiotic susceptibility test was performed using the disc diffusion method with the following antibiotics: carbenicillin (100 μg), kanamycin (30 μg), lincomycin (2 μg), penicillin (10 U), rifampicin (5 μg), erythromycin (15 μg), gentamicin (10 μg), neomycin (30 μg), and tetracycline (30 μg). After incubating at 30°C on Marine Agar plates for 2 days, bacterial cells were examined by staining with 1% uranyl acetate. The morphology of these strains was observed using a transmission electron microscope (TEM, Hitachi H7650). Quinones were extracted and purified as described by Collins and analyzed by high-performance liquid chromatography (HPLC) (Collins et al., 1977).

#### 2.5.3 Genomic characterization

Overall Genomic Relatedness Index (OGRIs) are commonly used in delineating prokaryotic species. Average nucleotide identity (ANI) and digital DNA–DNA hybridization (dDDH) are two widely used nucleotide-based OGRIs used to validate the species identity (Riesco and Trujillo, 2024). In this study, the ANI values were performed using pyani version 0.2 (Pritchard et al., 2015) with the ANIb algorithm. Digital DNA–DNA hybridization was estimated by the web service of Genome–Genome Distance Calculator (GGDC) version 3.0 (https://ggdc.dsmz.de/ggdc.php#) with Basic Local Alignment Search Tool (BLAST+) and formula 2 (Chun et al., 2018). There are 21 complete or draft genome sequences available in the NCBI Genome Database, which are closely related to seven novel strains, have been downloaded for comparative genomics analysis (Table 1). In this study, both ANI and dDDH values between the strains and the closely related species are below the thresholds for novel species designation: 95–96% and 70%, respectively (Meier-Kolthoff et al., 2013; Richter and Rosselló-Móra, 2009). The protein-based OGRIs, such as average amino acid identity (AAI), are usually coherent with phylogenomic reconstructions and could be used to complete the phylogenetic description of genera. For the analysis of genera within a family, AAI thresholds for genus delineation range from 65 to 72% (Konstantinidis and Tiedje, 2007), while for species delineation, the AAI thresholds are typically 95%−96% (Konstantinidis and Tiedje, 2005). We calculated AAI values using EzAAI (Kim et al., 2021).

**Table 1:** Genomic information of novel *Bacillaceae* species and reference strains.

**Organism**	**Strain**	**GenBank**	**Level**	**Completeness (%)**	**Size (Mb)**	**GC %**	**CDS**	**rRNA**	**tRNA**	**Isolation**	**References**
*Pseudalkalibacillus nanhaiensis*	SCS-8^T^	GCF_040126055.1	Complete	99.35	3.6223697	43	3,669	8	73	Deep-sea sediment (4–6 cmbsf) of the South China Sea	
*Pseudalkalibacillus berkeleyi*	KCTC 12718^T^	GCF 021608225.1	Draft	93.45	3.4962	39.14	3,615	5	61	Strongylocentrotus intermedius (sea urchin)	Nedashkovskaya et al., 2012
*Pseudalkalibacillus caeni*	HB172195^T^	GCF 005747095.1	Draft	99.57	4.703601	40.92	4,701	12	108	Bamen Bay mangrove forest	Mo et al., 2020
*Pseudalkalibacillus decolorationis*	DSM 14890^T^	GCF 024609785.1	Draft (scaffold)	99.03	5.272454	39.54	4,961	10	72	Wall paintings	Heyrman et al., 2003
*Pseudalkalibacillus hwajinpoensis*	MABIK MI00000821^T^	GCF 030035425.1	Complete	99.35	4.369347	40.11	4,348	27	89	Sea water	Yoon et al., 2004
*Paraperibacillus marinus*	SCS-26^T^	GCF_046237605.1	Complete	99.32	4.408267	45.72	4,334	12	85	Deep-sea sediment (6–7 cmbsf) of the South China Sea	
*Paraperibacillus esterisolven*	SCS-37^T^	GCF_046237625.1	Complete	99.32	4.270749	45.39	4,176	12	84	Deep-sea sediment (2–3 cmbsf) of the South China Sea	
*Paraperibacillus deserti*	DSM_105482^T^	GCF_016909175.1	Draft	95.15	4.363599	40	4,210	9	82	Xinjiang Province in north-west China	Zhang et al., 2011
*Paraperibacillus kribbensis*	DSM 17871^T^	GCF 000430765.1	Draft	99.35	5.052265	43	4,890	8	72	Potato cultivation in Jeju, Korea.	Lim et al., 2007
*Peribacillus sedimenti*	SCS-155^T^	GCF_046237635.1	Complete	98.91	5.112234	41.6	4,818	20	162	Deep-sea sediment (1–2 cmbsf) of the South China Sea	
*Peribacillus asahii*	MA001^T^	GCF_003570725.1	Draft	98.28	4.341281	37.55	4,171	4	26	Soil sample obtained from Shizuoka prefecture, Japan	Yumoto et al., 2004
*Peribacillus cavernae*	L5^T^	GCF_003989135.1	Draft (scaffold)	98.6	4.547591	41.4	4,440	4	58	Soil of Tenglong cave	Feng et al., 2016
*Peribacillus glennii*	V44_8^T^	GCF_003429105.1	Draft	99.03	4.469041	42.26	4,351	6	49	Viking spacecraft	Seuylemezian et al., 2020
*Neobacillus oceani*	SCS-31^T^	GCF_046237595.1	Complete	98.06	4.610304	44.9	4,566	9	88	Deep-sea sediment (7–8 cmbsf) of the South China Sea	
*Neobacillus novalis*	FJAT-14227^T^	GCF_001636395.1	Draft (scaffold)	99.35	5.668192	40.01	5,421	37	119	Soil of several disused hay fields	Heyrman et al., 2004
*Neobacillus soli*	DSM 15604^T^	GCF_002335815.1	Draft (scaffold)	99.35	5.579901	39.71	5,349	44	93	Soil of several disused hay fields	Heyrman et al., 2004
*Neobacillus piezotolerans*	YLB-04^T^	GCF_003362805.1	Draft	97.74	4.528037	45.41	4,457	1	43	Deep-sea sediments	Yu et al., 2019
*Neobacillus notoginsengisoli*	JCM 30743^T^	GCF_003515685.1	Draft	97.29	4.87403	43.78	4,704	13	74	Rhizospheric soil	Zhang et al., 2017
*Nanhaiella sioensis*	SCS-151^T^	JBKAEG000000000	Draft (scaffold)	98.24	5.314563	34.48	4,974	9	84	Deep-sea sediment (1–2 cmbsf) of the South China Sea	
*Bacillus massiliigabonensis*	Marseille-P2639^T^	GCF_900199725.1	Draft (scaffold)	98.82	5.231817	37.92	5,084	47	131	Stool sample	Liu B.-B. et al., 2023
*Bacillus timonensis*	MGYG-HGUT-01405^T^	GCF_902374785.1	Draft (scaffold)	95.88	4.646132	37.27	4,583	6	80	Human gut	Kokcha et al., 2012
*Cytobacillus citreus*	FJAT_49705^T^	GCF_018343665.1	Draft (scaffold)	93.56	5.328100	36.97	5,078	20	97	Citrus rhizosphere soil	Liu G.-H. et al., 2023
*Cytobacillus depressus*	BZ1^T^	GCF_008923245.1	Draft	98.06	5.379075	38.19	5,179	11	121	Soil sample collected from a sunflower field	Wei et al., 2016
*Rossellomorea sedimentorum*	SCS-153A^T^	GCF_046237645.1	Complete	98.24	4.200439	42.41	4,016	10	85	Deep-sea sediment (6–8 cmbsf) of the South China Sea	
*Rossellomorea salacetis*	SKP7-4^T^	GCF_003581585.1	Draft	97.65	4.675292	43.22	4,665	3	53	Shrimp paste	Daroonpunt et al., 2019
*Rossellomorea aquimaris*	TF12^T^	GCF_001648555.1	Draft	99.35	4.035445	37.31	4,046	9	83	Roots of Arthrocnemum macrostachyum	Yoon et al., 2003
*Rossellomorea marisflavi*	JCM 11544^T^	GCF_001274775.1	Draft (scaffold)	99.35	4.312088	48.57	4,353	3	52	Tidal flat of the Yellow Sea	Yoon et al., 2003
*Rossellomorea vietnamensis*	151_6^T^	GCF_009858215.1	Complete	99.35	4.597807	43.65	4,846	33	110	Vietnamese fish sauce	Noguchi et al., 2004

### 2.6 Genome *de novo* sequencing and assembly

Genomic DNA of these seven isolates were extracted using FastPure Bacteria DNA Isolation Mini Kit-box (Vazyme Biotech, Nanjing, China). Genome sequencing was carried out by the Illumina Novaseq 6000 (Illumina, Inc., CA, USA)and Nanopore PromethION (Oxford Nanopore Technology PLC., Oxford, UK) platforms at Beijing Novogene Bioinformatics Technology Co., Ltd. Illumina sequencing (PE 150) was performed using the NEBNext^®^ Ultra™ DNA Library Prep Kit (NEB, USA). Long-read Nanopore sequencing was performed using the Rapid Sequencing (SQK-LSK109) protocol. Genome assembly in this study was performed by Unicycler version 0.4.9 (Wick et al., 2017) with the default parameters, and visualized using Bandage software version 0.9.0 (Wick et al., 2015). The basic statistics of the genome were extracted using QUAST version 5.0.2 (Gurevich et al., 2013).

### 2.7 Comparative genomic analysis

Several genomes were acquired for comparative genomics analysis. There are 21 genome sequences of reference strains in the six clades that were uploaded to the Integrated Prokaryotes Genome and pangenome Analysis (IPGA) online server v1.09 (https://nmdc.cn/ipga/) for comparative genomic analysis (Liu et al., 2022).

### 2.8 Genome annotation

The tRNAs and rRNAs of genomes were predicted using tRNAscan-SE version 2.0 (Chan et al., 2021) and the RNAmmer version 1.2 (Lagesen et al., 2007), respectively. The prophages, genomic islands, and repeat sequences were distinguished via PhiSpy version 4.2.12 (Akhter et al., 2012) and RepeatMasker (Tarailo-Graovac and Chen, 2009), respectively. The coding DNA sequence (CDS) was predicted and annotated with the RAST tool kit (RASTtk), available on the Rapid Annotation using Subsystem Technology (RAST) server (https://rast.nmpdr.org/) (Aziz et al., 2008). Gene mapping was visualized using CGView software by combining the predicted results of the complete genome sequences. The chromosome and plasmid sequences of seven strains have been deposited in GenBank.

Further functional annotation and analysis of 28 strains were based on the results of Prokka version 1.11 (Seemann, 2014), and the protein sequences of CDS were blasted against via DIAMOND (Buchfink et al., 2015) program. Kyoto Encyclopedia of Genes and Genomes (KEGG) annotation was carried out on the KEGG Automatic Annotation Server (KAAS) (Moriya et al., 2007). Gene Orthology (GO) and Cluster of Orthologous Genes (COG) information were obtained by running eggNOG-mapper version 2.0.1 against the eggNOG database version 2.0 (Huerta-Cepas et al., 2019).

#### 2.8.1 Identification and classification of CAZymes families

To analyze the carbohydrate activity of the strains, the dbCAN3 server (Zheng et al., 2023) was employed to detect putative carbohydrate-active enzymes (CAZymes) by aligning sequences with the CAZymes database. The identified CAZymes were classified into glycoside hydrolases (GHs), glycosyltransferases (GTs), polysaccharide lyases (PLs), carbohydrate esterases (CEs), auxiliary activities (AAs), and carbohydrate-binding modules (CBMs) based on the CAZy database. This was achieved using HMMER search via dbCAN CAZyme domain HMM database (HMMER/CAZyme), DIAMOND search in the CAZy database (DIAMOND/CAZy), and HMMER search via dbCAN-sub HMM database of CAZyme subfamilies (HMMER/dbCAN-sub). To ensure the reliability of our results, we adopted a consensus approach where only the results predicted by at least two of the three tools were used for further analysis. We use KEGG and SubtiWiki (Zhu and Stülke, 2018) databases to annotate the specific response genes in the carbohydrate degradation pathway.

#### 2.8.2 Prediction of secondary metabolite biosynthetic gene clusters

Biosynthetic gene clusters (BGCs) of all novel strains and reference strain genomes were predicted and annotated by antiSMASH software with version 7.0 (https://antismash.secondarymetabolites.org/) (Blin et al., 2023). We further utilized Biosynthetic Gene Similarity Clustering and Prospecting Engine (BIG-SCAPE) software package to compare the Minimum Information about a Biosynthetic Gene (MIBiG) database for diversity clustering and similarity analysis of the predicted BGCs files (Terlouw et al., 2023).

## 3 Results

### 3.1 Diversity and composition of bacterial communities of SMA and NCA sites in SCS

A total of 1,668,346 reads were obtained from 33 different marine sediment samples in South China Sea (SCS), and 5,267 bacterial amplified sequence variants (ASVs) were obtained for bacterial communities.

**Figure 2:**
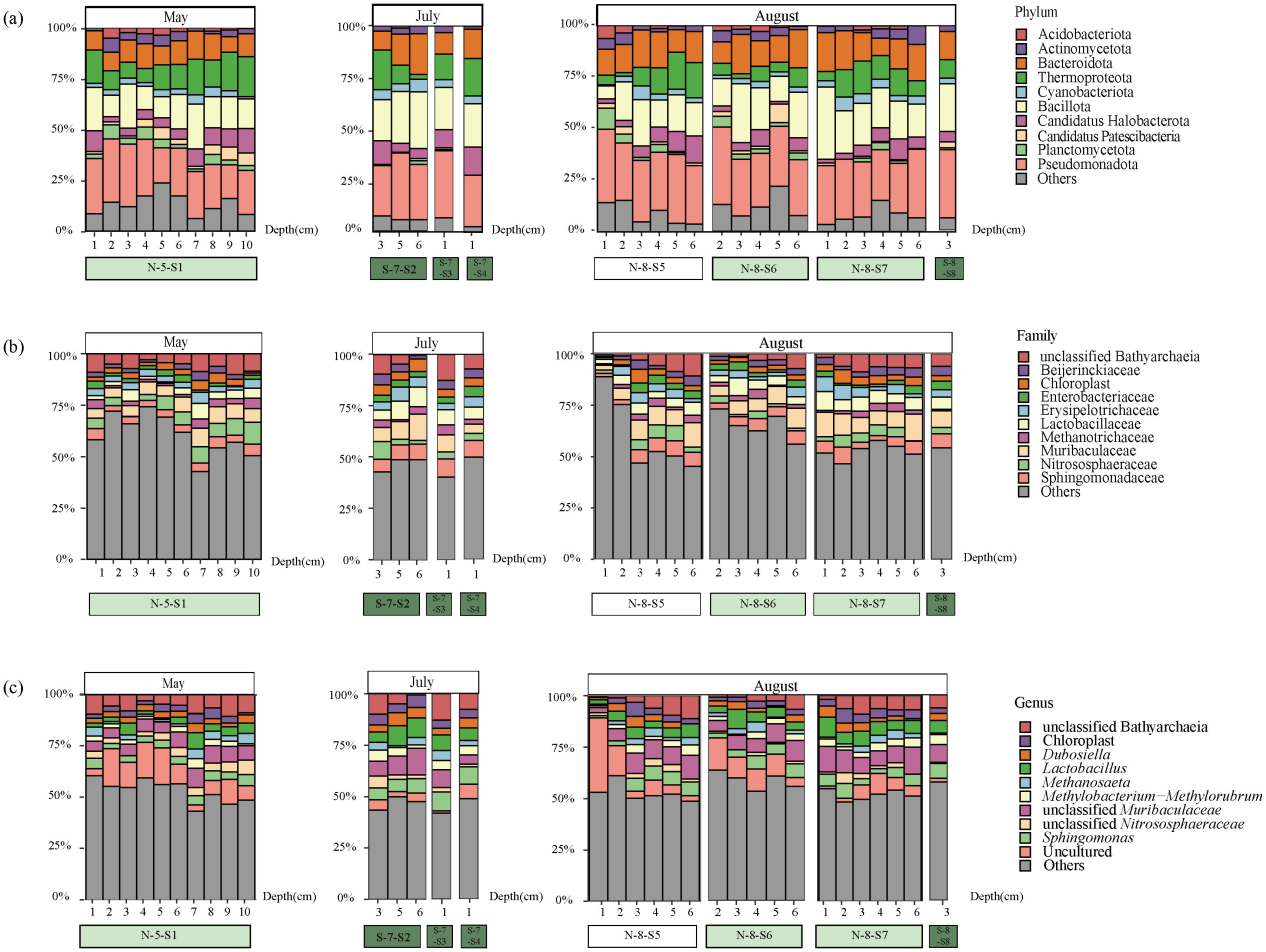
Community composition of major taxonomic groups of bacteria from 16S rRNA gene sequences at various sites and depths in SCS. The top 10 taxonomic groups were displayed, while all others were categorized as “Others.” **(a)** Relative abundance of bacteria at the phylum level; **(b)** Relative abundance of bacteria at the family level; **(c)** Relative abundance of bacteria at the genus level.

**Figure 3:**
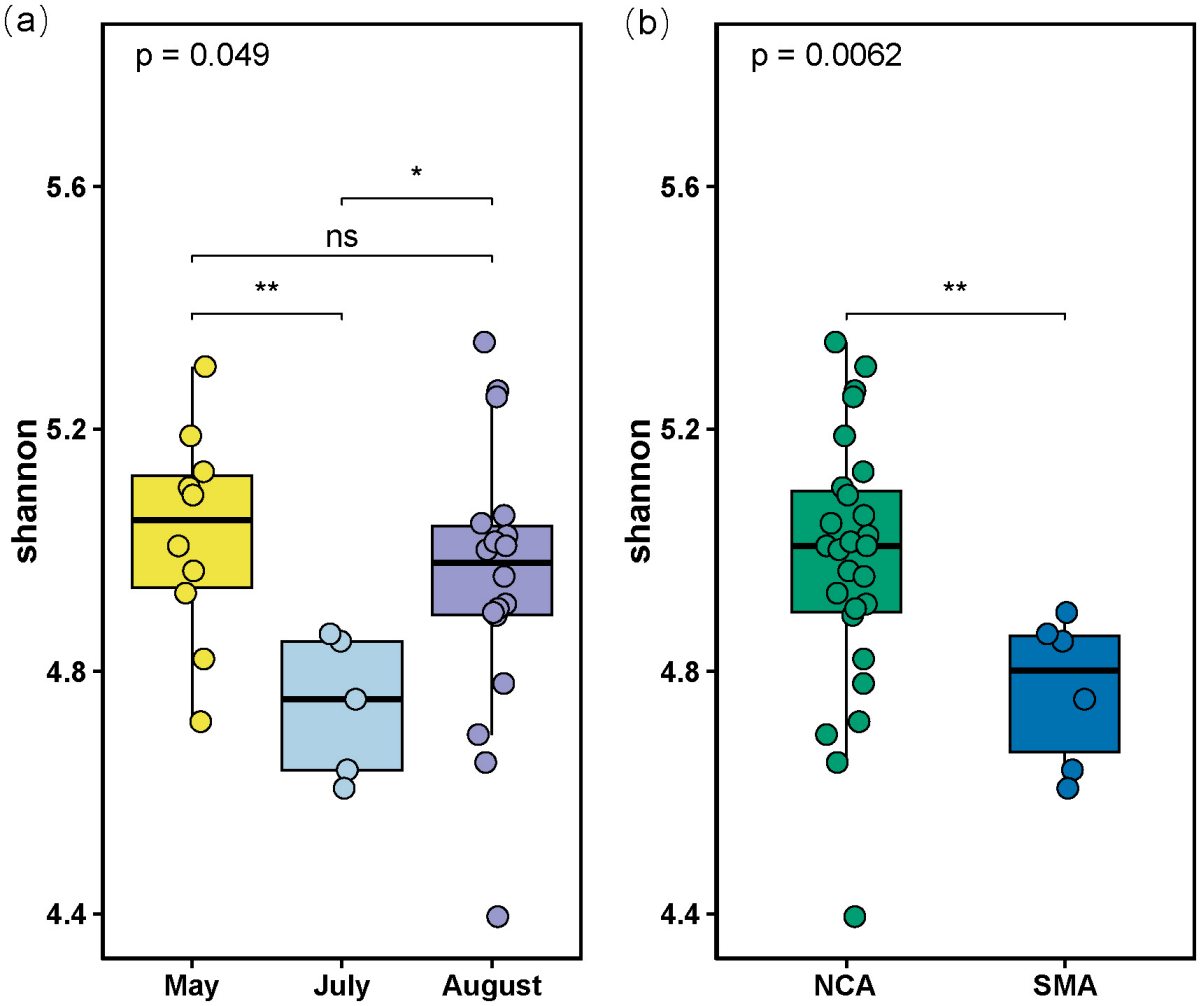
**(a)** Boxplot of Shannon indices calculated using the ASVs data in the 3 Months; **(b)** Boxplot of Shannon indices calculated using the ASVs data in the Southern Mining Area (SMA) and NCA (Northern Mining Area). “ns” stands for no significance where *p*-value > 0.05, “*” stands for *p*-value < 0.05, “**” stands for *p*-value < 0.01.

Aligned by the SILVA version 132 database, all bacterial sequences were affiliated with different taxonomic precision, including 55 phyla, 139 classes, 304 orders, 435 families, and 641 genera. At the phylum level, the bacterial composition was dominated by *Pseudomonadota* (27.3%), *Bacillota* (18.32%), and *Bacteroidota* (13.09%; Figure 2a). In addition, *Cyanobacteriota* showed a significant variation in abundance between SMA and NCA, while *Planctomycetota* showed a decreasing trend with increasing sediment depth. We also retained the top 10 families in terms of abundance, with the others categorized as “Others.” At the family level, *Muribaculaceae* (7.27%), *Bathyarchaeia* (5.49%), and *Sphingomonadaceae* (4.89%) are the most dominant components (Figure 2b). At the genus level, uncultured microorganisms (8.21%) are the most dominant components (Figure 2c), particularly in the N-8-S5 station, where they accounted for 33.41% of the composition. The ASV sequences obtained by sequencing were grouped for diversity comparison. After confirming normal distribution and that the variances followed a chi-square distribution, the *t*-test was used to analyze the differences. The *p*-value of Shannon's index was 0.0062, which was < 0.05, indicating a significant difference between the two, that is, the microbial diversity in the NCA area was significantly higher than that in the SMA area (Figure 3). In addition, the diversity of the sampling results was higher in May and August than in July.

**Figure 4:**
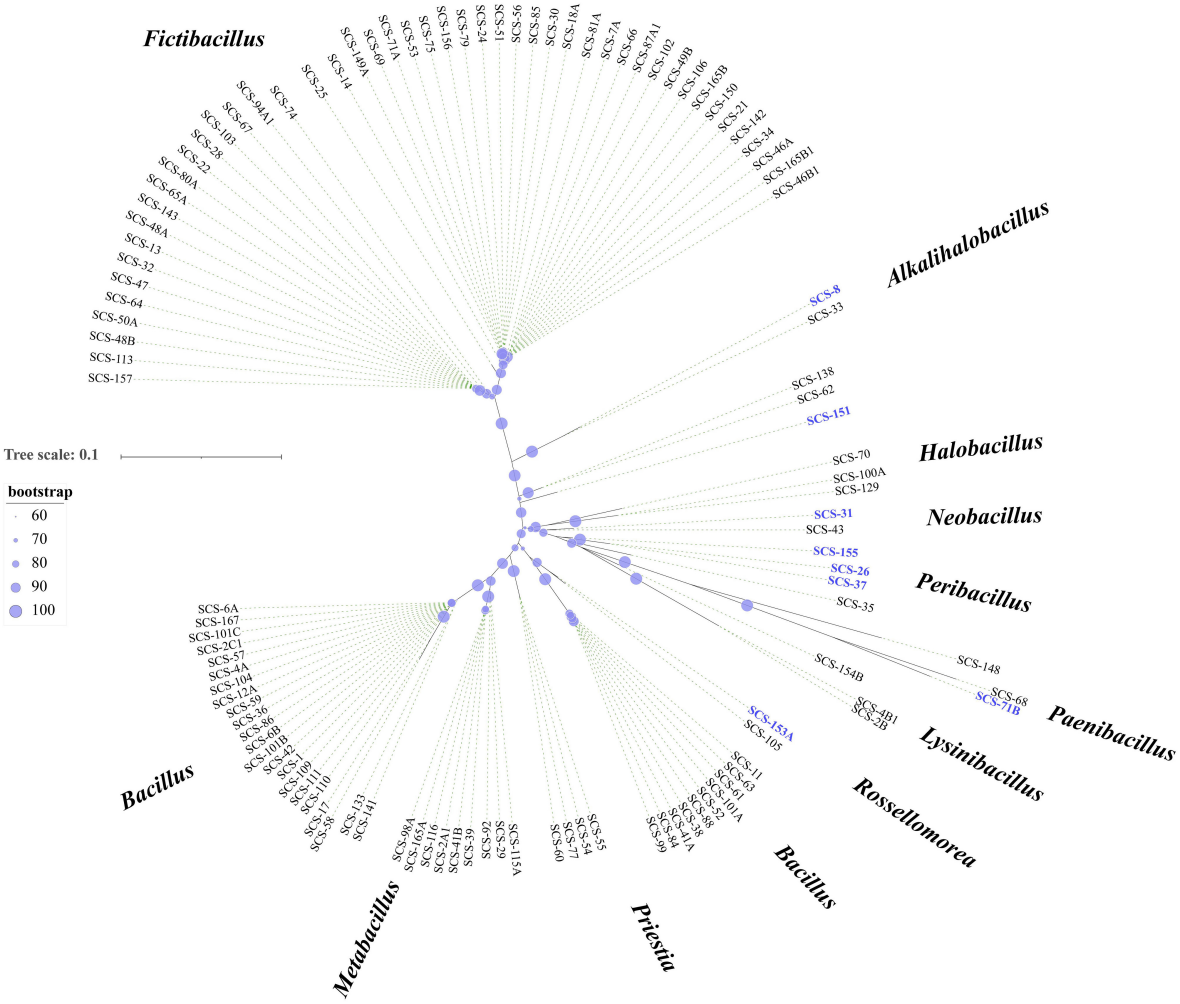
Diversity of cultivable bacteria from sediments. Phylogenetic tree constructed using 16S rRNA gene sequences of all isolates. The tree was constructed using MEGA X software with distance options according to the Kimura two-parameter model and clustering with the Neighbor-Joining (NJ). The size of the blue points on the branch represents the bootstrap value. Evolutionary distances were calculated using the Kimura two-parameter model, with a Bootstrap analysis with 2,000 replications. Bar, 10 nt substitution per 100 nt. Bold text indicates the strains isolated in this study (total, *n* = 156).

The culture-independent approaches could efficiently and rapidly provide overall microbial diversity information. While the cultivable microbes are essential for applied microbiological research. To acquire these microbial resources, we isolated and cultured the strains from these sediment samples. By analyzing bacterial 16S rRNA genes amplified from all isolates, 156 species were obtained for taxonomic identification (at 99% sequence similarity). They belonged to 12 different bacterial genera (Figure 4). Our cultivation strategy enabled the isolation of numerous representatives of previously described genera from marine sediments in the South China Sea (Sun et al., 2008), such as *Fictibacillus, Bacillus, Metabacillus*, and “Others”.

### 3.2 Polyphasic characterization of novel species

Among these isolates, we identified six novel species, designated as strains SCS-8^T^, SCS-26^T^, SCS-31^T^, SCS-37^T^, SCS-153A^T^, and SCS-155^T^, and one novel genus represented by Strain SCS-151^T^. The strains exhibited 16S rRNA gene sequence similarities below 98.65% compared to recognized species in EzBioCloud. Strain SCS-8^T^ showed high 16S rDNA sequence similarity to *Pseudalkalibacillus berkeleyi* KMM 6244^T^ (98.64%). The strains SCS-26^T^ and SCS-37^T^ showed 98.16 and 98.37% similarity to *Peribacillus kribbensis* BT080^T^, respectively. The Strain SCS-31^T^ had 98.47% similarity to *Neobacillus piezotolerans* YLB-04^T^. The Strain SCS-153A^T^ showed high similarity to *Bacillus salacetis* SKP7-4^T^ (98.88%), the Strain SCS-155^T^ had 98.03% similarity to *Bacillus massiliglaciei* Marseille-P2600^T^, and the Strain SCS-151^T^ shared 96.95% similarity with *Cytobacillus citreus* FJAT-49705^T^.

**Figure 5:**
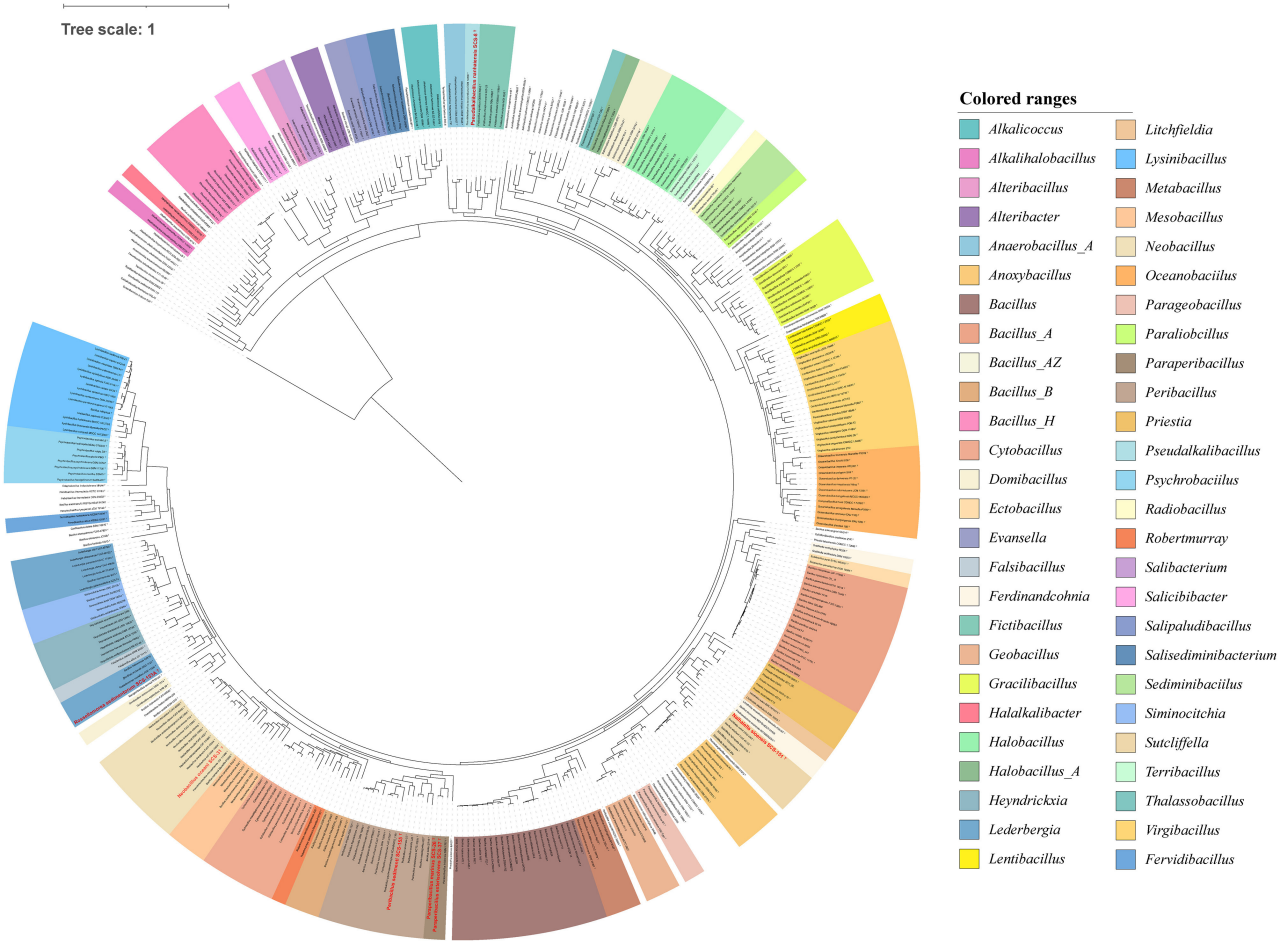
Maximum likelihood phylogenetic tree of *Bacillaceae* based on 120 bacterial marker genes. The phylogenomic tree was reconstructed using the GTDB-tk tool. The red frames represent novel species of *Bacillaceae* isolated in this study.

The Maximum-Likelihood (ML) phylogenetic trees conducted by 16S rRNA gene sequences of these strains were shown in Supplementary Figures S1a–f, and the topological structures were identical by the NJ method (shown in filled circle). These clades were further found to be stable when the phylogenomic trees were reconstructed using the 92 housekeeping core genes by the UBCG tool in Supplementary Figures S2a–f. The Strain SCS-8^T^ was clustered with the members of the genus *Pseudalkalibacillus*. The Strain SCS-31^T^ was clustered with *Neobacillus* genus. The strains SCS-26^T^, SCS-37^T^, and SCS-155^T^ were clustered with the *Peribacillus* clade. Based on the phylogenomic tree of strains SCS-26^T^, SCS-37^T^, and SCS-155^T^, and their reference *Peribacillus* species (Supplementary Figure S2c), strains SCS-26^T^ and SCS-37^T^ were clustered with *Peribacillus deserti* and *P. kribbensis* to form a new clade (named the *Paraperibacillus* genus) with 76% bootstrap support. The Strain SCS-155^T^ still belongs to the *Peribacillus* genus. The Strain SCS-153A^T^ was clustered with *B. salacetis*, which can be reclassified into the genus *Rossellomorea*. The Strain SCS-151^T^ forms a separate branch distinct from the listed genera. These results were consistent with the ML phylogenomic tree, obtained using the GTDB-tk tool based on 120 bacterial marker genes (Figure 5).

These phylogenies of the novel strains could be complemented with the OGRIs. For nucleotide-based OGRIs, the ANI values between Strain SCS-8^T^ and its related taxon were range from 71 to 75%, and other strains showed the following ranges with their respective related taxon: Strains SCS-26^T^ (71%−74%), SCS-31^T^ (73%−85%), SCS-37^T^ (71%−74%), SCS-151^T^ (72%−73%), SCS-153A^T^ (72%−79%), and SCS-155^T^ (72%−74%). The dDDH values between novel strains and their respective related taxon were as follows: Strains SCS-8^T^ (19.1%−28.4%), SCS-26^T^ (20.1%−28%), SCS-31^T^ (21.1%−28.4%), SCS-37^T^ (20.2%−26.3%), SCS-151^T^ (23.9%−33.6%), SCS-153A^T^ (21.6%−23.8%), and SCS-155^T^ (22.1%−26.6%; Supplementary Table S4).

For protein-based OGRIs, the AAI values between novel strains and their respective related taxon were as follows: Strain SCS-8^T^ exhibited values below 79.81% compared to its reference strains; Strain SCS-26^T^, below 76.04%; Strain SCS-31^T^, below 88.98%; Strain SCS-37^T^, below 76.02%; Strain SCS-151^T^, below 67.00%; Strain SCS-153A^T^, below 84.00%; and Strain SCS-155^T^, below 73.83% (Supplementary Tables S5a–e). These ANI, dDDH, and AAI values were all lower than the species' threshold (ANI 95%, dDDH 70%, and AAI 95%, respectively), indicating these strains belong to novel species. Besides, the AAI value of strains SCS-26^T^ and SCS-37^T^ with *P. deserti* and *P. kribbensis* was 73.21%−76.04%, whereas those with other *Peribacillus* species were 65.09%−69.19% (Supplementary Table S6b). This evidence also supports that the original *Peribacillus* clade can be divided into two genera as follows: *Paraperbacillus* and *Peribacillus*. The AAI values between Strain SCS-151^T^ and these genera are lower than their intragenus AAI values, ranging from 66.33 to 67.00% for *Lichfieldia* (intragenus 69.10%−77.89%), 64.81%−65.06% for *Sutcliffiella* (intragenus 70.20%−81.23%), and 66.24%−66.56% for *Ferdinandcohnia* (intragenus 86.10%−94.06%; Supplementary Table S6d). These results support the taxonomic position of Strain SCS-151^T^ as a novel species of a new genus, for which the name *Nanhaiella sioensis* gen. nov., sp. nov. is proposed.

Based on the phylogenomic tree and OGRIs analysis, several strains have updated their taxonomic status. Strains SCS-26^T^ and SCS-37^T^, *P. kribbensis*, and *P. deserti* have been reclassified into the novel genus *Paraperibacillus*. *B. salacetis* has been reclassified into the genus *Rossellomorea*, now named *Rossellomorea salacetis*. The Strain SCS-151^T^ has been proposed as a novel species of a new genus.

The low 16S rRNA gene sequence similarity, the phylogenetic and phylogenomic trees, and OGRIs indicated that they should represent novel species of different genera. Their morphological, physiological, and chemotaxonomic characteristics were analyzed to further clarify the taxonomic positions of these novel strains. The novel species are all rod shaped under optimal growth conditions and Gram-stain positive. Cells of these novel strains are motile except for the Strain SCS-8^T^. In the *Pseudalkalibacillus* clade, Strain SCS-8^T^ lacks catalase activity and cannot grow below 20°C. In the *Paraperibacillus* clade, strains SCS-26^T^ and SCS-37^T^ can hydrolyze β-galactosidase. In the *Neobacillus* clade, Strain SCS-31^T^ tolerates a wider NaCl range (0%−8%). In the *Rossellomorea* clade, Strain SCS-153A^T^ has positive oxidase activity. These different polyphasic characteristics can distinguish them from their reference strains. Detailed polyphasic characterizations are listed in Supplementary Tables S7a–e.

### 3.3 Comparative genomic analysis of seven novel strains

To comprehensively analyze the gene function defects or specificity of the strains in this study compared with the available strains from a genomic perspective, we chose appropriate reference strains under these six genus-level classification units based on the GTDB phylogenomic tree results. We collected the genomes of the reference strains that had been sequenced from the NCBI RefSeq database, including the genomes of four species of *Pseudalkalibacillus*, three genomes under the genus *Peribacillus*, two genomes under the genus *Paraperibacillus*, four genomes under the genus *Neobacillus*, two genomes under the genus *Cytobacillus*, four genomes under the genus *Rossellomorea*, and two reference genomes under *Bacillus*, totaling 21 reference genome sequences. The genome size, GC%, number of tRNAs, number of rRNAs, and other genetic structures of 28 strains are shown in Table 1.

According to the comparison, the genome size and the number of CDSs in strains SCS-8^T^, SCS-26^T^, SCS-31^T^, SCS-37^T^, and SCS-153A^T^ are lower than the average values of their respective clade. For example, in the *Pseudalkalibacillus* clade, Strain SCS-8^T^ had a genome size of 3.62 Mb, which was lower than the average genome size (4.46 Mb). A total of 3,669 CDSs were annotated in Strain SCS-8^T^, which was also lower than the average value (4,406) in this clade. It reported that marine microbes tend to have smaller genome sizes than terrestrial species (Chuckran et al., 2022; Lerat et al., 2005; Rodríguez-Gijón et al., 2022). This reduction in genome size may be an adaptation to the marine environment, where resources are relatively limited, and streamlining the genome could provide a selective advantage. We have also observed the smaller genome size in our novel species, which may reflect an adaptive evolutionary genome strategy in response to the deep-sea environment.

The proportions of core, accessory, and specific genes in these 28 genomic taxa were subsequently analyzed. “Core genes” were contained by both new species and reference strains; “specific genes” were contained by a single strain only; and “accessory genes” were genomes of two or more, but not all, strains. Core genes are mainly housekeeping genes, which are often associated with crucial biological functions of the species. In contrast, specific genes generally reveal the potential for specific physiological functions of the species. The results of the comparative genome analysis are shown in Table 2. As a result, within the *Pseudalkalibacillus* clade, 13.79%−18.92% (684) gene families were identified as core genes. The *Peribacillus* exhibited 23.31%−26.90% (1,122) core genes among four strains. The *Paraperibacillus* clades exhibited 36.7%−42.91% (1,792) core genes. The *Neobacillus* clade has 14.47%−17.45% (776) core genes among five species. The *Cytobacillus* clade has 13.92%−15.73% (721) core genes among five species. The *Rossellomorea* clade displayed 27.56%−34.97% (1,320) core genes among five strains. The reduced proportion of core genes across these clades is consistent with a previous study of the *Bacillaceae* family (Alcaraz et al., 2010), indicating a higher percentage of accessory and strain-specific genes. The expanded accessory genes correlate to the diverse environments (soil, seawater, sea urchins, etc.) that are encountered by these 28 strains. It confirms the genomic adaptive strategies of *Bacillaceae* lineages, where variable accessory genes are predominantly linked to adaptations for coping with different environmental stresses (Fajardo-Cavazos et al., 2014).

**Table 2:** Core genes, accessory genes, and specific genes information of candidate novel *Bacillaceae* sp. and reference strains.

**Strain**	**Core genes**	**Accessory genes**	**Strain-specific genes**	**Total genes**
Strain SCS-8^T^	684 (18.64%)	1,689 (46.03%)	1,296 (35.32%)	3,669
*Pseudalkalibacillus decolorationis* DSM 14890^T^	684 (13.79%)	1,174 (23.66%)	3,103 (62.55%)	4,961
*Pseudalkalibacillus caeni* HB172195^T^	684 (14.55%)	701 (14.91%)	3,317 (70.54%)	4,702
*Pseudalkalibacillus hwajinpoensis* MABIK MI00000821^T^	684 (15.73%)	650 (14.95%)	3,014 (69.32%)	4,348
*Pseudalkalibacillus berkeleyi* KCTC 12718^T^	684 (18.92%)	1,704 (47.14%)	1,227 (33.94%)	3,615
Strain SCS-26^T^	1,792 (41.35%)	1,876 (43.29%)	666 (15.36%)	4,334
Strain SCS-37^T^	1,792 (42.91%)	1,197 (28.66%)	467 (11.13%)	4,176
*Paraperibacillus kribbensis* DSM 17871^T^	1,792 (36.70%)	460 (9.42%)	2,631 (53.88%)	4,883
*Paraperibacillus deserti* DSM 105482^T^	1,792 (42.60%)	524 (12.46%)	1,891 (44.95%)	4,207
Strain SCS-155^T^	1,122 (23.31%)	955 (19.84%)	2,736 (56.85%)	4,813
*Peribacillus asahii* MA001^T^	1,122 (26.90%)	434 (10.41%)	2,615 (62.69%)	4,171
*Peribacillus glennii* V44-8^T^	1,122 (25.79%)	1,332 (30.61%)	1,897 (43.60%)	4,351
*Peribacillus cavernae* L5^T^	1,122 (25.27%)	1,224 (27.57%)	2,094 (47.16%)	4,440
Strain SCS-31^T^	776 (17.12%)	1,684 (37.14%)	2,074 (45.74%)	4,534
*Neobacillus novalis* FJAT-14227^T^	776 (14.47%)	1,411 (26.31%)	3,175 (59.21%)	5,362
*Neobacillus soli* DSM 15604^T^	776 (14.54%)	1,380 (25.85%)	3,182 (59.61%)	5,338
*Neobacillus notoginsengisoli* JCM 30743^T^	776 (16.58%)	1,031 (22.03%)	2,874 (61.40%)	4,681
*Neobacillus piezotolerans* YLB-04^T^	776 (17.45%)	1,698 (38.18%)	1,973 (44.37%)	4,447
Strain SCS-151^T^	721 (15.57%)	1,463 (31.60%)	2,446 (52.83%)	4,630
*Bacillus massiliigabonensis* Marseille-P2639^T^	721 (14.18%)	768 (15.11%)	3,595 (70.71%)	5,084
*Bacillus timonensis* MGYG-HGUT-01405^T^	721 (15.73%)	698 (15.23%)	3,164 (69.04%)	4,583
*Cytobacillus depressus* BZ1^T^	721 (13.92%)	1,991 (38.44%)	2,467 (47.63%)	5,179
*Cytobacillus citreus* FJAT-49705^T^	721 (14.20%)	2,158 (42.50%)	2,199 (43.30%)	5,078
Strain SCS-153A^T^	1,320 (33.36%)	1,510 (38.16%)	1,127 (28.48%)	3,957
*Rossellomorea vietnamensis* 151-6^T^	1,320 (27.56%)	1,510 (31.53%)	1,959 (40.91%)	4,789
*Rossellomorea salacetis* SKP7-4_1^T^	1,320 (28.60%)	1,576 (34.14%)	1,720 (37.26%)	4,616
*Rossellomorea aquimaris* TF-12^T^	1,320 (34.97%)	1,251 (33.14%)	1,204 (31.89%)	3,775
*Rossellomorea marisflavi* JCM 11544^T^	1,320 (30.43%)	722 (16.64%)	2,296 (52.93%)	4,338

To understand the main functional categories of core and strain-specific genes, we subsequently performed COG annotation on the open reading frames (ORFs) obtained from the 28 strains. The COG functional genes were classified into core COG functional genes, accessory COG functional genes, and strain-specific COG functional genes. In the *Pseualkalihalobacillus* clade (Supplementary Figure S8a), we found that the core genes of Strain SCS-8^T^ contain 684 genes, accounting for 18.64% of the total gene count. In comparison, the accessory genes and strain-specific genes include 1,689 genes (46.03%) and 1,296 genes (35.32%), respectively. Mapping the core, accessory, and strain-specific genes to the COG database, revealing that the core COG genes are predominantly associated with essential metabolic functions, with COG J (translation, ribosomal structure, and biogenesis) and COG L (replication, recombination, and repair) being the most represented categories. Besides, strain-specific genes in Strain SCS-8^T^ are primarily classified under COG categories M (cell wall/membrane/envelope biogenesis) and V (defense mechanisms). The COG category M is implicated in preserving cellular structural integrity, crucial in high-pressure, high-salt environments. There is no strain-specific gene annotated with the COG categories Z (related to the cytoskeleton), U (related to intracellular trafficking, secretion, and vesicular transport), and W (related to extracellular structures).

In the *Peribacillus* and *Paraperibacillus* clades, Strain SCS-155^T^ had the most strain-specific genes. The number of strain-specific genes in strains SCS-26^T^ and SCS-37^T^ was relatively low (Supplementary Figures S8b, c), making them the strains with the lowest proportion of strain-specific genes. This phenomenon may be due to the high similarity between SCS-26^T^ and SCS-37^T^, as they share 640 genes. The COG analysis of strain-specific genes for these three strains focused on COG X (representing prophages and transposons), COG Q (representing secondary metabolite biosynthesis, transport, and metabolism), and COG M categories (Strain SCS-26^T^: COG X and COG V; Strain SCS-37^T^: COG X, COG Q, and COG M; and Strain SCS-155^T^: COG X and COG M). The genes associated with the COG X category are commonly linked to prophages, transposons, and other mobile genetic elements (MGE). The COG X category had a higher proportion of the strain-specific genes of strains SCS-26^T^, SCS-37^T^, and SCS-155^T^. The findings indicate that these three novel strains contain unique MGEs, which likely play a role in horizontal gene transfer (HGT) in the deep marine sediment environments.

In the *Neobacillus* clade, Strain SCS-31^T^ possesses 2,074 unique genes (45.74%; Supplementary Figure S8c). In particular, there is a high percentage of specific genes in COG G (carbohydrate transport and metabolism) and E (amino acid transport and metabolism) categories, highlighting the unique utilization of carbohydrate and amino acid resources in Strain SCS-31^T^. In *Cytobacillus* clade, we found the Strain SCS-151^T^ has 3585 specific genes, accounting for 52.83% of its total genome (Supplementary Figure S8d). The main differences between strain SCS-151^T^ and other reference strains were concentrated in the COG X, V, Q, and U. In *Rossellomorea* clade, Strain SCS-153A^T^ showed 1127 specific genes (28.48%), which made it the lowest number of specific genes among the seven novel strains (Supplementary Figure S8e). These specific genes showed a high percentage in COG X and G categories.

The higher proportion of specific genes may suggest that the novel species has the potential to encode novel functional proteins. We analyzed the strain-specific genes of novel species based on the COG database, focusing on the COG M, Q, V, and G (carbohydrate metabolism) categories. Due to the presence of isozymes, the analysis of the different protein functions relying only on COG classification may not reflect comprehensively and adequately. To further explore the functional differences and specificity within carbohydrate metabolism, secondary biosynthetic products, and membrane compositions, we annotated these genomes with the CAZyme, antiSMASH, and other databases. These results could help us understand metabolic processes, signaling, and other unique biological functions of the novel species.

#### 3.3.1 Degradation of carbohydrate

**Figure 6:**
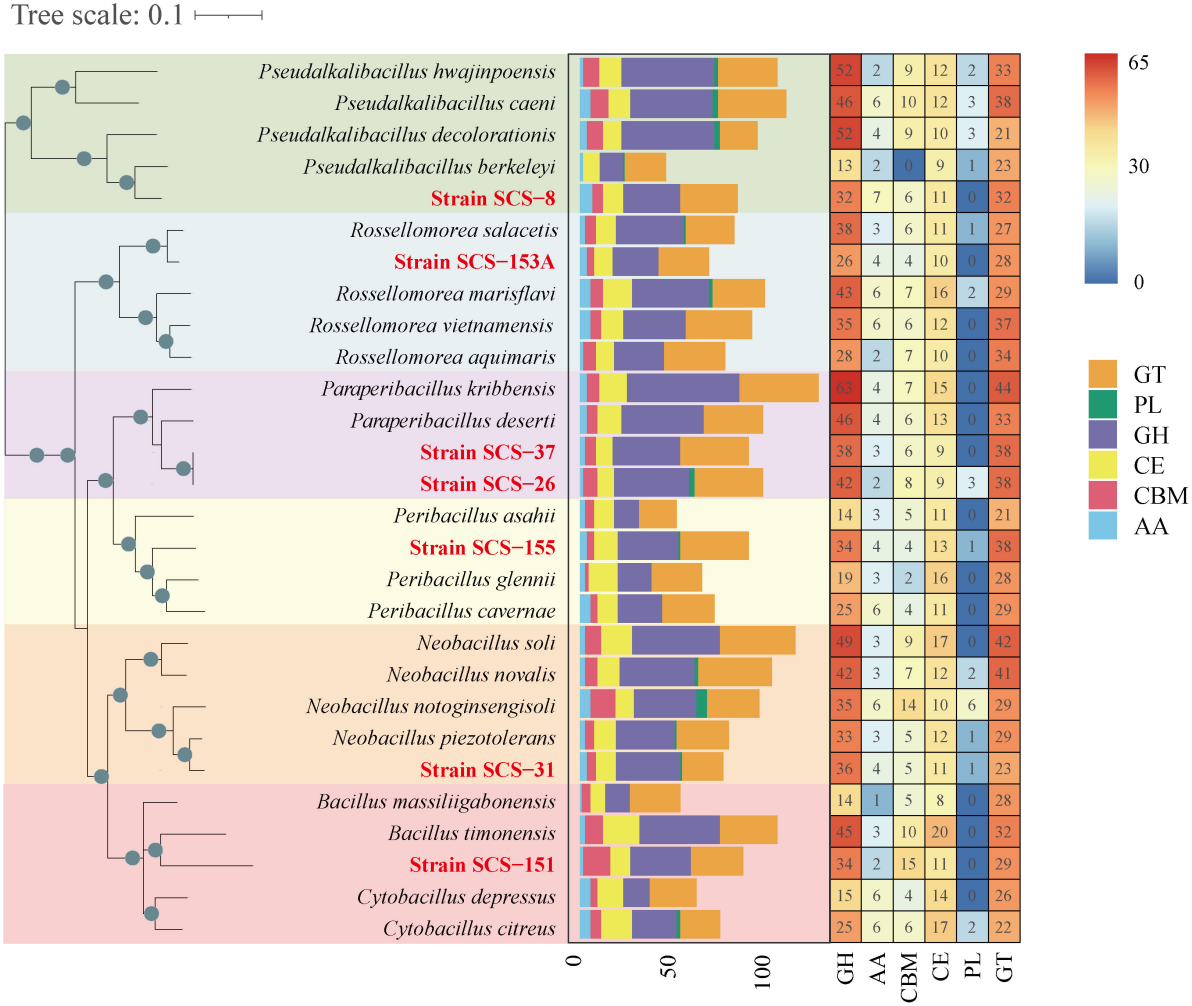
Heatmap of CAZyme family among different strains on the left. The abundance of different CAZyme families within 28 strains is shown on the right. Glycoside hydrolases (GHs), glycosyltransferases (GTs), polysaccharide lyases (PLs), carbohydrate esterases (CEs), auxiliary activities (AAs), and carbohydrate-binding modules (CBMs). The seven strains in this study are red in color.

Carbohydrate metabolic genomic analysis was employed on CAZymes, KEGG, and SubtiWiki databases. As a result, a total of 88, 102, 80, 94, 91, 72, and 94 CAZymes were identified in strains SCS-8^T^, SCS-26^T^, SCS-31^T^, SCS-37^T^, SCS-151^T^, SCS-153A^T^, and SCS-155^T^, respectively (Figure 6). These values could represent their capacity for carbohydrate metabolism of novel species in marine sediment. Among the analyzed species, SCS-151^T^ showed lower CAZymes than other novel species, while SCS-26^T^ showed a notably higher number of CAZymes. We conducted a further analysis of the abundance of different CAZyme families, finding that glycoside hydrolases (GHs) enzymes had the highest abundance. GH enzymes have glycosidic bond hydrolysis and rearrangement and are usually involved in the process of sugar hydrolysis, playing a role in cell wall degradation, the digestion of sugars, and a variety of biochemical pathways.

**Figure 7:**
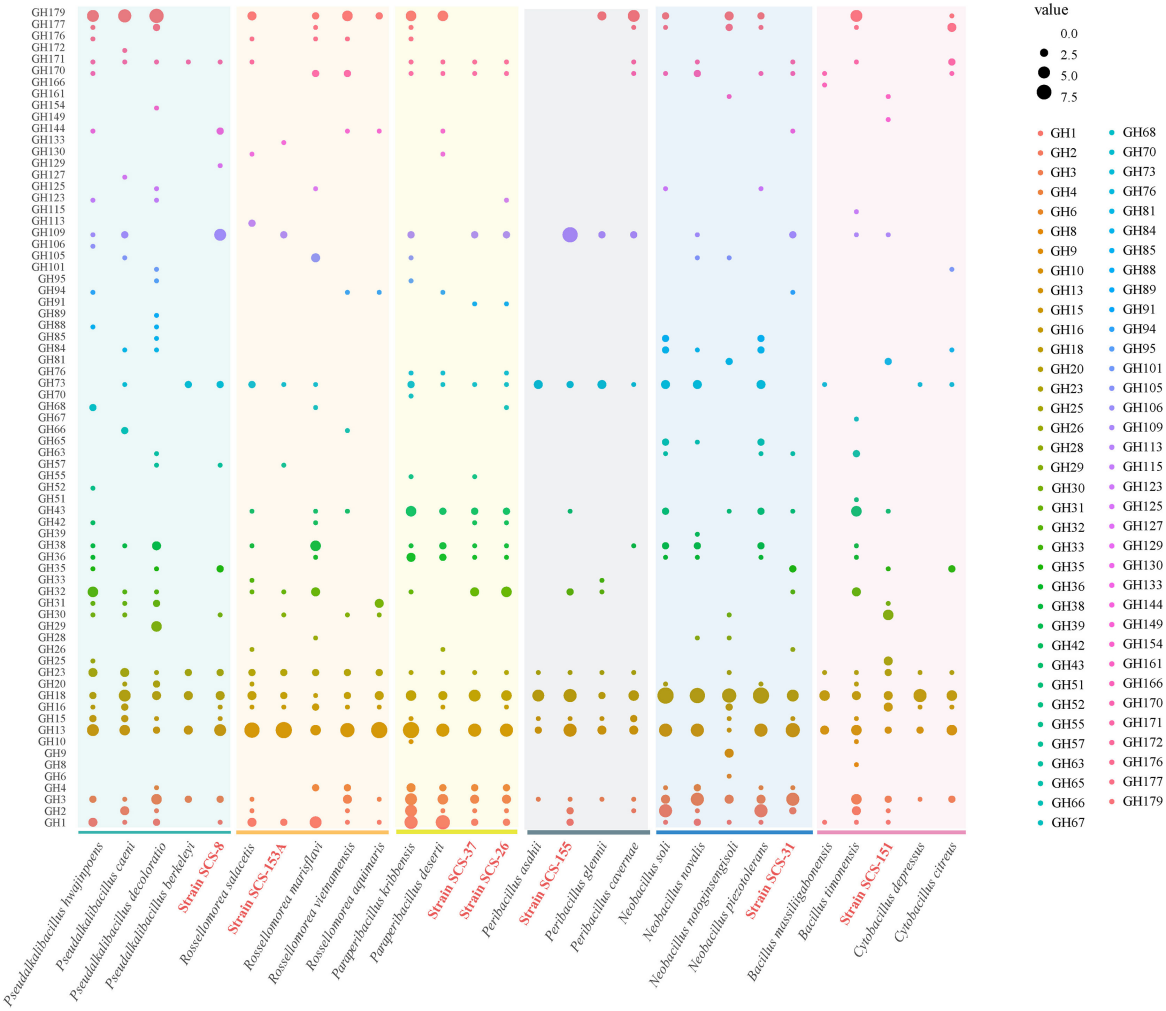
Domain distribution of GH family among different strains of the six clades. The seven novel strains in this study are red in color.

Furthermore, we extended the analysis of the abundance of diverse families that consist of GH categories. The results suggest that the GH distribution of the novel species of *Bacillaceae* differs from the available reference strains (Figure 7). We found that all novel species lacked the genes annotated to the GH177 and GH179 families, and there was a high abundance of genes annotated to the GH109 family. In the previous study, the GH109 family was related to the hydrolysis of the O-glycan chain of mucin (Liu et al., 2007). GH179, a recently defined GH family, possesses β-N-acetylglucosidase activity, which shares a similar N-terminal NAD+ structural domain with GH109. In the *Pseudalkalibacillus* clade, Strain SCS-8^T^ possesses genes annotated to the GH129 family, which is different from other reference strains. The GH129 family is known for its involvement in the degradation of algal polysaccharides in marine environments, potentially reducing algal bloom biomass accumulation and influencing oceanic carbon turnover (Kiyohara et al., 2012). In the *Rossellomorea* clade, Strain SCS-153A^T^ possesses genes annotated to GH133 family (glycogen exo-alpha-1,6-glucosidase) and lacks GH176 family (isoamylase) (Wang et al., 2022), which may indicate a substrate utilization preference. Among the *Paraperibacillus* clade, strains SCS-26^T^ and SCS-37^T^ have genes in the GH91 and GH32 families, and these two families are associated with the catabolism of inulin in fructans. In the *Neobacillus* clade, Strain SCS-31^T^ lacks the GH1 family and contains the GH32 and GH35 families, which are associated with the hydrolysis of exo-glucosidase in chitin. Compared with the *B. songklensis, B. fengqiuensis*, and *Cytobacillus* clade, Strain SCS-151^T^ exhibited a distinct GH family profile, especially with more genes of the GH30 family. The enzymes from the GH30 family play a key role in the metabolism of xylan and pectin (St John et al., 2010), which could bridge carbon flow between plant biomass and microbial food webs.

Additionally, we annotated these genomes using the KEGG and SubtiWiki databases, focusing on the genes involved in the metabolic utilization of carbohydrates. First, based on KEEG annotations, the phosphotransferase system (PTS) and ABC transporter genes associated with inward transport of carbohydrates, as well as glycolysis and pentose phosphate pathway (PRPP) genes associated with carbon domain utilization pathways, were obtained.

**Figure 8:**
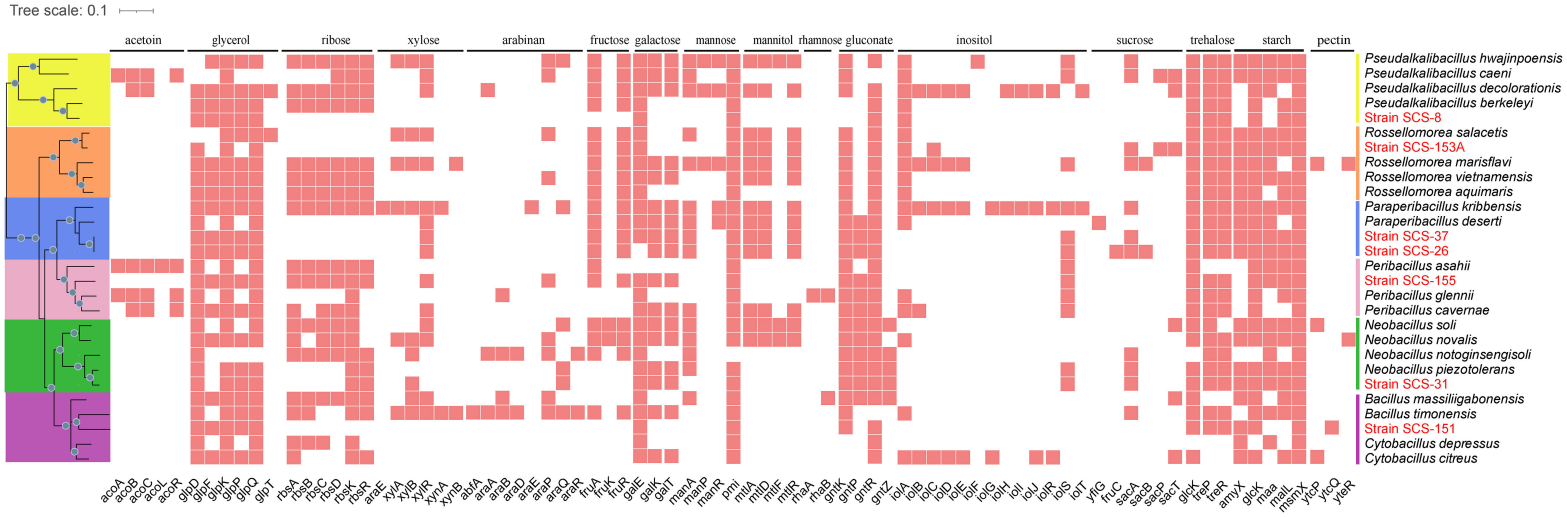
Reconstruction of metabolic features found in the genomes of the seven novel strains and the other 21 reference species. Symport, antiport, uniport, and direction are indicated by the number and direction of arrows. Gray points indicate that the metabolic pathway is present both in the API 50CHB experiment and in the genetic pathway annotated by KEGG results.

In Figure 8, the metabolic pathways of the novel strains and type strains are presented. Filled triangles indicate the presence of genes annotated in the KEGG database for these strains. Filled black dots represent the confirmed utilization of carbohydrates as verified by the API 50CHB strip experiment. In the *Pseudalkalibacillus* clade, the PTS, which is only related to glucose and fructose basic monosaccharides, was annotated in Strain SCS-8^T^. It is suggested that Strain SCS-8^T^ has the potential to metabolize these basic monosaccharides. Compared with other reference strains, except for *P. berkeleyi*, mannose, mannitol, and sucrose PTS were absent in Strain SCS-8^T^. This result suggests that Strain SCS-8^T^ may have lost a large number of genes related to carbohydrate utilization during evolutionary processing. In *Paraperibacillus* clade, the Strain SCS-26^T^ was annotated to be PTS-associated with glucose, fructose, maltose, mannitol, N-acetyl-glucosamine, trehalose, and sucrose, as well as ABC transporter genes associated with ribose, rhamnose, and galactose. In comparison with the reference strains, SCS-26^T^ could utilize more extensive substrates. Compared to Strain SCS-26^T^, the Strain SCS-37^T^ was missing PTS genes for maltose, β-glucoside, and ribose, and in the *Peribacillus* clade, the Strain SCS-155^T^ was missing PTS genes for maltose, β-glucoside, and mannitol. In the *Neobacillus* clade, Strain SCS-31^T^ lacks the genes associated with transporting key carbon sources into the cell, including PTS genes for glucose, fructose, and mannose. However, the results of the API 50CHB experiment revealed more diverse carbohydrate substrate utilization traits. It suggests that certain putative proteins may perform similar carbohydrate transport functions. Additionally, Strain SCS-151^T^ exhibited very low carbon source utilization activity, whereas Strain SCS-153A^T^ demonstrated a broad range of carbon substrate utilization traits. In conclusion, their annotation results were well-aligned with the findings from the API 50CHB strips.

There are 16 carbon sources that were selected according to the carbohydrate utilization experiment of the polyphasic approach. These carbon sources include acetoin, glycerol, ribose, xylose, arabinan, fructose, galactose, mannose, mannitol, rhamnose, gluconate, inositol, sucrose, trehalose, starch, and pectin. The gene names related to the degradation pathways of these carbon sources were obtained from the SubtiWiki website. After matching our annotation to the gene name list from the SubtiWiki website, the different results in the presence or absence of genes in metabolic pathways across these 28 species remained.

**Figure 9:**
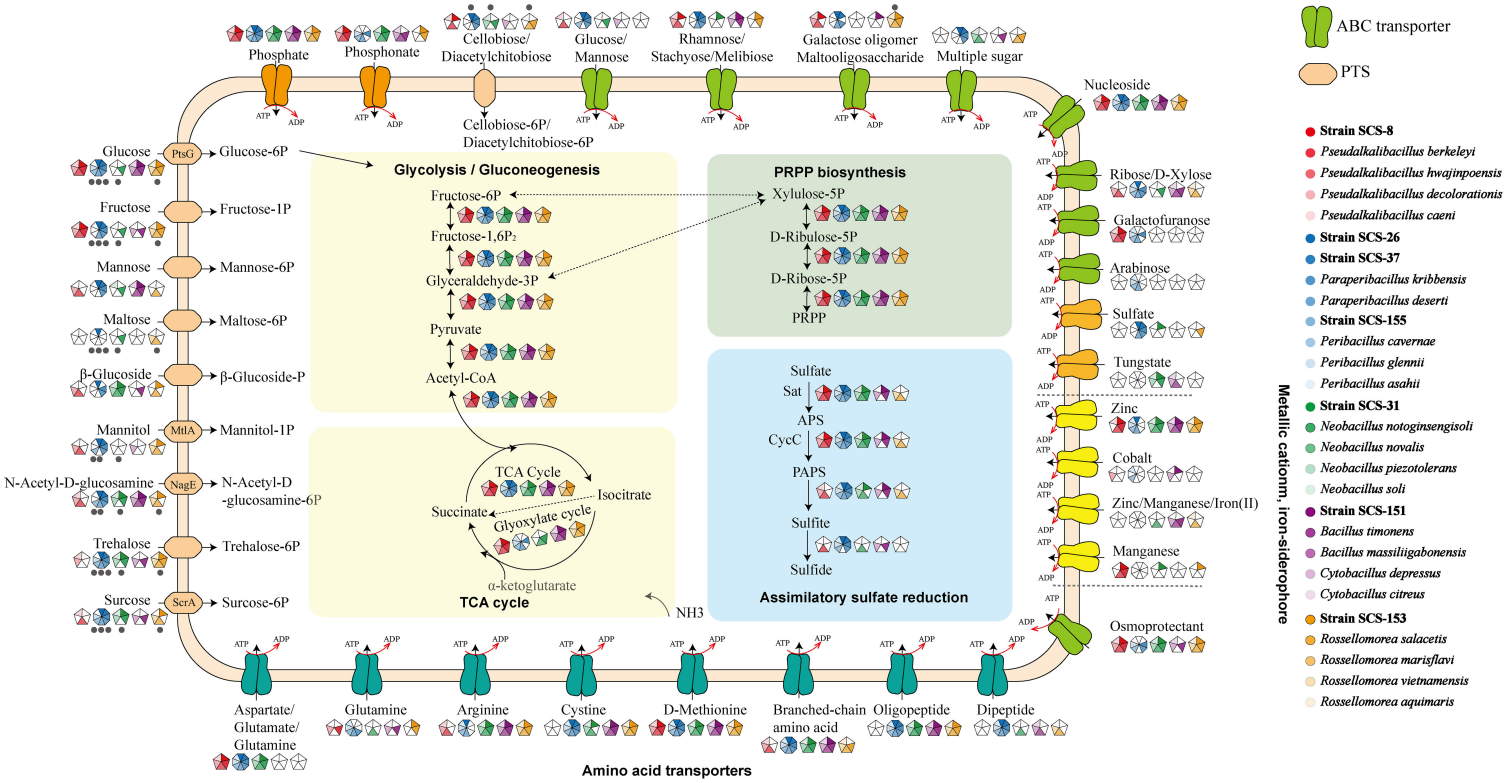
Distribution of specific carbohydrate substrate metabolism genes in 28 strains of six clades. The seven strains in this study are red in color. The different results of the respective clades were retained, and the consistent results were removed.

As a result, we found that these strains exhibit different genes in the specific carbon source utilization (Figure 9). Compared with other reference strains, all seven novel strains showed a lower number of genes related to the metabolism of xylose and arabinose, which are both major components of plant cell walls. It is reasonable to infer that in deep-sea sediments, sedentary microbial taxa may have streamlined some genes associated with plant polysaccharide degradation during the evolutionary process due to the general lack of organic carbon sources for plant fibers on land. In particular, in the *Pseudalkalibacillus* clade, the Strain SCS-8^T^ is missing several genes associated with ribose (*rbsA, rbsB, rbsC, rbsD, rbsK*, and *rbsR*), xylose (*xylA, xylB, xylR, xynA, xynB*, and *xynP*), arabinose (*abnA, araA, araB, araD, araE, araN, araP*, and *araQ*), and rhamnose (*manA, manP*, and *manR*). As mentioned above, the genome size and the number of its CDSs of Strain SCS-8^T^ are smaller than those of the other reference strains, suggesting that genome streamlining may have occurred in Strain SCS-8^T^. This phenomenon may be an evolutionary strategy for adaptation to the deep-sea environment with nutrient limitation or specific ecological niches. In addition, Strain SCS-151^T^ had a large number of missing genes (compared with the other four reference strains), related to ribose, xylose, arabinan, fructose, mannitol, rhamnose, and sucrose. The results suggest that Strain SCS-151^T^ follows a streamlined set of carbohydrate utilization pathways, selectively retaining metabolic pathways for the carbon sources that are prevalent in its environment. However, strains SCS-26^T^ and SCS-37^T^ of the *Paraperibacillus* genus exhibited a higher conservation of genes involved in carbon source utilization. These results suggest that these two novel species have retained a comprehensive suite of metabolic pathways, enabling them to adapt to a variety of environmental conditions.

#### 3.3.2 Prediction of biosynthetic products

BGCs are crucial for producing diverse secondary metabolites, which facilitate interference competition among microbes (Steinke et al., 2021). In this study, we analyzed the genomes of seven novel strains and predicted their secondary metabolite types using antiSMASH software. The cluster details are presented in Table 3: there are six BGCs predicted in Strain SCS-8^T^; five and four BGCs were in strains SCS-37^T^ and SCS-26^T^, respectively, and only one BGC was detected in Strain SCS-151^T^. The BGC prediction detected a total of 26 BGCs of these novel species, corresponding to an average of 3.7 BGCs per genome. Previous studies show that the average genome of the *Bacillaceae* family encodes 12.2 BGCs (Yin et al., 2023), which is higher than the novel species in this study. The different numbers of BGCs of different *Bacillaceae* clades showed they have distinct physiological traits and specialized niche adaptation strategies (Xia et al., 2022).

**Table 3:** BGC prediction of seven novel strains.

**Strain**	**Region**	**Type**	**From**	**To**	**Most similar known cluster**	**Similarity (%)**
Strain SCS-8^T^	Region 1	NRPS, transAT-PKS	547,282	619,263	Bottromycin A2	RiPP:bottromycin (6%)
	Region 2	NI-siderophore	923,108	937,093		
	Region 3	LAP	1,021,271	1,043,832	Vazabitide A	NRP (4%)
	Region 4	RRE-containing	1,364,875	1,386,020		
	Region 5	terpene	1,944,236	1,966,098		
	Region 6	T3PKS	2,101,703	2,142,797		
Strain SCS-26^T^	Region 1	T3PKS	1,607,255	1,648,343		
	Region 2	betalactone	2,180,731	2,204,893	Fengycin	NRP (46%)
	Region 3	RiPP-like	2,416,164	2,429,978		
	Region 4	terpene	3,577,539	3,599,407		
Strain SCS-31^T^	Region 1.1	RiPP-like	1,350,910	1,364,811		
	Region 1.2	T3PKS	2,658,890	2,700,095		
	Region 1.3	Terpene	3,533,446	3,555,614		
Strain SCS-37^T^	Region 1	Betalactone	2,088,839	2,113,001	Fengycin	NRP (46%)
	Region 2	T3PKS	2,648,477	2,689,565		
	Region 3	Terpene	3,477,542	3,499,410		
	Region 4	RRE-containing	3,797,387	3,816,695		
	Region 5	Lassopeptide	3,820,431	3,844,323	Paeninodin	RiPP (80%)
Strain SCS-151^T^	Region 5.1	Terpene	661,962	683,833		
	Region 5.2	NI-siderophore	4,301,210	4,315,311		
	Region 5.3	T3PKS	4,428,273	4,469,361		
Strain SCS-153A^T^	Region 1	NI-siderophore	2,076,999	2,090,813		
	Region 2	T3PKS	2,654,238	2,695,311		
Strain SCS-155^T^	Region 1	T3PKS	977,980	1,019,131	Butirosin A/butirosin B	Saccharide (7%)
	Region 2	Betalactone	2,321,808	2,355,473	Fengycin	NRP (46%)
	Region 3	Terpene	2,512,833	2,534,713	Surfactin	NRP:lipopeptide (13%)

**Figure 10:**
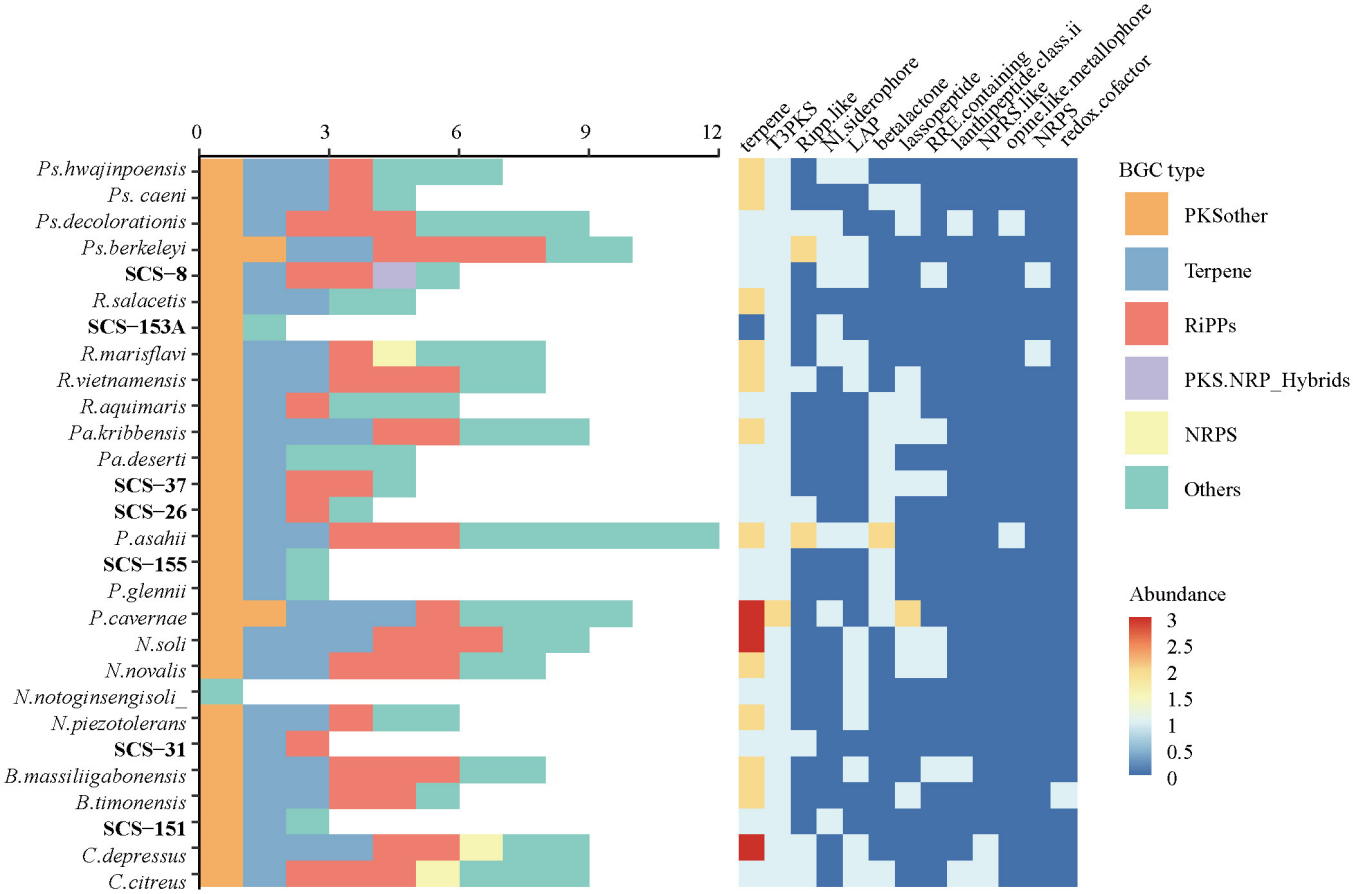
The abundance of different BGC types and subcategories annotated from seven novel strains and their reference species.

Besides, the most predicted BGCs also showed low similarity to known BGCs in the MIBiG database: only one BGC (Region-5 of SCS-31) had a similarity >80%. Among all the analyzed groups, terpenes and other types of polyketide synthase (PKSother) BGC clusters tended to remain with a relatively high abundance (Figure 10). Previous studies have shown that terpenoids can mediate antagonistic interactions between organisms, including maintaining cell membrane stability, antioxidant properties, and the processes of signaling and protection. For instance, isopentenyl adenosine enhances the translation efficiency of modified tRNAs. It reduces their susceptibility to codon context (Boronat and Rodríguez-Concepción, 2014), and hopanoids (as the same function as cholesterol in eukaryotes) can be produced in bacteria to adapt to extreme environments, thereby modulating the permeability of their cell membranes to ensure survival (Vilcheze et al., 1994). Furthermore, 17 BGCs could not be predicted with similar known metabolites, suggesting that these novel strains have the potential to produce unknown metabolites.

**Figure 11:**
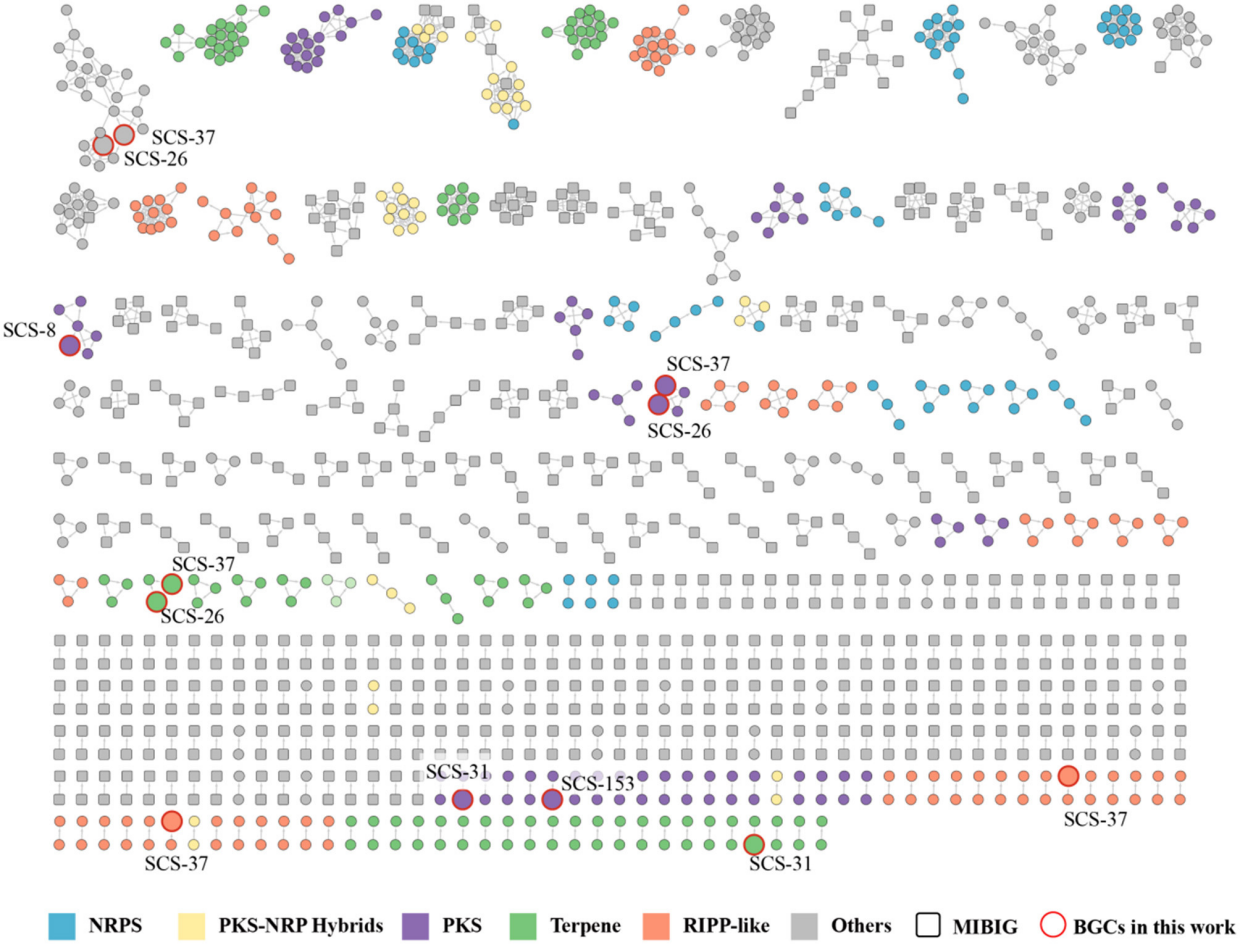
Gene cluster family network of the 1,409 predicted BGCs from 384 *Bacillaceae* family genomes against the MIBiG database, which consists of 182 GCFs (748 singletons not shown). The BGCs predicted from strains in this study were highlighted in red.

To supplement the annotation and calculate the similarity between annotated BGCs, we employed BIG-SCAPE to reveal their evolutionary relationships and functional conservation across the *Bacillaceae* family. A total of 384 complete genomes under the *Bacillaceae* family downloaded from NCBI, which were analyzed by antiSMASH. Based on all the BGCs of seven novel strains and reference genomes above, 1,409 BGCs were produced. The detected BGCs and the known BGCs from the MIBIG database were clustered using the BIG-SCAPE pipeline (with default parameters). The derived gene cluster families (GCFs) represent BGCs with conserved domains and putative functional coherence. The results revealed a high diversity of BGCs in the *Bacillaceae* (Figure 11). The six categories (PKSother, Terpene, PKS-NPRS, RiPPs, NRPS, and “Others”) contain 930 GCFs, including 748 single orphan BGCs and 182 clustered GCF groups, each averaging 3.6 BGCs. The cluster networking analysis may reveal that these strains shared the GCFs with standard metabolic functions or evolutionary relationships. Single orphan BGCs dominated the Terpene and RiPPs categories, and the clustered GCF groups predominated in the PKS-NPRS and NRPS categories. In the PKSother category, the proportions of clustered GCFs and single orphan BGCs were nearly equal (Supplementary Figure S9). This reflects the high diversity of *Bacillaceae* BGCs across the six groups, potentially indicating that the *Bacillaceae* family possesses broad metabolic adaptations.

Among the 26 predicted BGCs of the novel species, only 12 BGCs were able to cluster with existing *Bacillaceae* BGCs, which were distributed among strains SCS-8^T^ (1), SCS-26^T^ (3), SCS-31^T^ (2), SCS-37^T^ (5), and SCS-153A^T^ (1). In contrast, no BGCs from the SCS-151^T^ and SCS-155^T^ strains formed any clusters. Among the clustered groups, strains SCS-26^T^ and SCS-37^T^ exhibited the richest clustering, suggesting that these two strains have highly similar metabolic potentials in BGCs, possibly due to their close similarities and collinearity. The presence of 14 single orphan BGCs indicates that the BGCs contained in these seven novel strains possess a high degree of novelty, and the unexplored biosynthetic pathways may harbor new metabolites.

Of the 26 predicted BGCs in the novel species, only 12 clustered with existing *Bacillaceae* BGCs. These 12 BGCs are distributed as follows: Strains SCS-8^T^ (1), SCS-26^T^ (3), SCS-31^T^ (2), SCS-37^T^ (5), and SCS-153A^T^ (1). Strains SCS-26^T^ and SCS-37^T^ showed the most clustering, indicating highly similar BGC-related metabolic products, due to their close genetic similarities and phylogenetic distance. The 14 single orphan BGCs may suggest the presence of new metabolites from unexplored biosynthetic pathways in these novel strains.

## 4 Discussion

In this study, we explored the microbial diversity in the marine sediments of the South China Sea by amplicon sequencing and cultivation. The results showed the microbial community structure and diversity from corer samples below the seafloor (0–10 cm). The amplicon sequencing data reveal that the family *Bacillaceae* (phylum: *Bacillota*; class *Bacilli*; order: *Caryophanales*) accounts for 0.7% of total ASVs. While almost all cultured microorganisms belong to the *Bacillaceae* family. In the study, cultivated bacterial isolates were less common in amplicon sequencing data, suggesting that *Bacillaceae* species in the original environments may have formed endospores, making DNA extraction from these spore cells challenging in environmental samples (Cupit et al., 2019).

Based on the phylogenomic analysis, genome indices and the characteristic differences in polyphasic taxonomic studies, this study proposes six novel species and one novel genus within the family *Bacillaceae*: *Pseudalkalibacillus nanhaiensis* sp. nov., *Paraperibacillus marinus* sp. nov., *Neobacillus oceani* sp. nov., *Paraperibacillus esterisolvens* sp. nov., *Nanhaiella sioensis* gen. nov., sp. nov., *Rossellomorea sedimentorum* sp. nov., and *Peribacillus sedimenti* sp. nov.; additionally, we describe two new genera, *Paraperibacillus* and *Nanhaiella*, and reclassify three species beyond the novel strains in this study: *P. kribbensis* and *P. deserti* have been reclassified into *Paraperibacillus*, and *B. salacetis* has been reclassified into the genus *Rossellomorea*. To further understand their genomic differences from established strains, the complete genomes of these seven novel strains and reference genomes for functional gene annotation and comparative genomic analysis were analyzed. The results indicated that seven novel strains exhibited reduced genome sizes compared to the reference strains, accompanied by unique metabolic pathways potentially linked to niche adaptation or specialized ecological functions.

Notably, the highest distribution of specific genes was observed in the homologous protein groups of COG-M, COG-G, and COG-Q, which suggests that the candidate novel *Bacillaceae* species exhibit specific functions in cell membrane formation and signaling, carbohydrate metabolism and utilization, and secondary metabolite production. This unique functionality may be associated with their habitat from deep-sea sediment environments.

To help understand the variable in the metabolic pathway of carbohydrate degradation, we combine experimental results in API 50CHB strips and gene annotation results in KEGG and SubtiWiki databases. The utilization of carbon sources from plant cell walls has decreased, while carbon sources related to bacterial and fungal cell walls has increased. This suggests that in the extreme environment of deep-sea sediments, *Bacillaceae* species preferentially utilize bacterial cell walls for carbon metabolism. Further validation might be conducted through experiments designed to assess carbon source utilization.

Based on the predicted BGCs, the seven novel strains have fewer BGCs than the reference strains. This suggests possible genomic streamlining in the deep sea's high-pressure, low-temperature environment. By reducing the energy expenditure on secondary metabolites, these bacteria can focus more on other essential metabolic pathways for survival. Interestingly, the novel *Bacillaceae* strains primarily tend to remain terpenoid compounds as secondary metabolites. Besides, their BGCs show low similarity to known ones in the database and have difficulty forming GCFs in the MIBiG database. This highlights the possible unique metabolic functions of BGCs in these novel strains. Future studies could explore the composition and mechanisms of these compounds through methods such as extraction, isolation, and purification of secondary metabolites.

## 5 Description

### 5.1 Description of *Pseudalkalibacillus nanhaiensis* sp. nov.

*Pseudalkalibacillus nanhaiensis* (nan.hai.en'sis. N.L. masc. adj. nanhaiensis, of or pertaining to Nanhai (South China Sea), where the type strain was isolated).

Cells are Gram-stain positive, aerobic, rod shaped, non-motile, and of 2.0–5.0 μm in length and 0.5–0.8 μm in width. On rich nutrient media, colonies are circular, creamy white, convex, wrinkled, and 10 mm in diameter after incubation at MB for 3 days. Oxidase-positive and catalase-negative. Nitrate is not reduced to nitrite. Growth occurs in medium with 0%−12% NaCl concentration (%, w/v; optimal 1.5−5%), pH 6.0–7.0 (optimal 6.5–8.0), and temperature 25–50°C (optimal 33°C). Positive for hydrolysis of starch, but negative for casein, Tween 40, and Tween 80. The reactions on the API 20NE kit were positive for glucose, arabinose, mannose, mannitol, N-acetyl-glucosamine, maltose, potassium gluconate, adipic acid, malate and phenylacetic acid; weakly positive for hydrolysis (β-glucosidase; esculin) β-galactosidase and negative for glucose, arginine dihydrolase, urease, capric acid and trisodium citrate. API ZYM test strip results indicate that Strain SCS-8^T^ is positive for alkaline phosphatase, esterase, esterase lipase, acid phosphatase, naphthol-AS-BI-phosphohydrolase, and α-glucosidase; negative for lipase, leucine arylamidase, valine arylamidase, cystine arylamidase, trypsin, α-chymotrypsin, α-galactsidase, β-galactosidase, β-glucuronidase, β-glucosidase, N-acetyl-β-glucosaminidase, α-mannosidase, α-fucosidase. Acid production is positive from acetoacetic acid. Other substrates included in the API 50CH strips gave negative results. Using Biolog GENIII plates, the tested SCS-8^T^ strain utilized the following source: glucuronamid—sensitive to carbenicillin, lincomycin, erythromycin, and neomycin but not sensitive to kanamycin, penicillin G, gentamicin, and tetracycline. The sole respiratory quinone was MK-7.

Type strain is SCS-8^T^ (= KCTC 43507^T^ = MCCC 1K08267^T^), which was isolated from the South China Sea. The GenBank/EMBL/DDBJ accession numbers for the 16S rRNA gene sequence and the complete genome sequence are PP882840 and CP143541, respectively.

### 5.2 Description of *Paraperibacillus* gen.nov.

*Paraperibacillus* (Gr. prep. *para*, beside; N.L. masc. n. *Peribacillus*, a genus name; N.L. masc. n. *Paraperibacillus*, resembling the genus *Peribacillus*). Cells are rod shaped, Gram-stain positive, aerobic, motile, spore forming, oxidase positive, and catalase positive. The predominant menaquinone is MK-7. The type species is *Paraperibacillus kribbensis*, which belongs phylogenetically to the family *Bacillaceae*.

### 5.3 Description of *Paraperibacillus deserti* comb. nov.

(des.er'ti. L. gen. neut. n. deserti, of a desert).

Basonym: ***Bacillus deserti*** Zhang et al., 2012.

The description of this species is the same as provided by Zhang et al. (2011) for ***Bacillus deserti***.

Type strain: = CCTCC AB 207173^T^ = KCTC 13246^T^.

### 5.4 Description of *Paraperibacillus kribbensis* comb. nov.

[krib.ben'sis. N.L. masc. adj. kribbensis—arbitrary name formed from the acronym of the Korea Research Institute of Bioscience and Biotechnology (KRIBB), where taxonomic studies on this species were performed].

Basonym: ***Bacillus kribbensis*** Lim et al., 2007.

The description of this species is the same as provided by Lim et al. (2007) for ***B**.* ***kribbensis***.

Type strain: = KCTC 13934^T^ = DSM 17871^T^.

### 5.5 Description of *Paraperibacillus marinus* sp. nov.

*Paraperibacillus marinus* (ma.ri'nus. L. masc. adj. marinus, of or belonging to the sea, marine).

Cells are Gram-stain positive, facultatively anaerobic, motile, rod shaped, and of about 0.6–0.8 μm in width and 2.0–5.0 μm in length. Cells of the strain are catalase- and oxidase-positive. Forms smooth, translucent, whitish with entire margins and < 1 mm in diameter after 3 days incubation at 30°C. Growth occurs with 0−5% (w/v) NaCl, at 25–42°C, and at pH 6.0–8.0. Optimal growth is observed with 0.5–3.0% (w/v) NaCl, at 28–33°C, and at pH 6.5. Nitrate is not reduced to nitrite. Weakly positive for hydrolysis of casein and Tween 20, but negative for hydrolysis of starch, Tween 40, and Tween 80. In API 20E assays, positive for esculin, gelatin, β-galactosidase, glucose, mannitol, n-acetyl-glucosamine, maltose, potassium gluconate, adipic acid, and malate; weakly positive for mannose ans phenylacetic acid; and negative for tryptophane, glucose, arginine dihydrolase, urease, arabinose, capric acid, and trisodium citrate. The reactions on the API ZYM test strips were positive for alkaline phosphatase, esterase, esterase lipase, acid phosphatase, naphthol-AS-BI-phosphohydrolase, β-galactosidase, α-glucosidase; weakly positive for α-chymotrypsin and negative for lipase, leucine arylamidase, valine arylamidase, cystine arylamidase, trypsin, α-galactsidase, β-glucuronidase, β-glucosidase, N-acetyl-β-glucosaminidase, α-mannosidase, and α-fucosidase. Acid is produced from _D−_glucose, _D−_fructose, _D−_mannose, _D−_mannitol, N-acetylglucosamine, esculin, maltose, sucrose, trehalose, inulin and _D−_tagatose. Other substrates included in the API 50CH gave negative results. Using Biolog GENIII plates, the results were positive for the utilization of Dextrin, _D−_maltose, _D−_trehalose, _D−_cellobiose, gentiobiose, sucrose, _D−_turanose, _D−_raffinose, α-_D−_lactose, _D−_melibiose, β-Methyl-_D−_glucoside, _D−_salicin, N-acetyl-_D−_glucosamine N-acetyl-β-_D−_mannosamine, N-acetyl-_D−_galactosamine, α-_D−_glucose, _D−_mannose, _D−_fructose, _D−_galactose, 3-methyl-glucose, _D−_fucose, _L−_rhamnose, inosine, 1 sodium lactate _D−_sorbitol _D−_mannitol glycerol, _D−_glucose-6-PO4, _D−_fructose-6-PO4 gelatin, glycyl-_L−_proline, _L−_alanine, _L−_arginine, _L−_pyroglutamic acid, _L−_serine, pectin, _L−_galactonic acid lactone, _D−_gluconic acid, methyl pyruvate, α-keto-glutaric acid, _L−_malic acid, bromo-succinic acid, nalidixic acid, potassium tellurite, sodium butyrate, and acetic acid. Weakly sensitive to kanamycin and lincomycin but not sensitive to carbenicillin, penicillin G, gentamicin, erythromycin, neomycin, and tetracycline. The sole respiratory quinone was MK-7.

Type strain is SCS-26^T^ (= KCTC 43508^T^ = MCCC 1K08268^T^), which was isolated from the South China Sea. The GenBank/EMBL/DDBJ accession numbers for the 16S rRNA gene sequence and complete genome sequence are PQ606470 and CP143542, respectively.

### 5.6 Description of *Paraperibacillus esterisolvens* sp. nov.

*Paraperibacillus esterisolvens* (es.te.ri.sol'vens. N.L. neut. n. ester, ester; L. pres. part. solvens, degrading; N.L. masc. part. adj. esterisolvens, ester-degrading).

Cells of the strains were gram-stain positive, facultatively anaerobic, rod shaped, oxidase and catalase positive, 1–5 μm in length and 0.5–0.8 μm in width, and capable of forming endospores. On Columbia agar, these strain colonies appear circular with creamy white, convex, smooth, and have a diameter of about 2 mm. Growth occurs in medium with 0–5% (w/v) NaCl concentration (optimum: 0.5–2%, w/v), 25–42°C (optimum: 30–33°C), and pH 6.0–10.0 (optimum: pH 5.0–7.0). Positive for hydrolysis of Tween 20, Tween 40, and Tween 80, weakly positive for casein, but negative for hydrolysis of starch. Nitrate is not reduced to nitrite. In the API 20NE tests, the results were positive for the activities of esculin, gelatin, β-galactosidase, glucose, mannitol, N-acetyl-glucosamine, maltose, potassium gluconate, malate, mannose; weakly positive for adipic acid, trisodium citrate, phenylacetic acid and negative for tryptophane, glucose, arginine dihydrolase, urease, arabinose and capric acid. API ZYM test strip results indicate that Strain SCS-37^T^ is positive for alkaline phosphatase, esterase lipase, lipase, leucine arylamidase, valine arylamidase, cystine arylamidase; weakly positive for esterase, trypsin, α-chymotrypsin and negative for α-galactsidase, β-glucuronidase, β-glucosidase, N-acetyl-β-glucosaminidase, α-mannosidase, and α-fucosidase. Acid is produced from Glycerol, Galactose, _D−_glucose, _D−_fructose, _D−_mannitol, Alpha-methyl-_D−_glucoside, N-acetylglucosamine, amygdalin, esculin, salicin, cellobiose, maltose, sucrose, trehalose, starch, β-Gentiobiose, _D−_turanose. Other substrates included in the API 50CHB system gave negative results. Using Biolog GENIII plates, the results were positive for the utilization of dextrin, _D−_maltose, _D−_trehalose, _D−_cellobiose, gentiobiose, sucrose, _D−_turanose, β-methyl-_D−_glucoside, _D−_salicin, N-acetyl-_D−_glucosamine N-acetyl-β-_D−_mannosamine, N-Acetyl-_D−_galactosamine, α-_D−_glucose, _D−_mannose, _D−_galactose, inosine, 1 sodium lactate, _D−_sorbitol, _D−_mannitol, glycerol, gelatin, _L−_arginine, _L−_pyroglutamic acid, _L−_serine, pectin, _D−_gluconic acid, methyl pyruvate, _D−_aactic acid methyl ester, _L−_lactic acid, citric acid, α-keto-glutaric acid, _L−_malic acid, nalidixic acid, β-hydroxy-D, lbutyric acid, acetoacetic acid, acetic acid and sodium butyrate. Sensitive to carbenicillin, kanamycin, lincomycin, and penicillin G but not sensitive to erythromycin, gentamicin, neomycin, and tetracycline. The sole respiratory quinone was MK-7.

Type strain is SCS-37^T^ (= KCTC 43510^T^ = MCCC 1K08269^T^), which was isolated from the South China Sea. The GenBank/EMBL/DDBJ accession numbers for the 16S rRNA gene sequence and draft genome sequence are PQ606472 and CP143545, respectively.

### 5.7 Description of *Neobacillus oceani* sp. nov.

*Neobacillus oceani* (o.ce.a'ni. L. gen. masc. n. oceani, of the ocean).

Cells are facultatively anaerobic, Gram-stain positive, spore forming, motile rods that are 0.3–0.5 μm in width and 1.0–2.0 μm in length. Colonies on medium are whitish, translucent, with a tiny rise and ~ < 1 mm in diameter after 3 days of growth at 30°C. Oxidase and catalase are produced. Nitrate can be reduced to nitrite. Growth occurs at 25–50°C and in the presence of 0–5% (w/v) NaCl, with the optimum growth at 33°C and with 2% (w/v) NaCl. The optimum pH for growth is pH 6.0–8.0; growth does not occur below pH 6.0 or above pH 8.5. Decomposes casein, Tween 60, and Tween 80, does not hydrolyze starch, Tween 20, and Tween 40. In the API 20 ZYM tests, the results were positive for alkaline phosphatase, esterase, esterase lipase, leucine arylamidase, valine arylamidase, cystine arylamidase, naphthol-AS-BI-phosphohydrolase, α-glucosidase; weakly positive for trypsin, α-chymotrypsin, acid phosphatase, β-glucosidase, and negative for lipase, α-galactsidase, β-galactosidase, β-glucuronidase, N-acetyl-β-glucosaminidase, α-mannosidase, and α-fucosidase. According to API 20NE, esculin, β-galactosidase, mannitol, N-acetyl-glucosamine, potassium gluconate, adipic acid, phenylacetic acid are present, but glucose, arginine dihydrolase, urease, gelatin, arabinose, maltose, and trisodium citrate are absent. Acid is produced from Erythritol, _D−_Glucose, _D−_Fructose, Mannitol, α-methyl-_D−_glucoside, N-acetylglucosamine, amygdalin, esculin, salicin, cellobiose, maltose, sucrose, trehalose, starch, and β-gentiobiose. Other substrates included in the API 50CHB gave negative results. GEN III tests showed positive results for acetoacetic acid, acetic acid, nalidixic acid, aztreonam, and sodium butyrate. Not sensitive to carbenicillin, kanamycin, lincomycin, penicillin G, erythromycin, gentamicin, neomycin, and tetracycline. The sole respiratory quinone was MK-7.

Type strain is SCS-31^T^ (= KCTC 43509^T^ = MCCC 1K08361^T^), which was isolated from the South China Sea. The GenBank/EMBL/DDBJ accession numbers for the 16S rRNA gene sequence and complete genome sequence are PQ606471 and CP143543, respectively.

### 5.8 Description of *Nanhaiella* gen.nov.

*Nanhaiella* (Nan.hai.el.la. N.L. dim. fem. n. *Nanhaiella*, named after Nanhai, the Chinese name for the South China Sea, referring to the region where the bacterium was isolated). Gram-positive, rod shaped, facultatively anaerobic, spore forming, and oxidase and catalase positive. The predominant menaquinone is MK-7. The type species is *Nanhaiella sioensis*, which belongs phylogenetically to the family *Bacillaceae*.

### 5.9 Description of *Nanhaiella sioensis* sp. nov.

*Nanhaiella sioensis* (sio.en'sis. N.L. masc. adj. sioensis, arbitrary name derived from the acronym SIO for Second Institute of Oceanography, Ministry of Natural Resources, where the taxonomic studies on this novel species were conducted).

Cells are Gram-positive, rod shaped, facultatively anaerobic, spore forming, oxidase and catalase positive, 1–4 μm in length and 0.5–0.7 μm in width. Growth occurs in medium with 0–5% (w/v) NaCl (optimum 0.5–2.0%), 25–42°C (optimum 30–33°C), and pH 6.0–9.0 (optimum pH 8.0). Negative for hydrolysis of Tween 20, Tween 40, Tween 80, casein, and starch. In the API 20NE tests, the results were positive for glucose fermentation, β-glucosidase hydrolysis, glucose assimilation, N-acetyl-glucosamine, maltose, potassium gluconate, adipic acid, malic acid, and phenylacetic acid, weakly positive for β-galactosidase, arabinose, mannose, and mannitol. In the API 20 ZYM tests, the results were positive for alkaline phosphatase, esterase (C4), acid phosphatase, naphthol-AS-BI-phosphohydrolase, and weakly positive for α-chymotrypsin. In the API 50CHB system, acid production occurs using esculin ferric citrate, but no acid is produced in other substrates in the API 50CHB system. Using Biolog GENIII plates, the results were positive for dextrin, _D_-maltose, _D_-trehalose, gentiobiose, _D_-turanose, α-_D_-lactose, _D_-melibiose, _D_-salicin, n-acetyl-_D_-glucosamine, α-_D_-glucose, _D_-galactose, 3-methyl-glucose, _D_-fucose, _L_-fucose, _L_-rhamnose, inosine, 1% sodium lactate, myo-inositol, glycerol, _D_-glucose-6-PO4, _D_-fructose-6-PO4, gelatin, glycyl-_L_-proline, _L_-alanine, _L_-arginine, _L_-glutamic acid, _L_-histidine, _L_-pyroglutamic acid, pectin, _D_-galacturonic acid, _L_-galactonic acid lactone, _D_-gluconic acid, _D_-glucuronic acid, glucuronamid e, quinic acid, tetrazolium violet, tetrazolium blue, methyl pyruvate, _D_-lactic acid methyl ester, L-lactic acid, nalidixic acid, lithium chloride, potassium tellurite, tween 40, α-hydroxy-butyric acid, α-keto-butyric acid, acetoacetic acid, acetic acid, and sodium butyrate. Using Biolog GENIII plates, the results were weakly positive for the utilization of glucuronamid, tetrazolium violet, and tetrazolium blue. Sensitive to carbenicillin, gentamicin, neomycin, and tetracycline but not sensitive to kanamycin, lincomycin, and penicillin G. MK-7 is the predominant respiratory quinone.

Type strain is SCS-151^T^ (= MCCC 1K08270^T^), which was isolated from the South China Sea. The GenBank/EMBL/DDBJ accession numbers for the 16S rRNA gene sequence and draft genome sequence are PQ606473 and JBKAEG000000000, respectively.

### 5.10 Description of *Rossellomorea salacetis* comb. nov.

(sal.a.ce'tis. L. masc. n. sal, salt; N.L. masc. n. Acetes, a shrimp genus; N.L. gen. masc. n. salacetis, of salt shrimp).

Basonym: ***Bacillus salacetis*** Daroonpunt et al., 2019.

The description of this species is the same as provided by Daroonpunt et al. (2019) for ***Bacillus salacetis***.

Type strain: = JCM 33205^T^ = KCTC 43014^T^ = TISTR 2596^T^.

### 5.11 Description of *Rossellomorea sedimentorum* sp. nov.

*Rossellomorea sedimentorum* (se.di.men.to'rum. L. gen. neut. pl. n. sedimentorum, of settlings, subsidences, here intended to mean of sediments, referring to the source of isolation).

Cells are Gram-positive, rod shaped, facultatively anaerobic, spore forming, oxidase positive, and catalase negative, 1.0–3.5 μm in length and 0.5–0.7 μm in width. Growth occurs in medium with 0−10% (w/v) NaCl (optimum 2.0−3.0%), 25–44°C (optimum 20–37°C), and pH 6.0–9.0 (optimum pH 6.0–7.0). Positive for hydrolysis of starch, weakly positive for Tween 80, but negative for hydrolysis of Tween 40 and Tween 60. In API 20NE test, the results were positive for glucose assimilation, arabinose, mannose, mannitol, N-acetyl-glucosamine, maltose, capric acid and malic acid, weakly positive for adipic acid, but indole production, glucose fermentation, arginine dihydrolase, urease, esculin, gelatin and β-galactosidase and potassium gluconate and trisodium citrate and phenylacetic acid were negative. Nitrate is not reduced to nitrite. In the API ZYM test strip kit, the results were positive for α-chymotrypsin, naphthol-AS-BI-phosphohydrolase, and α-glucosidase, weakly positive for alkaline phosphatase, esterase (C4), esterase lipase (C8), and leucine arylamidase. In API 50CH systems, acid production occurs using galactose, _D−_glucose, _D−_fructose, mannitol, Alpha-methyl-_D−_glucoside, N-acetylglucosamine, amygdalin, esculin ferric citrate, salicin, cellobiose, maltose, sucrose, trehalose, starch, and β-gentiobiose, but no acid is produced in other substrates in the API 50CHB system. Using Biolog GENIII plates, the results were positive for _D−_serine, _D−_fructose-6-PO4, glucuronamid, tetrazolium violet, tetrazolium blue, acetoacetic acid, propionic acid, acetic acid. Not sensitive to carbenicillin, kanamycin, lincomycin, penicillin G, erythromycin, gentamicin, neomycin, and tetracycline. MK-7 is the predominant respiratory quinone.

Type strain is SCS-153A^T^ (= MCCC 1K08314^T^), which was isolated from the South China Sea. The GenBank/EMBL/DDBJ accession numbers for the 16S rRNA gene sequence and complete genome sequence are PQ606474 and CP143546, respectively.

### 5.12 Description of *Peribacillus sedimenti* sp. nov.

*Peribacillus sedimenti* (se.di.men'ti. L. gen. neut. n. sedimenti, of sediment).

Cells are Gram-positive, rod shaped, facultatively anaerobic, spore forming, oxidase and catalase positive, 1–4 μm in length, and 0.5–0.7 μm in width. Growth occurs in medium with 0%−3% NaCl (optimum 1.0%), 28–45°C (optimum 33°C), and pH 6.0 to 7.5 (optimum pH 6.0–7.0). Positive for hydrolysis of Tween 20, but negative for hydrolysis of Tween 40 and Tween 80. Biochemical characteristics were tested using the API ZYM test strip kit, API 20NE kit, and API 50CH strips (bioMérieux) following the manufacturer's instructions. In the API 20NE test, the results were positive for urease, gelatin, glucose assimilation, arabinose, mannose, mannitol, N-Acetyl-glucosamine, maltose, potassium gluconate, capric acid, adipic acid, malic acid, trisodium citrate, and phenylacetic acid. In the API ZYM test strip kit, the results were positive for alkaline phosphatase, leucine arylamidase, valine arylamidase, cystine arylamidase, trypsin, α-chymotrypsin, acid phosphatase, and naphthol-AS-BI-phosphohydrolase, weakly positive for esterase (C4) and esterase lipase (C8). In the API 50CHB system, acid production occurs in _D_-glucose, _D_-fructose, esculin, salicin, cellobiose, maltose, sucrose, trehalose, and β-gentiobiose, but no acid is produced in other substrates in the API 50CHB system. Sensitive to carbenicillin, kanamycin, lincomycin, penicillin G, erythromycin, gentamicin, neomycin, and tetracycline. Using Biolog GENIII plates, the results were positive. The sole respiratory quinone was MK-7.

Type strain is SCS-155^T^ (= MCCC 1K08271^T^), which was isolated from the South China Sea. The GenBank/EMBL/DDBJ accession numbers for the 16S rRNA gene sequence and complete genome sequence are PQ606475 and CP143547, respectively.

## Data Availability

The data presented in this study are deposited in the GenBank repository, accession numbers: CP143541-CP143547, JBKAEG000000000.
