# Supplementary material for: Insights into the bacterial fraction of sediments from the South China Sea: physiological, chemotaxonomic, and genomic characterization of seven novel members of the Bacillaceae family
Source: Front Microbiol. 2025 Jul 4;16:1553679. doi: 10.3389/fmicb.2025.1553679 (PMC12272613; doi:10.3389/fmicb.2025.1553679)
Supplement: Supplementary file 1 [file Supplementary_file_1.docx]

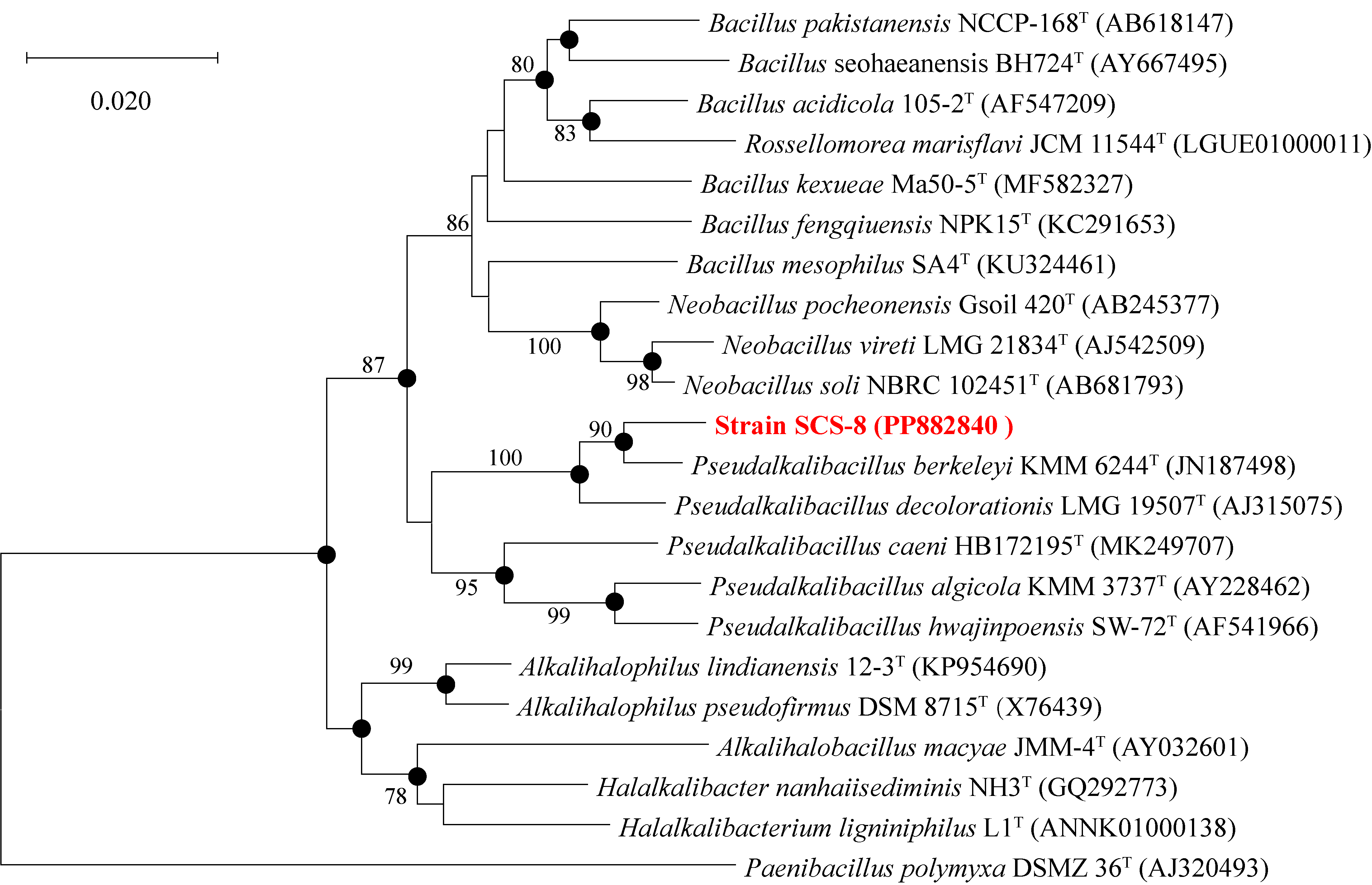


**Figure S1-A. Phylogenetic placement of Strain SCS-8 (red bold type) to closely related type strains (16S rRNA gene accessions in parentheses) based on nearly full-length 16S rRNA gene sequences.** *Paenibacillus polymyxa* DSMZ 36^T^(AJ320493) was used as an outgroup. Filled circles indicate nodes that were also recovered in Maximum-likelihood and Neighbour-joining trees based on the same sequences. Bootstrap values (expressed as percentages of 2000 replications) are shown at branch points. Bar, 0.02 nucleotide substitution per position.


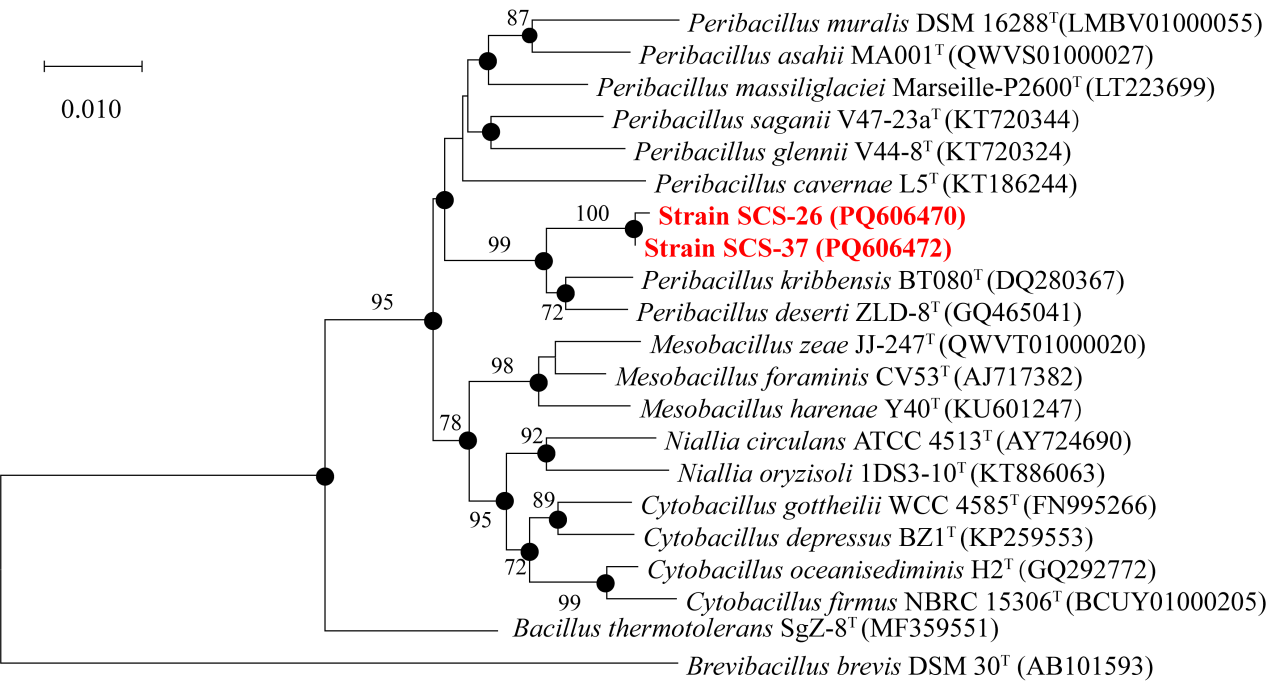


**Figure S1-B. Phylogenetic placement of Strain SCS-26 and SCS-37 (red bold type) to closely related type strains (16S rRNA gene accessions in parentheses) based on nearly full-length 16S rRNA gene sequences.** *Brevibacillus brevis* DSM 30^T^(AB101593) was used as an outgroup. Filled circles indicate nodes that were also recovered in Maximum-likelihood and Neighbour-joining trees based on the same sequences. Bootstrap values (expressed as percentages of 2000 replications) are shown at branch points. Bar, 0.01 nucleotide substitution per position.


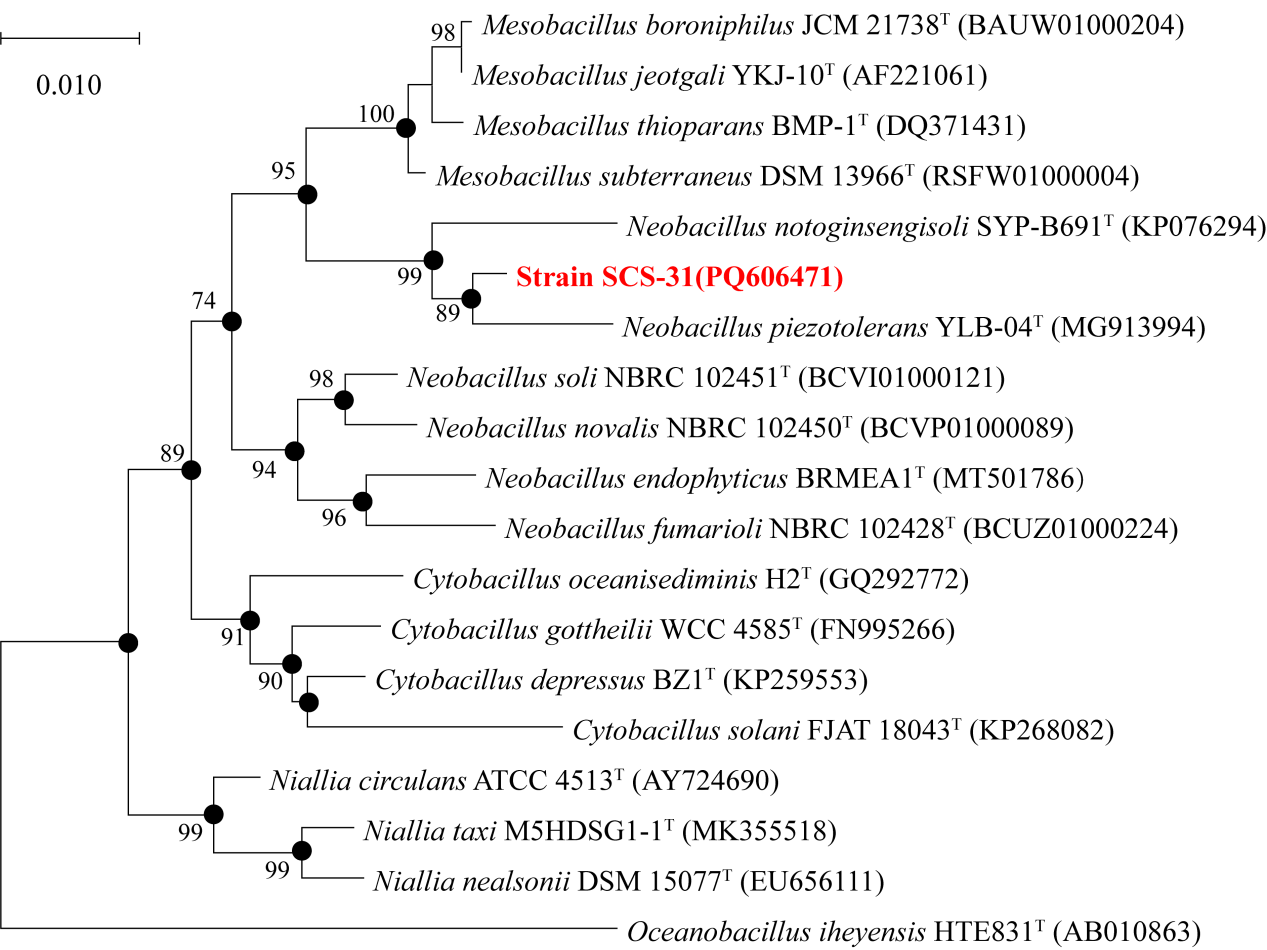


**Figure S1-C. Phylogenetic placement of strain SCS-31 (red bold type) to closely related type strains (16S rRNA gene accessions in parentheses) based on nearly full-length 16S rRNA gene sequences.** *Oceanobacillus iheyeniss* HTE831^T^(AB010863) was used as an outgroup. Filled circles indicate nodes that were also recovered in Maximum-likelihood and Neighbour-joining trees based on the same sequences. Bootstrap values (expressed as percentages of 2000 replications) are shown at branch points. Bar, 0.01 nucleotide substitution per position.


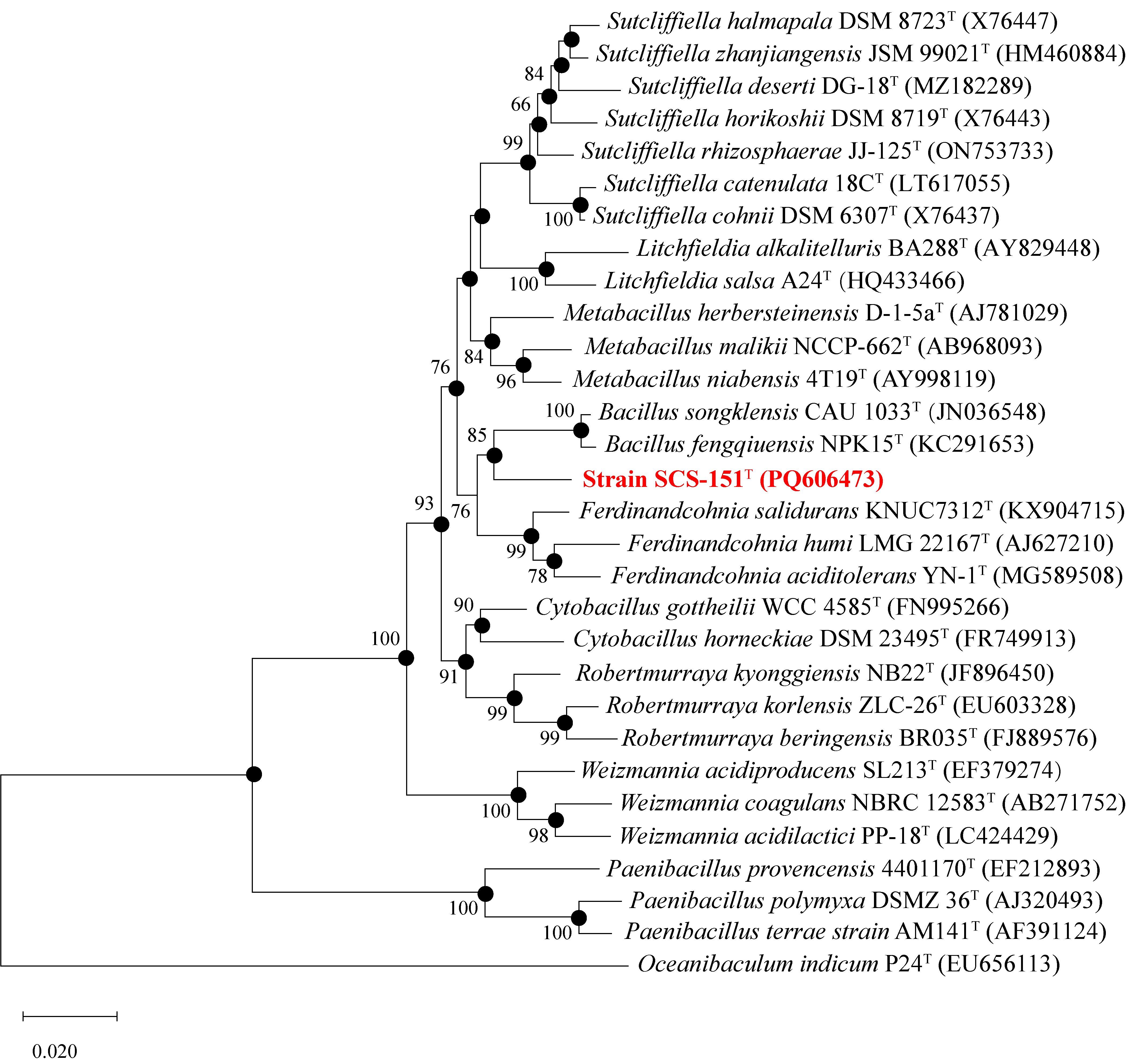


**Figure S1-D. Phylogenetic placement of strain SCS-151 (red bold type) to closely related type strains (16S rRNA gene accessions in parentheses) based on nearly full-length 16S rRNA gene sequences.** *Oceanibaulum indicum* P24^T^(EU656113) was used as an outgroup. Filled circles indicate nodes that were also recovered in Maximum-likelihood and Neighbour-joining trees based on the same sequences. Bootstrap values (expressed as percentages of 2000 replications) are shown at branch points. Bar, 0.02 nucleotide substitution per position.


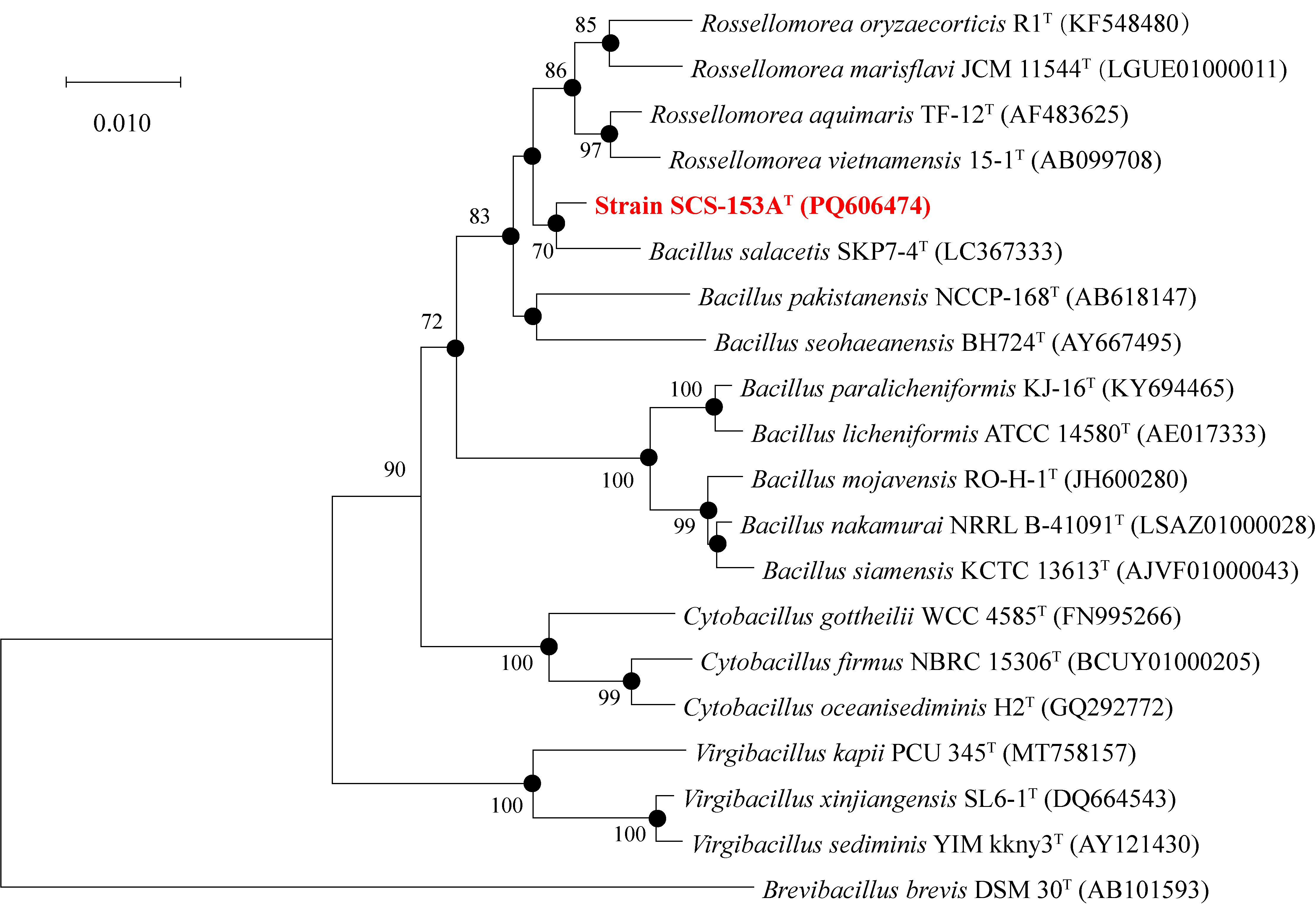


**Figure S1-E. Phylogenetic placement of strain SCS-153A (red bold type) to closely related type strains (16S rRNA gene accessions in parentheses) based on nearly full-length 16S rRNA gene sequences.** *Brevibacillus brevis* DSM 30^T^(AB101593) was used as an outgroup. Filled circles indicate nodes that were also recovered in Maximum-likelihood and Neighbour-joining trees based on the same sequences. Bootstrap values (expressed as percentages of 2000 replications) are shown at branch points. Bar, 0.01 nucleotide substitution per position.


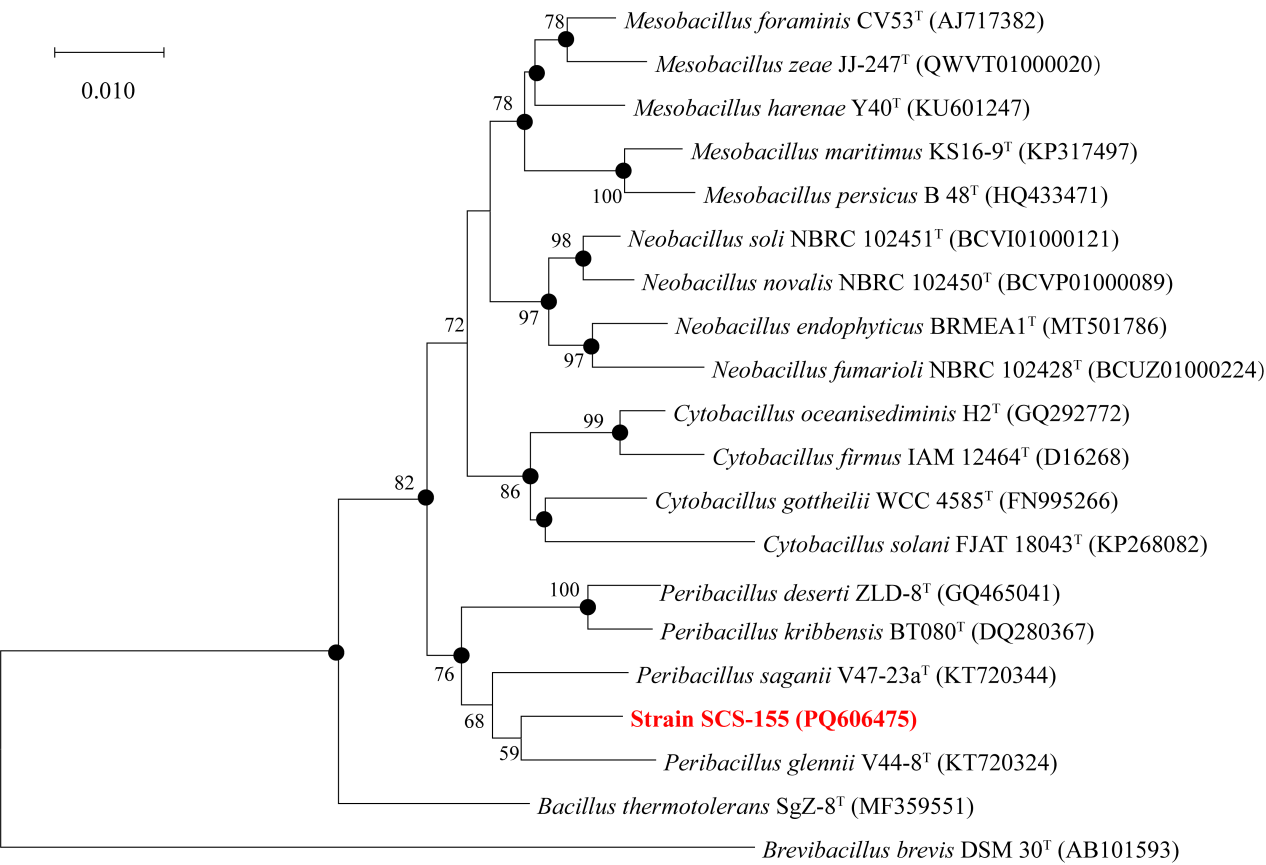


**Figure S1-F. Phylogenetic placement of strain SCS-155 (red bold type) to closely related type strains (16S rRNA gene accessions in parentheses) based on nearly full-length 16S rRNA gene sequences.** *Brevibacillus brevis* DSM 30^T^(AB101593) was used as an outgroup. Filled circles indicate nodes that were also recovered in Maximum-likelihood and Neighbour-joining trees based on the same sequences. Bootstrap values (expressed as percentages of 2000 replications) are shown at branch points. Bar, 0.01 nucleotide substitution per position.


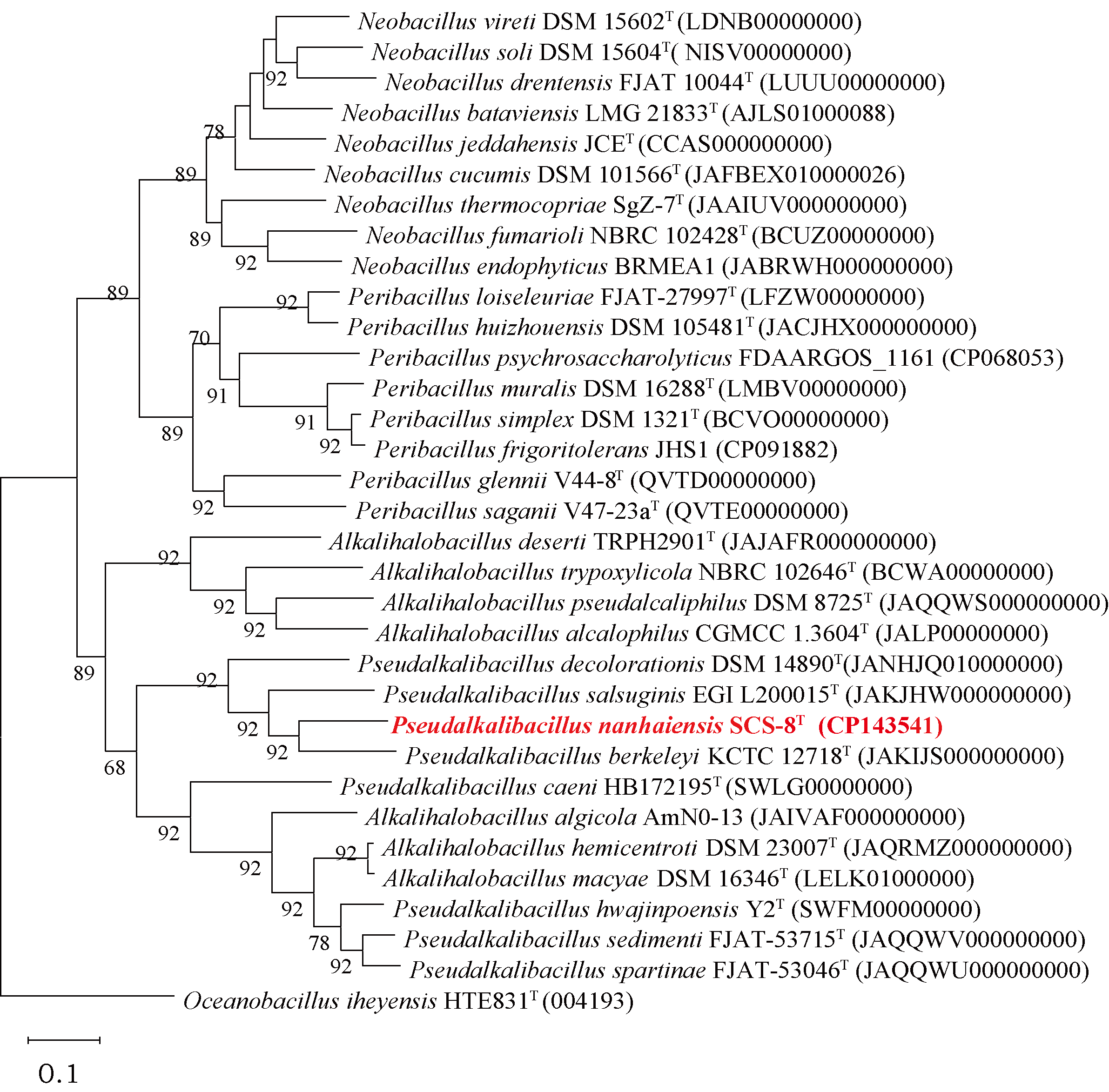


**Figure S2-A. Phylogenomic tree of strain SCS-8 (red bold type) constructed using the 92 bacterial core gene sequences.** The 92 gene sequences were extracted using the Up-to-date bacterial core gene (UBCG) tool and subjected to FastTree to reconstruct a maximum likelihood tree with default parameters. Bar, 0.1 nucleotide substitution per position. *Oceanobacillus iheyeniss* HTE831^T^ was used as an outgroup.


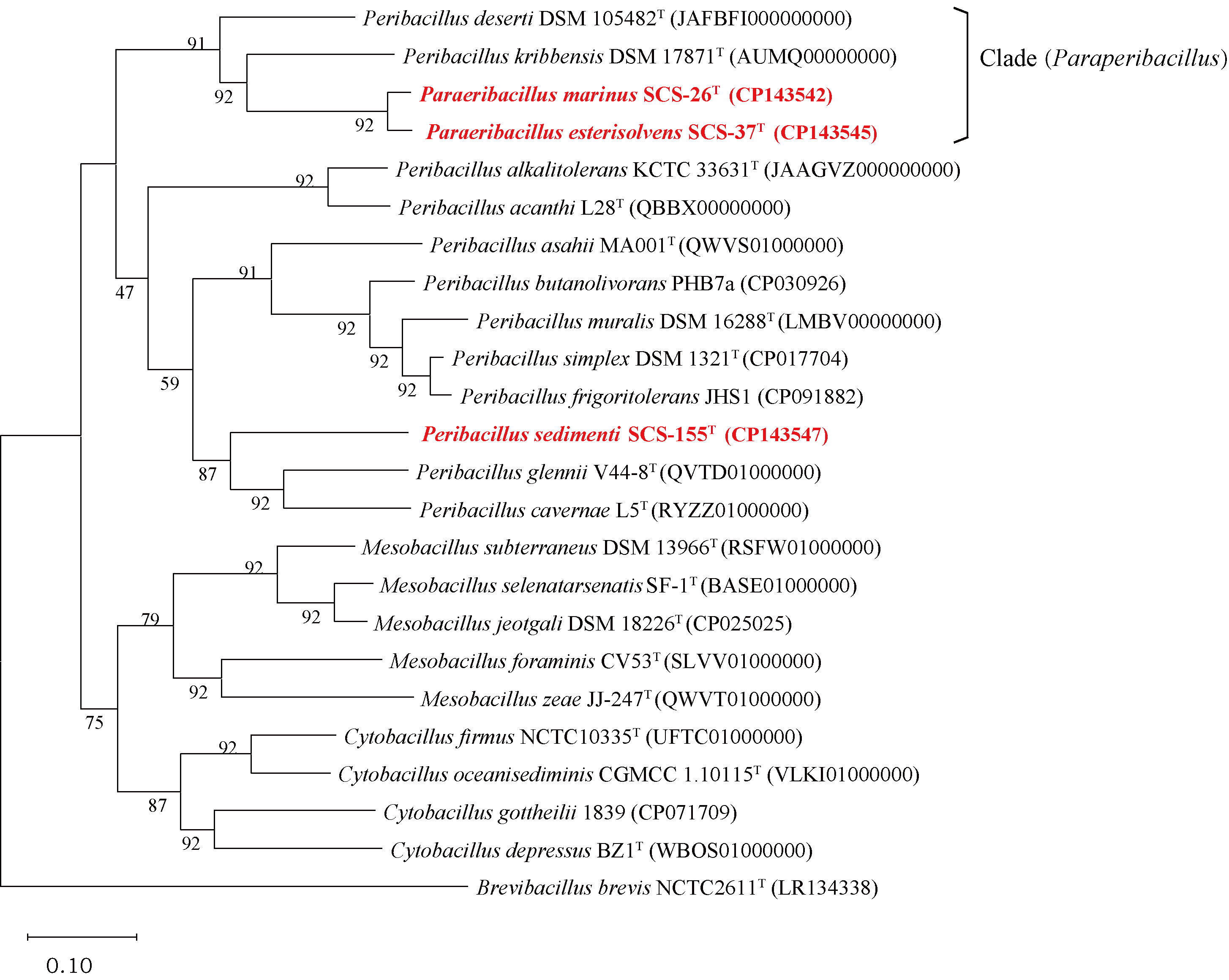


**Figure S2-B. Phylogenomic tree of Strain SCS-26, SCS-37 and SCS-155 (red bold type) constructed using the 92 bacterial core gene sequences.** The 92 gene sequences were extracted using the Up-to-date bacterial core gene (UBCG) tool and subjected to FastTree to reconstruct a maximum likelihood tree with default parameters. Bar, 0.1 nucleotide substitution per position. *Brevibacillus brevis* NCTC2611^T^ was used as an outgroup.

**
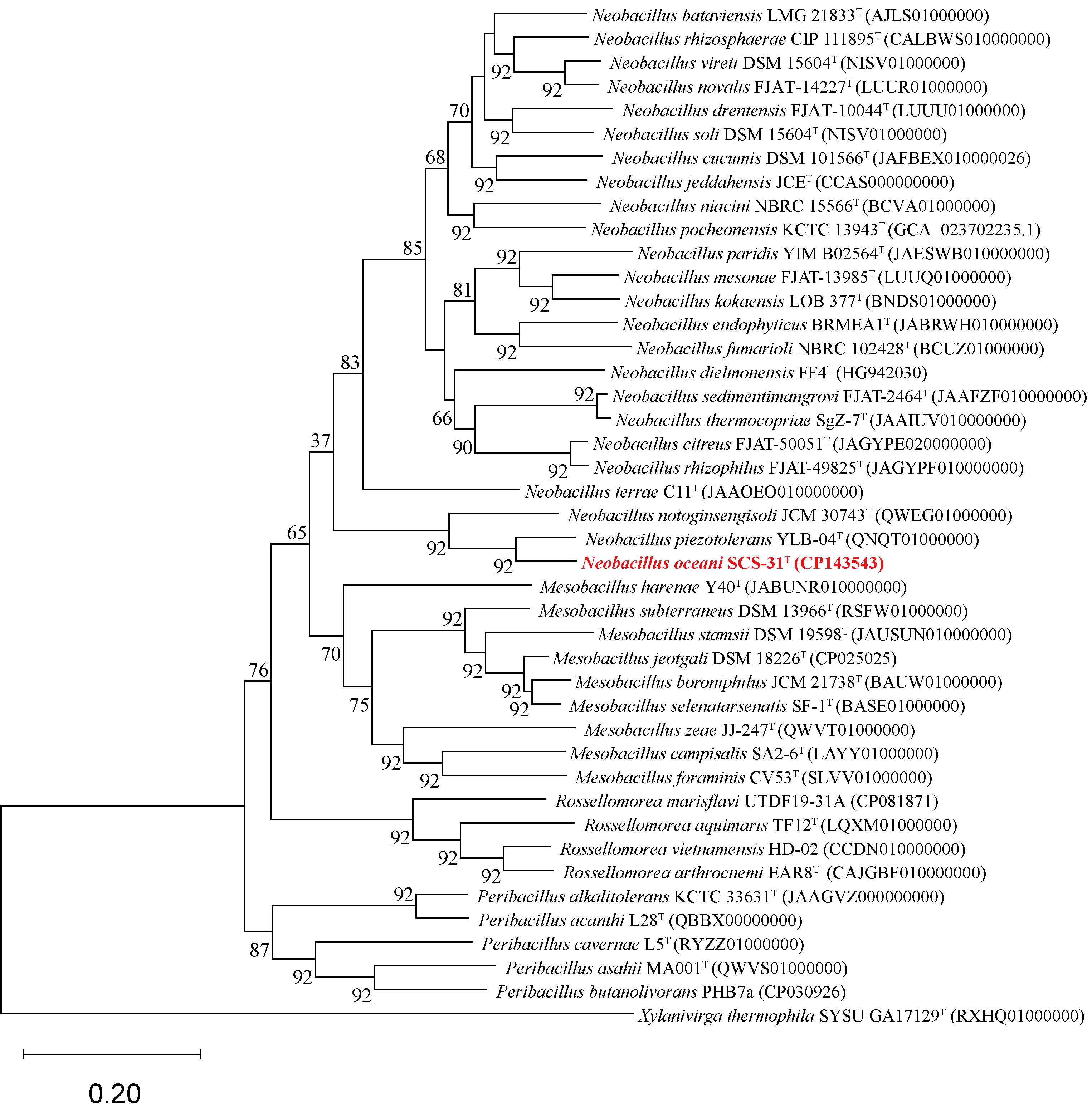
**

**Figure S2-C. Phylogenomic tree of strain SCS-31 (red bold type) constructed using the 92 bacterial core gene sequences.** The 92 gene sequences were extracted using the Up-to-date bacterial core gene (UBCG) tool and subjected to FastTree to reconstruct a maximum likelihood tree with default parameters. Bar, 0.1 nucleotide substitution per position. *Oceanobacillus iheyeniss* HTE831^T^ was used as an outgroup.


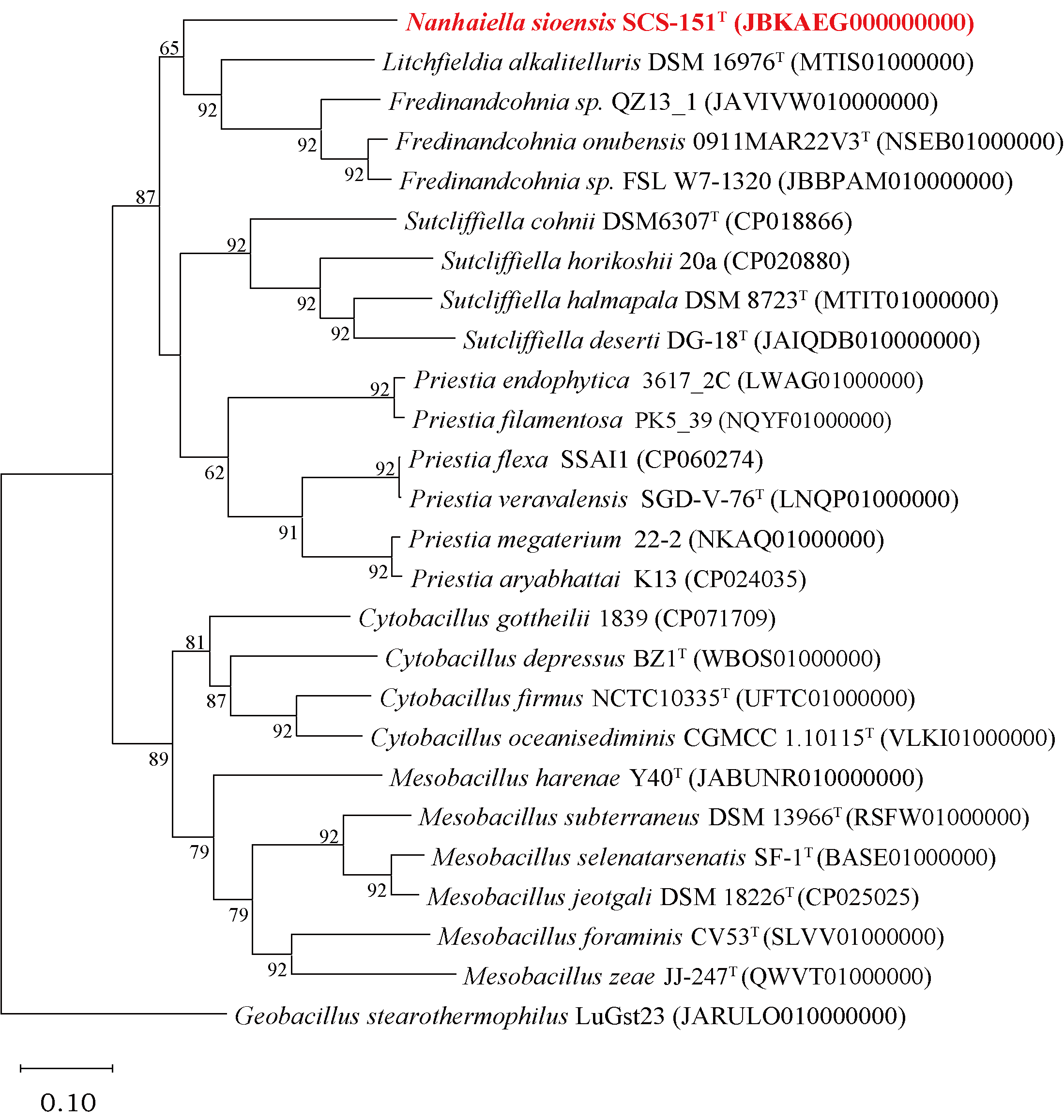


**Figure S2-D. Phylogenomic tree of strain SCS-151 (red bold type) constructed using the 92 bacterial core gene sequences.** The 92 gene sequences were extracted using the Up-to-date bacterial core gene (UBCG) tool and subjected to FastTree to reconstruct a maximum likelihood tree with default parameters. Bar, 0.1 nucleotide substitution per position. *Geobacillus stearothermophilus* LuGst23^T^ was used as an outgroup.


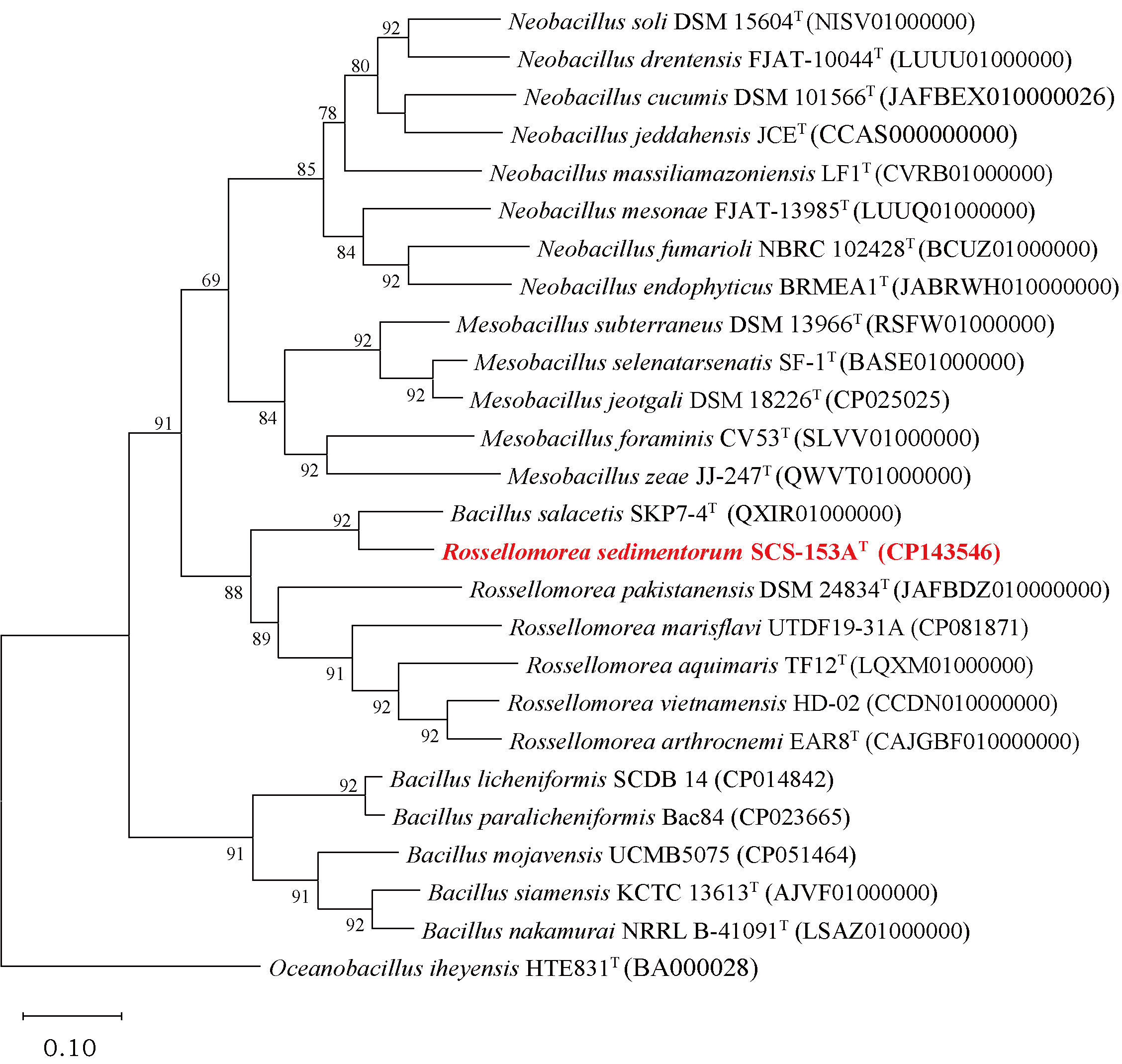


**Figure S2-E. Phylogenomic tree of strain SCS-153A (red bold type) constructed using the 92 bacterial core gene sequences.** The 92 gene sequences were extracted using the Up-to-date bacterial core gene (UBCG) tool and subjected to FastTree to reconstruct a maximum likelihood tree with default parameters. Bar, 0.1 nucleotide substitution per position. *Oceanobacillus iheyeniss* HTE831^T^ was used as an outgroup.


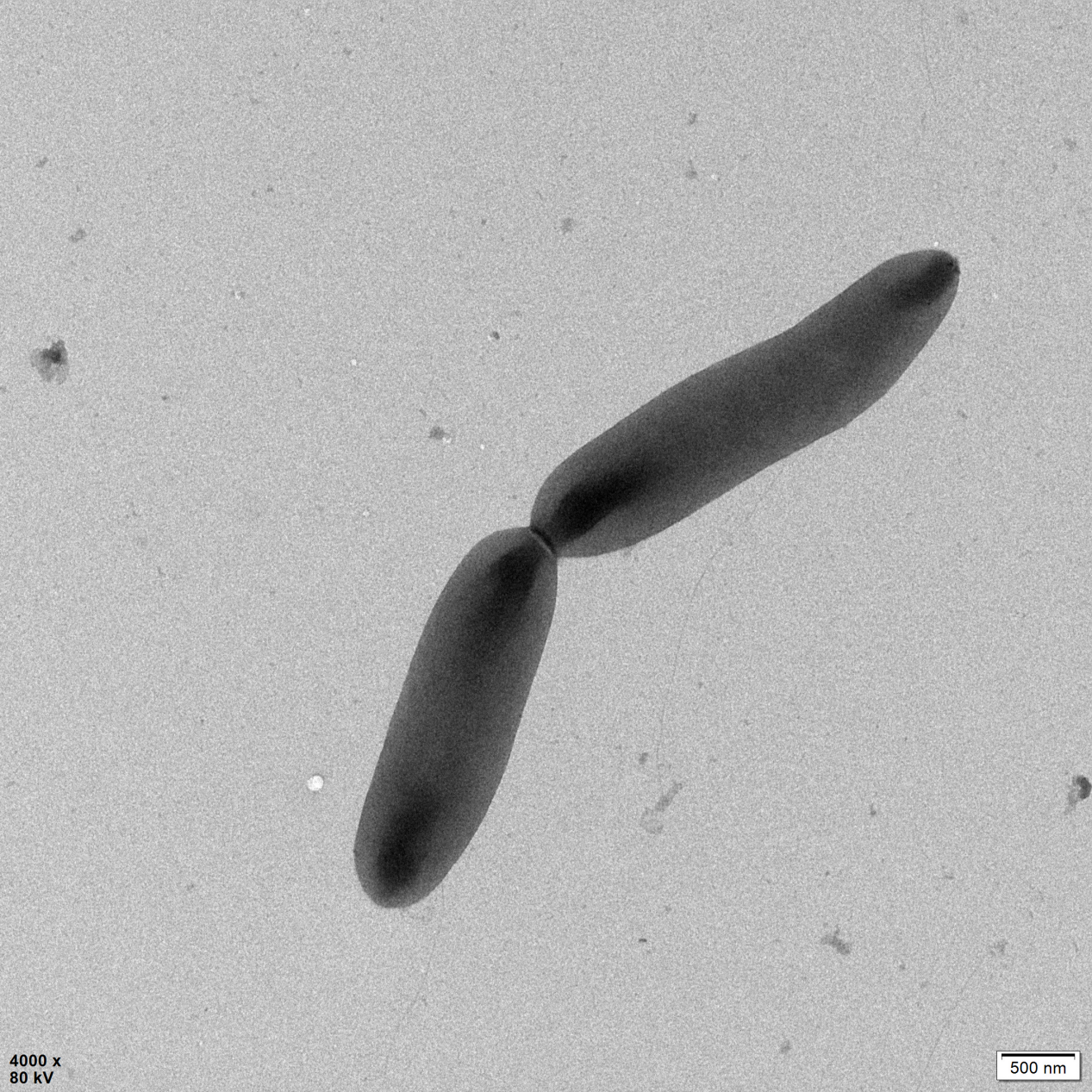


**Figure S3-A.** Transmission Electron micrograph of the Strain SCS-8 culture growth in MB at 30 °C for 2 days depicts rod-shaped bacteria cells. Bar length, 500 nm.


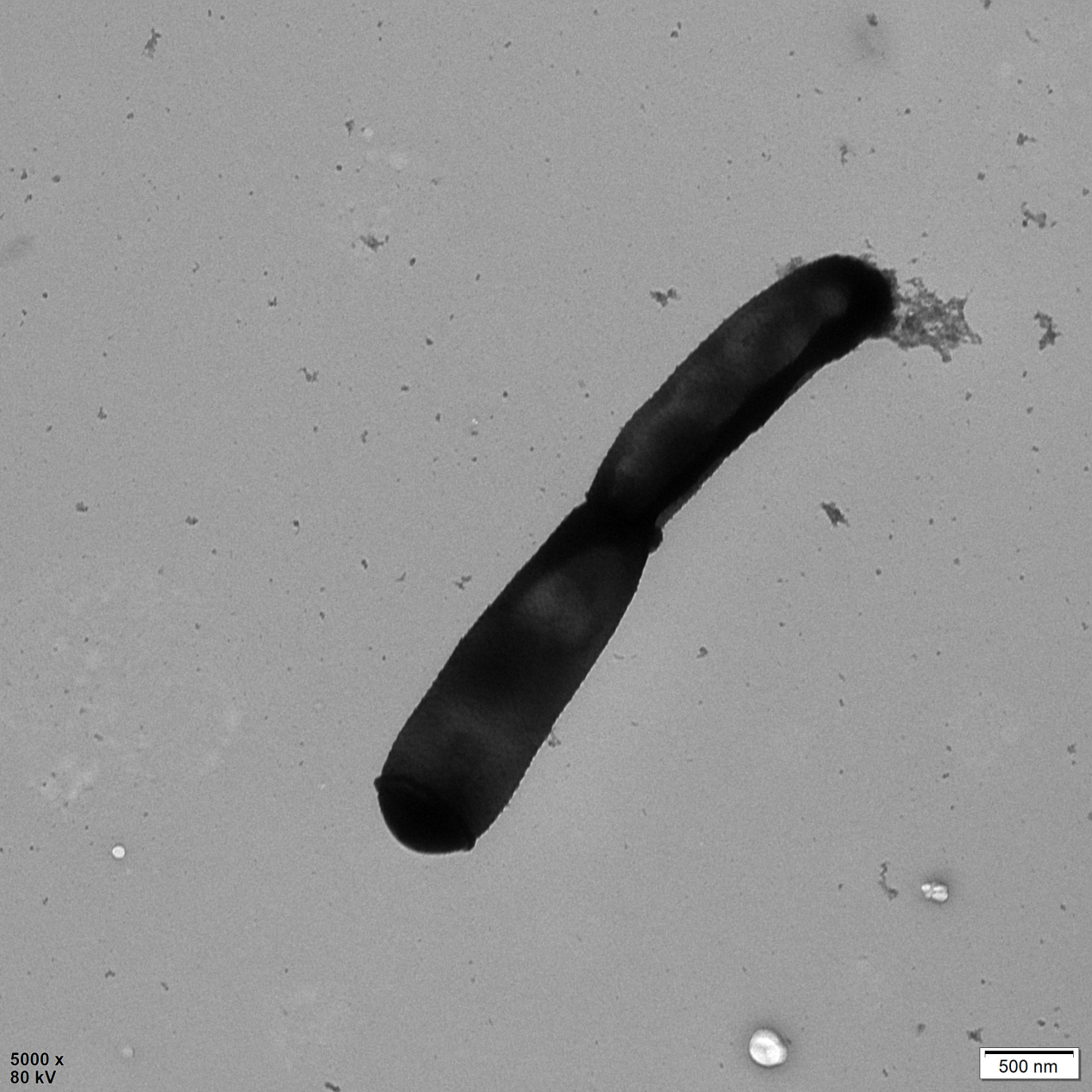


**Figure S3-B.** Transmission Electron micrograph of the Strain SCS-26 culture growth in MB at 30°C for 2 days depicts rod-shaped bacteria cells. Bar length, 500 nm.


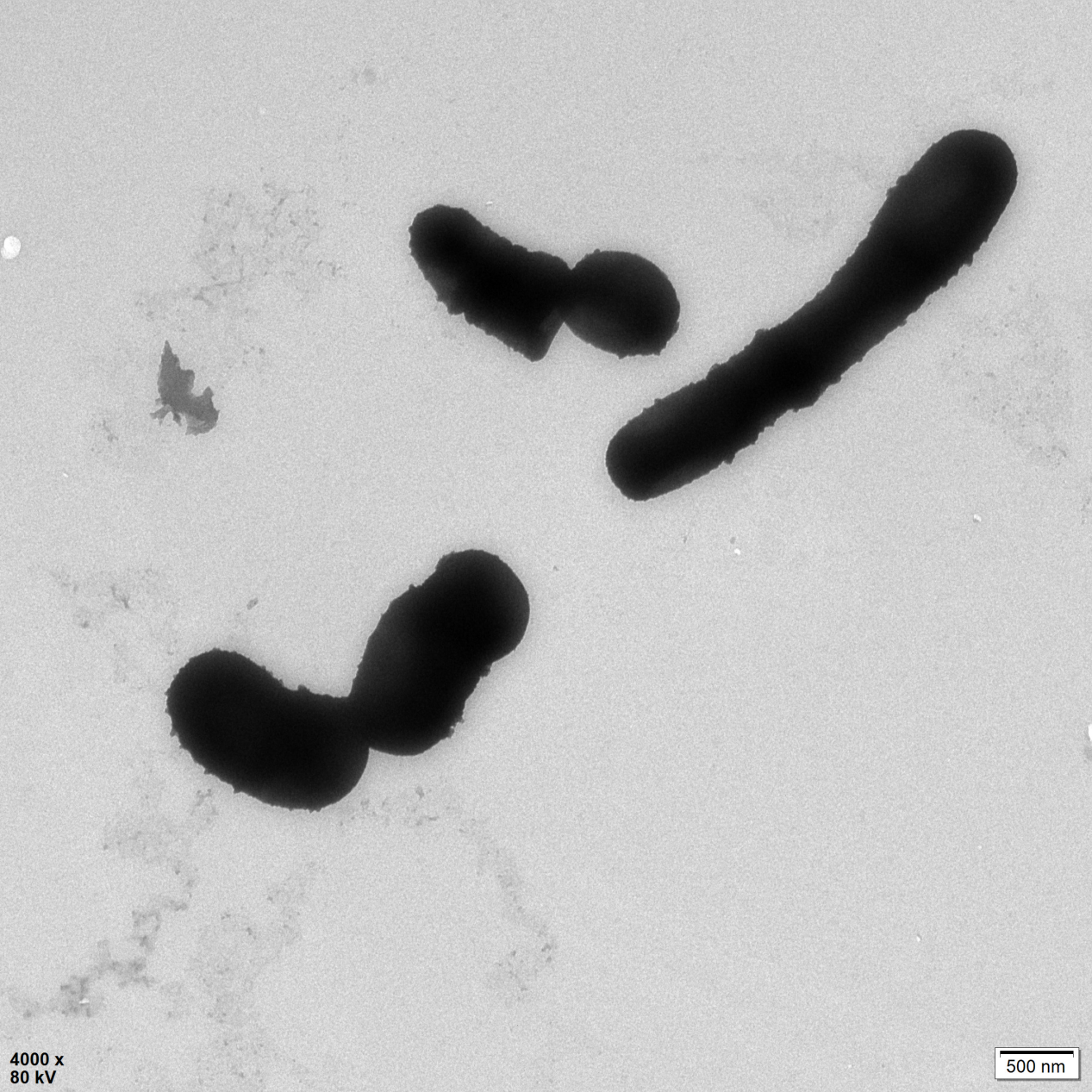


**Figure S3-C.** Transmission Electron micrograph of the Strain SCS-37 culture growth in MB at 30°C for 2 days depicts rod-shaped bacteria cells. Bar length, 500 nm.


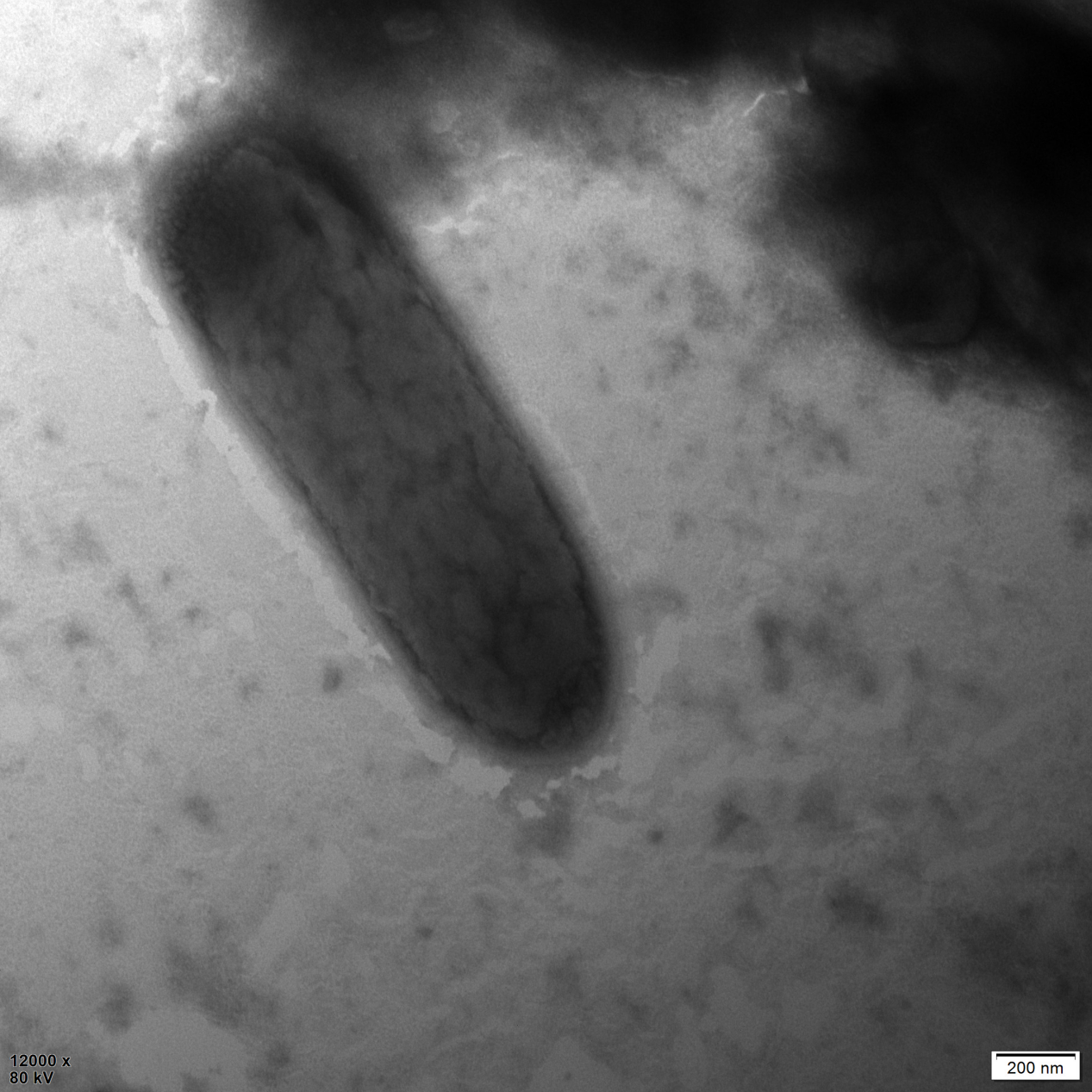


**Figure S3-D** Transmission Electron micrograph of the Strain SCS-31 culture growth in MB at 30°C for 2 days depicts rod-shaped bacteria cells. Bar length, 200 nm.


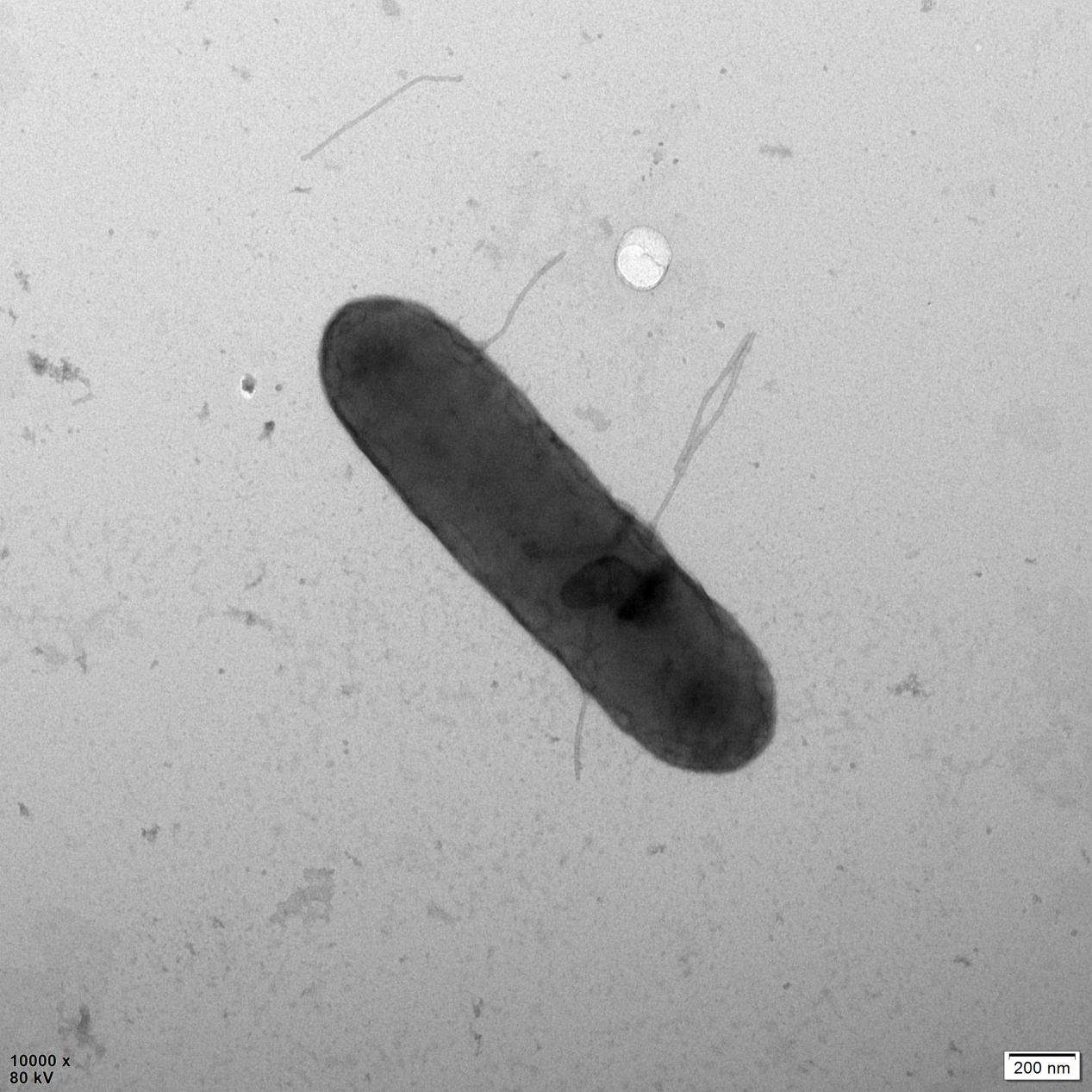


**Figure S3-E.** Transmission Electron micrograph of the Strain SCS-151 culture growth in MB at 30°C for 2 days depicts rod-shaped bacteria cells. Bar length, 200 nm.


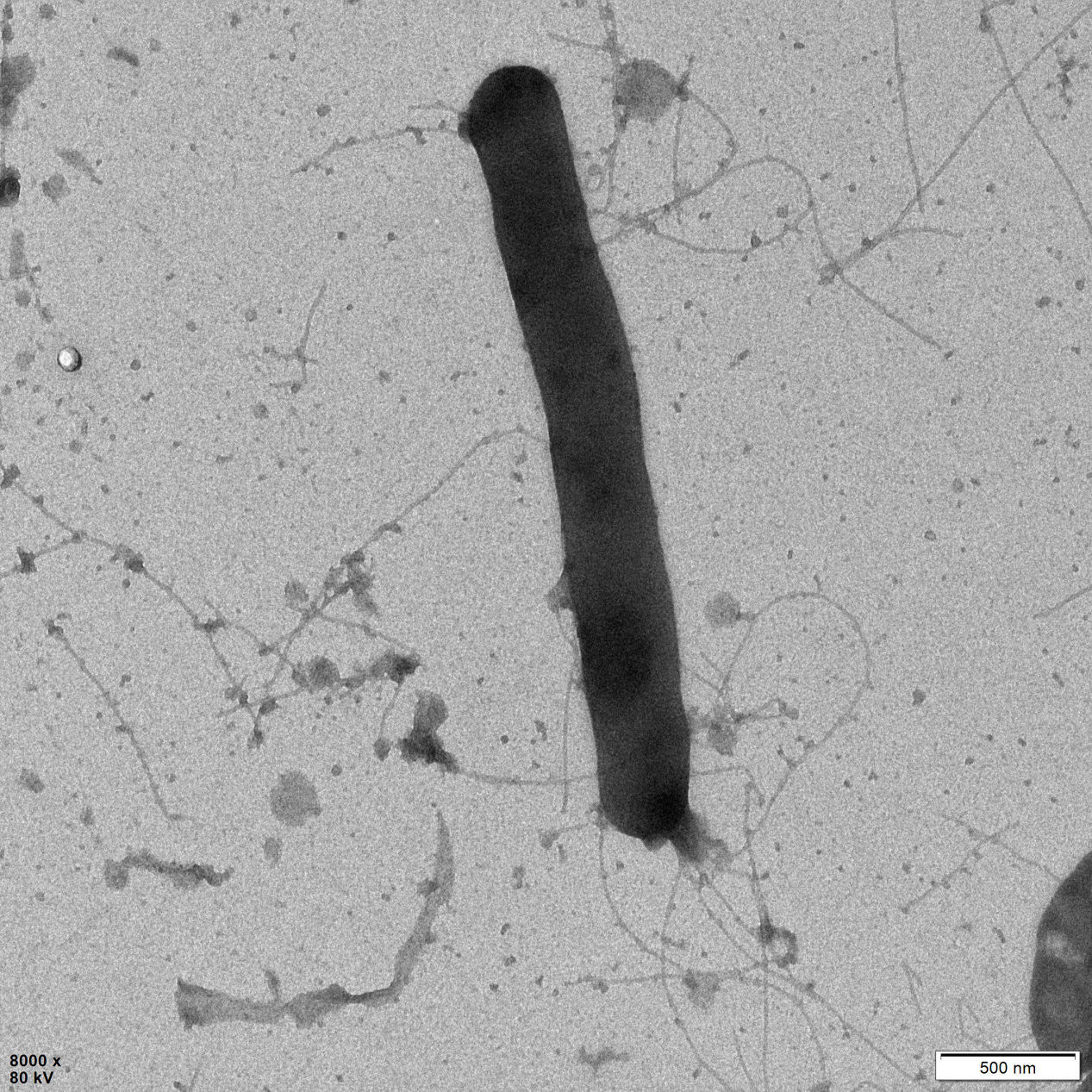


**Figure S3-F.** Transmission Electron micrograph of the Strain SCS-153A culture growth in MB at 30°C for 2 days depicts rod-shaped bacteria cells. Bar length, 500 nm.


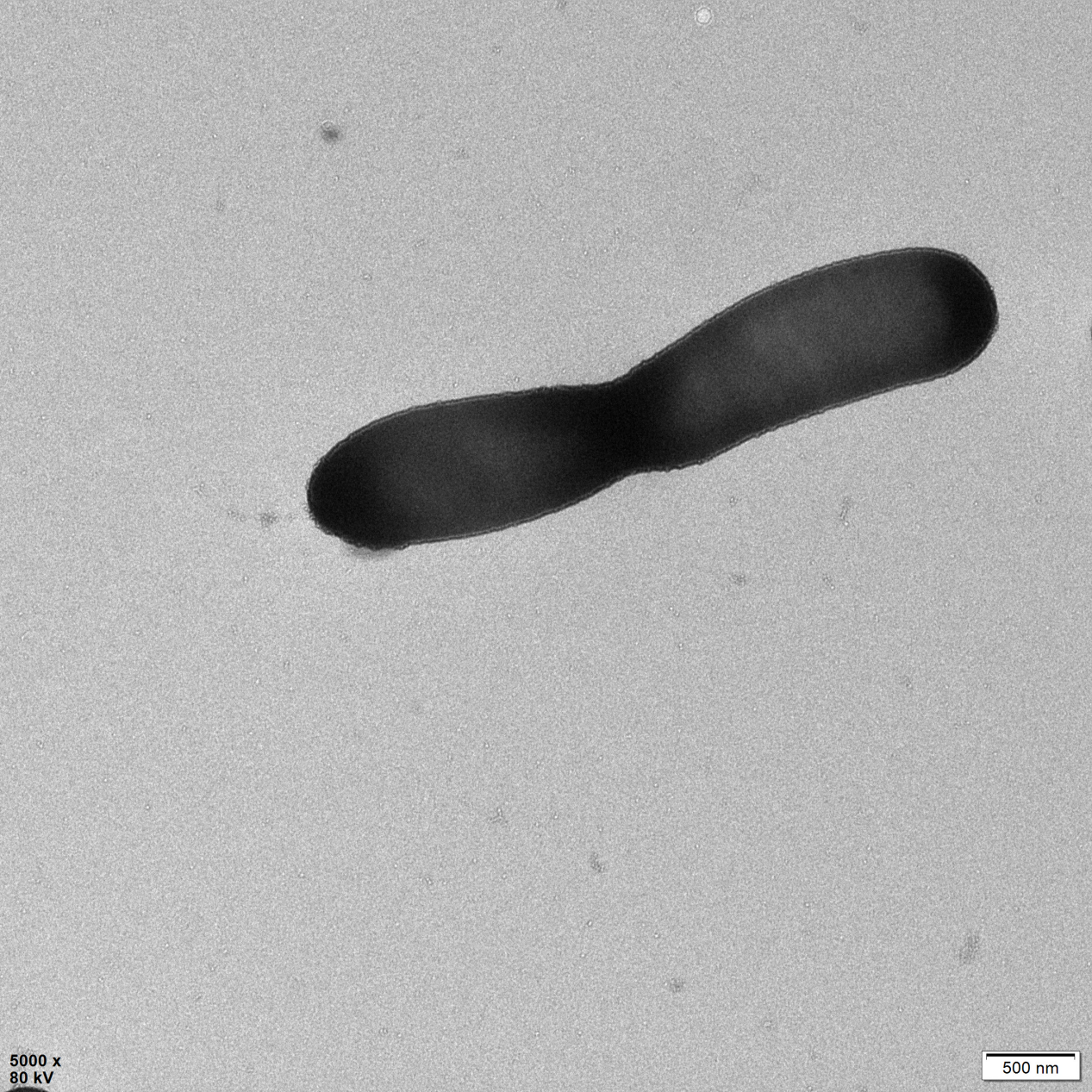


**Figure S6-C.** Transmission Electron micrograph of SCS-155 culture growth in MB at 30°C for 2 days depicts rod-shaped bacteria cells. Bar length, 500 nm.


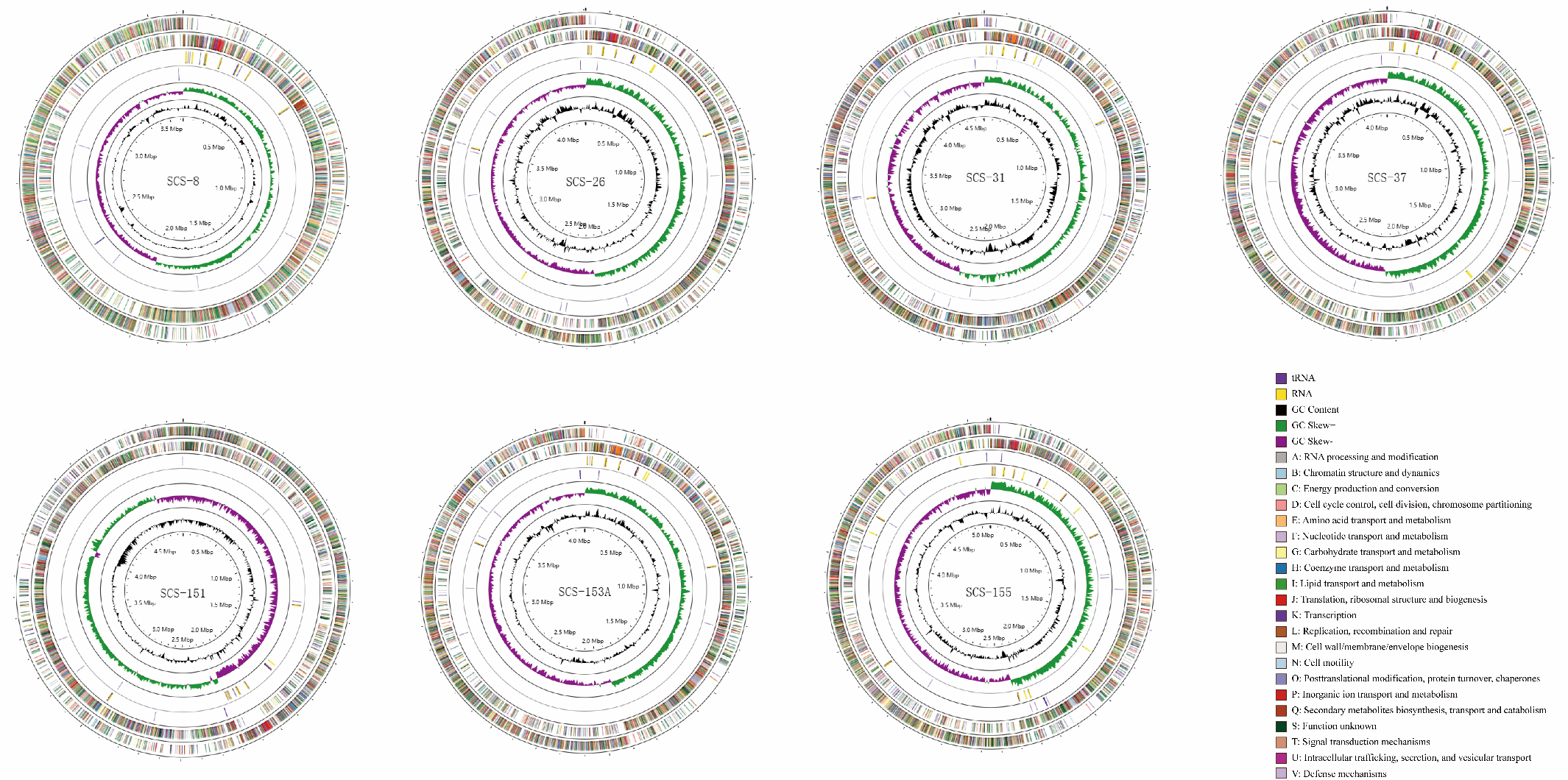


**Figure S7. Circular genome visualization of 7 strains.** From outside to the center: CDS on the forward and reverse strands, colored according to COG classification; non-coding RNAs on the forward and reverse strand; the G+C content; G+C skew and genome size

**
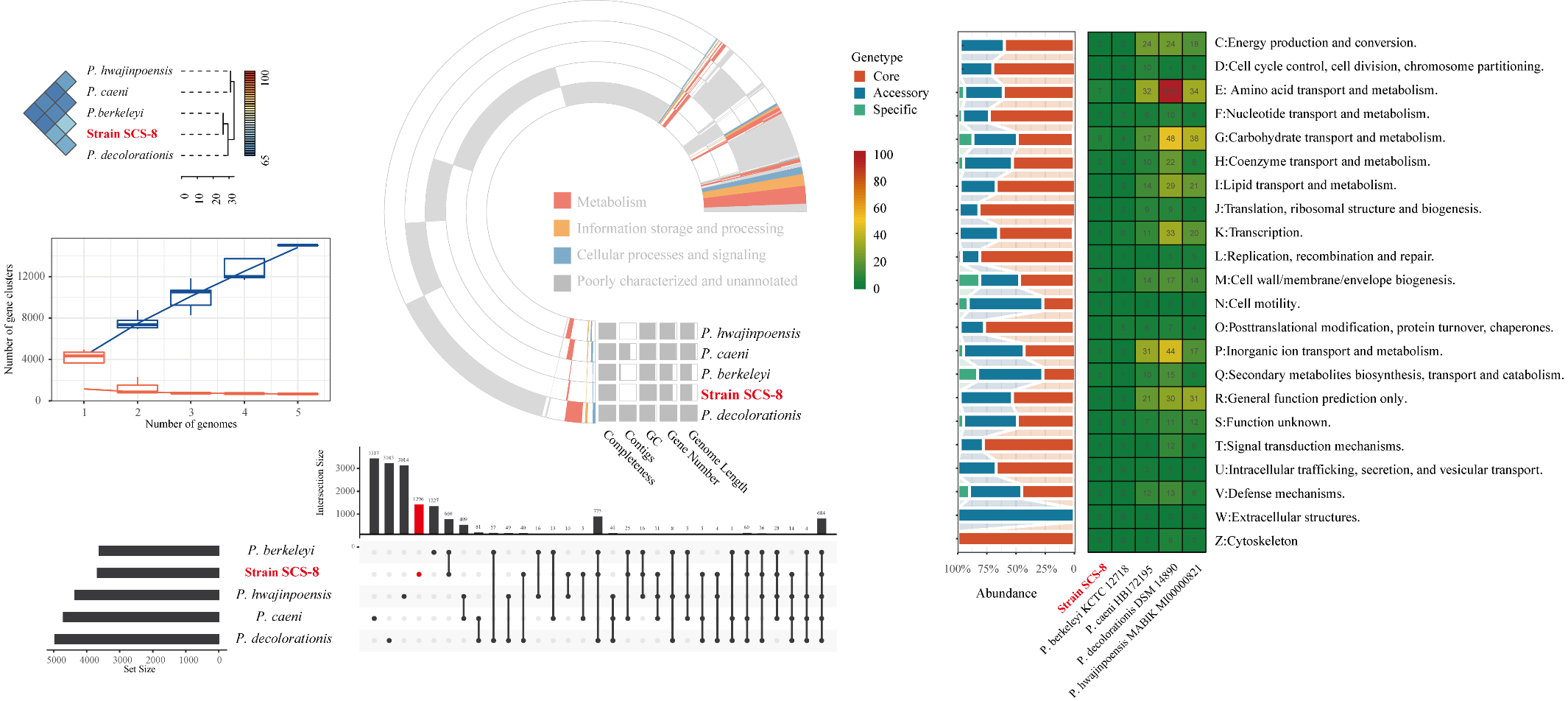
**

E

D

C

B

A

**Figure S8-A. Comparative genomic analysis of Strain SCS-8 and other 4 reference strains.** (A) Display the evolutionary relationships among 5 strains based on ANI values. (B) Display the core, accessory and specific gene sets annotated by COG categories. (C) Upset plot shows the intersection of gene sets across 5 strains. The bar graph at the top indicates the size of each intersection, while the dots and connecting lines below represent the specific species involved in each intersection. (D) Relative abundance of core, accessory and specific genes within Strain SCS-8 and heatmap of specific gene sets within 5 strains annotated by COG categories.

**
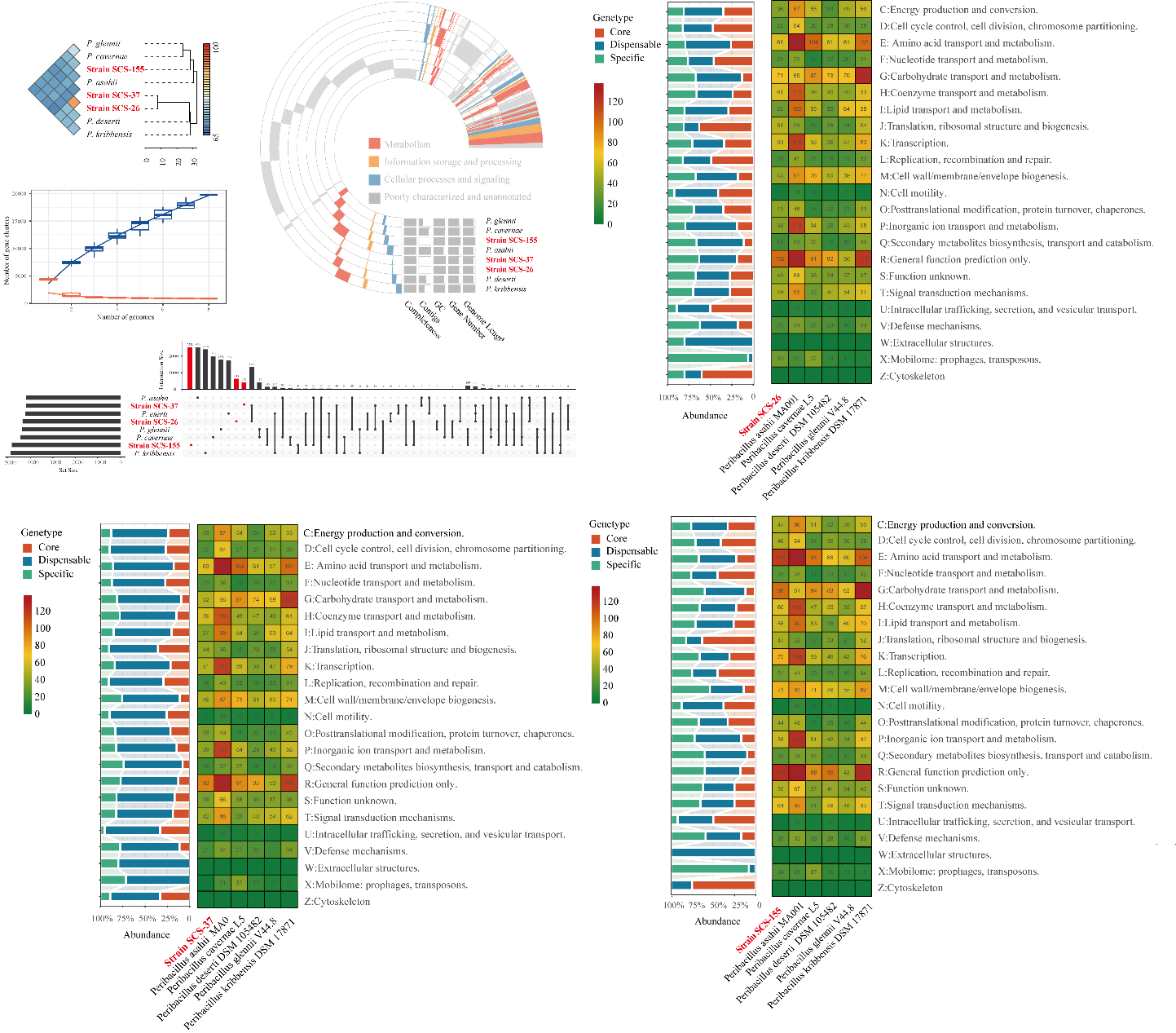
**

**G**

**F**

E

C

B

D

A

E

**Figure S8-B. Comparative genomic analysis of Strain SCS-26, SCS-37, SCS-155 and other 4 reference strains.** (A) Display the evolutionary relationships among 8 strains based on ANI values. (B) Display the core, accessory and specific gene sets annotated by COG categories. (C) Upset plot shows the intersection of gene sets across 8 strains. The bar graph at the top indicates the size of each intersection, while the dots and connecting lines below represent the specific species involved in each intersection. (D) Relative abundance of core, accessory and specific genes within Strain SCS-26, (E) SCS37, (F) SCS155 and heatmap of specific gene sets within other strains annotated by COG categories.

**
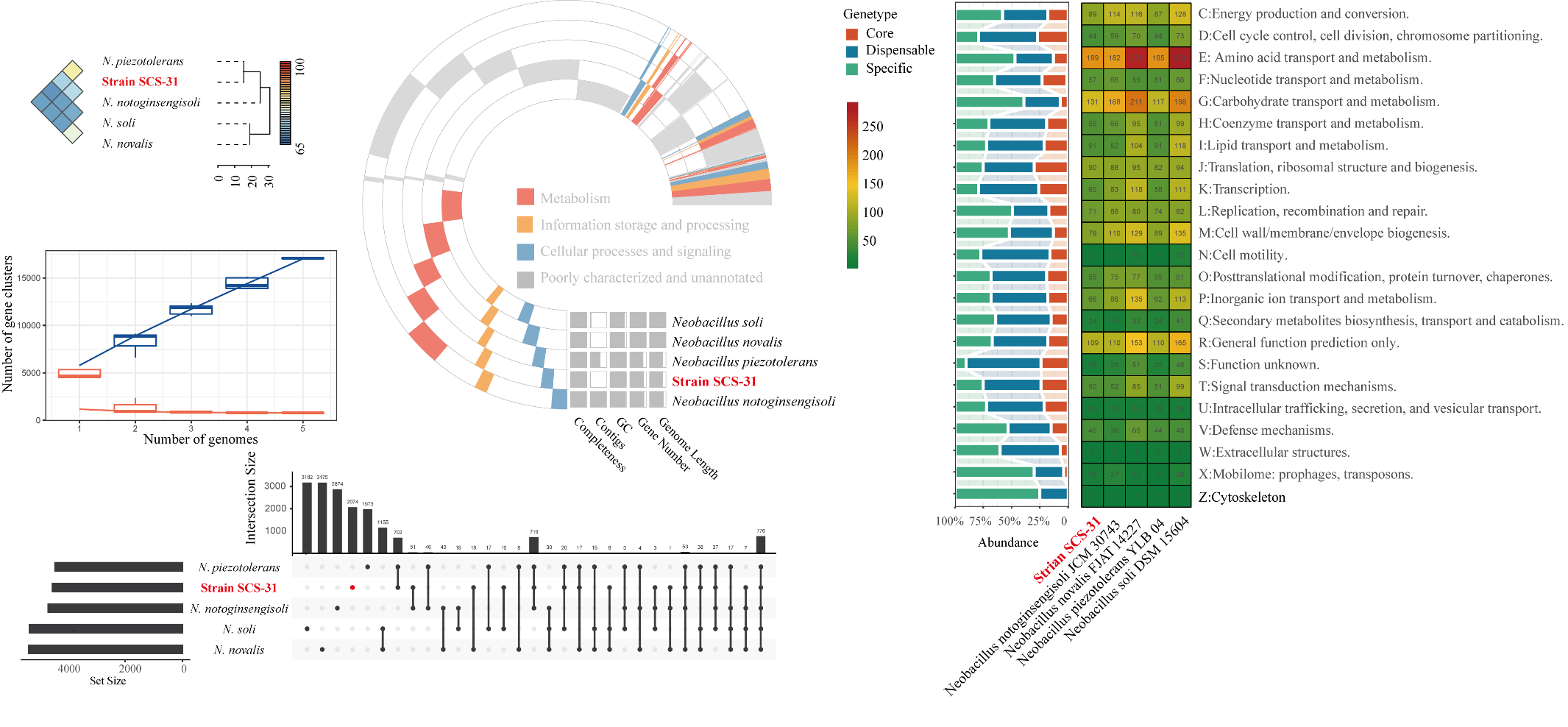
**

E

D

C

B

A

**Figure S8-A. Comparative genomic analysis of Strain SCS-31 and other 4 reference strains.** (A) Display the evolutionary relationships among 5 strains based on ANI values. (B) Display the core, accessory and specific gene sets annotated by COG categories. (C) Upset plot shows the intersection of gene sets across 5 strains. The bar graph at the top indicates the size of each intersection, while the dots and connecting lines below represent the specific species involved in each intersection. (D) Relative abundance of core, accessory and specific genes within Strain SCS-31 and heatmap of specific gene sets within 5 strains annotated by COG categories.

D

A

**
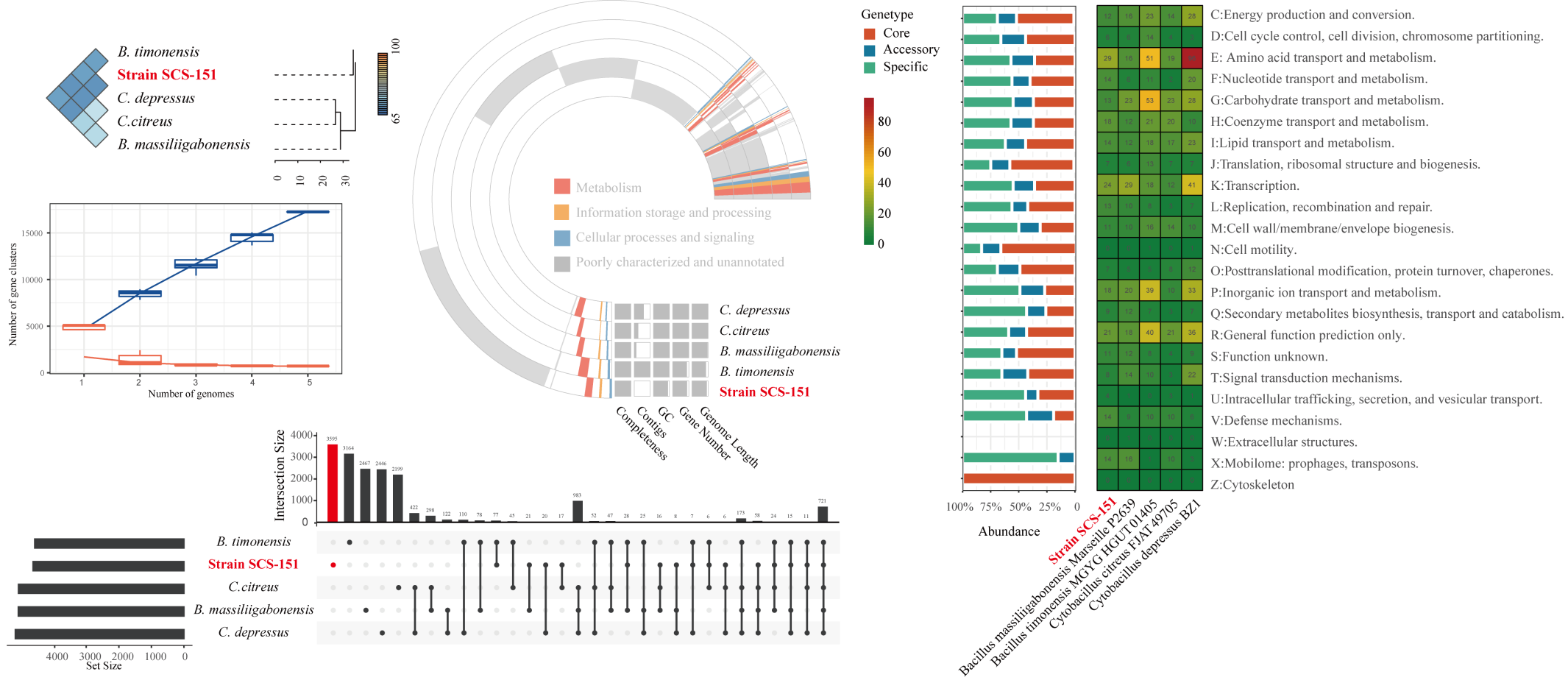
**

E

C

B

**Figure S8-A. Comparative genomic analysis of Strain SCS-151 and other 4 reference strains.** (A) Display the evolutionary relationships among 5 strains based on ANI values. (B) Display the core, accessory and specific gene sets annotated by COG categories. (C) Upset plot shows the intersection of gene sets across 5 strains. The bar graph at the top indicates the size of each intersection, while the dots and connecting lines below represent the specific species involved in each intersection. (D) Relative abundance of core, accessory and specific genes within Strain SCS-151 and heatmap of specific gene sets within 5 strains annotated by COG categories.

**
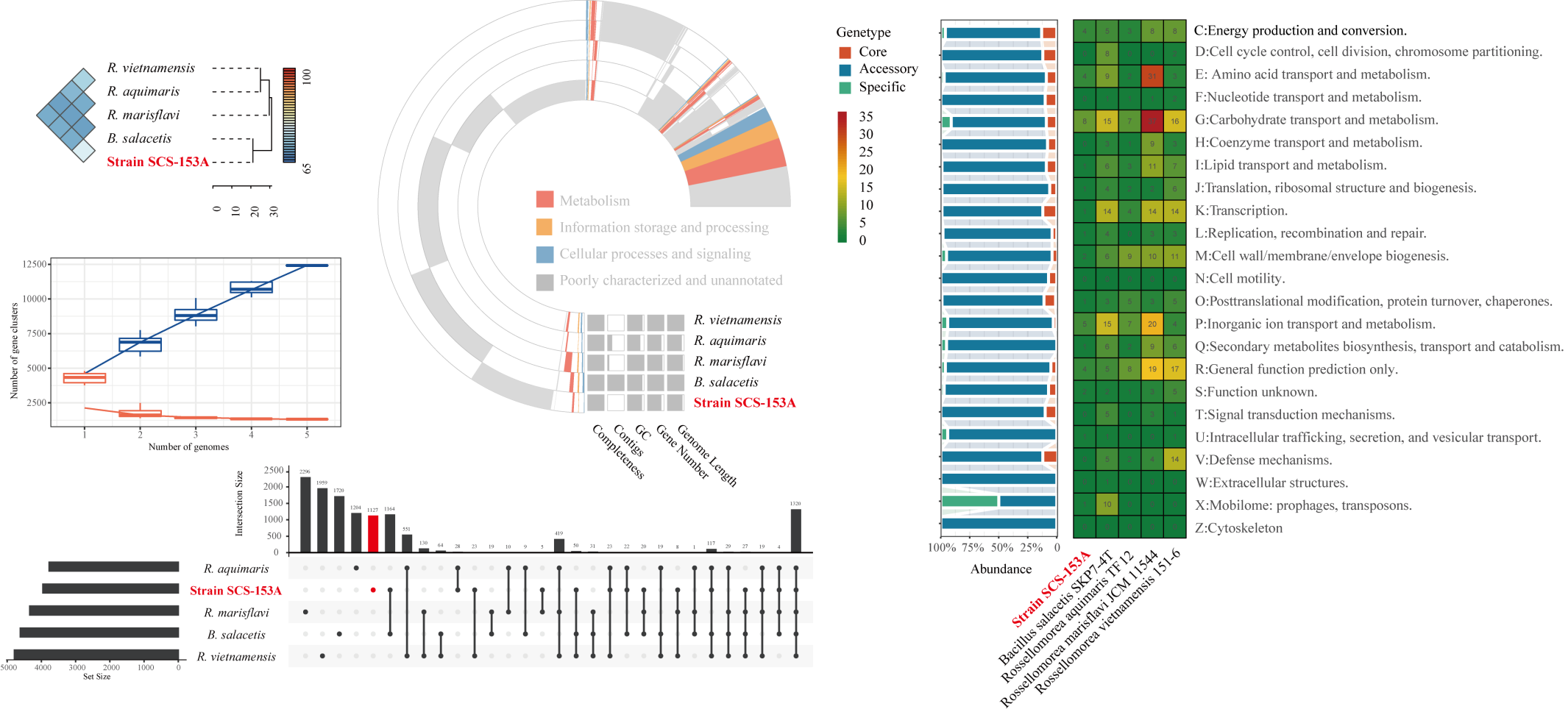
**

E

B

A

D

C

**Figure S8-A. Comparative genomic analysis of Strain SCS-153A and other 4 reference strains.** (A) Display the evolutionary relationships among 5 strains based on ANI values. (B) Display the core, accessory and specific gene sets annotated by COG categories. (C) Upset plot shows the intersection of gene sets across 5 strains. The bar graph at the top indicates the size of each intersection, while the dots and connecting lines below represent the specific species involved in each intersection. (D) Relative abundance of core, accessory and specific genes within Strain SCS-153A and heatmap of specific gene sets within 5 strains annotated by COG categories.


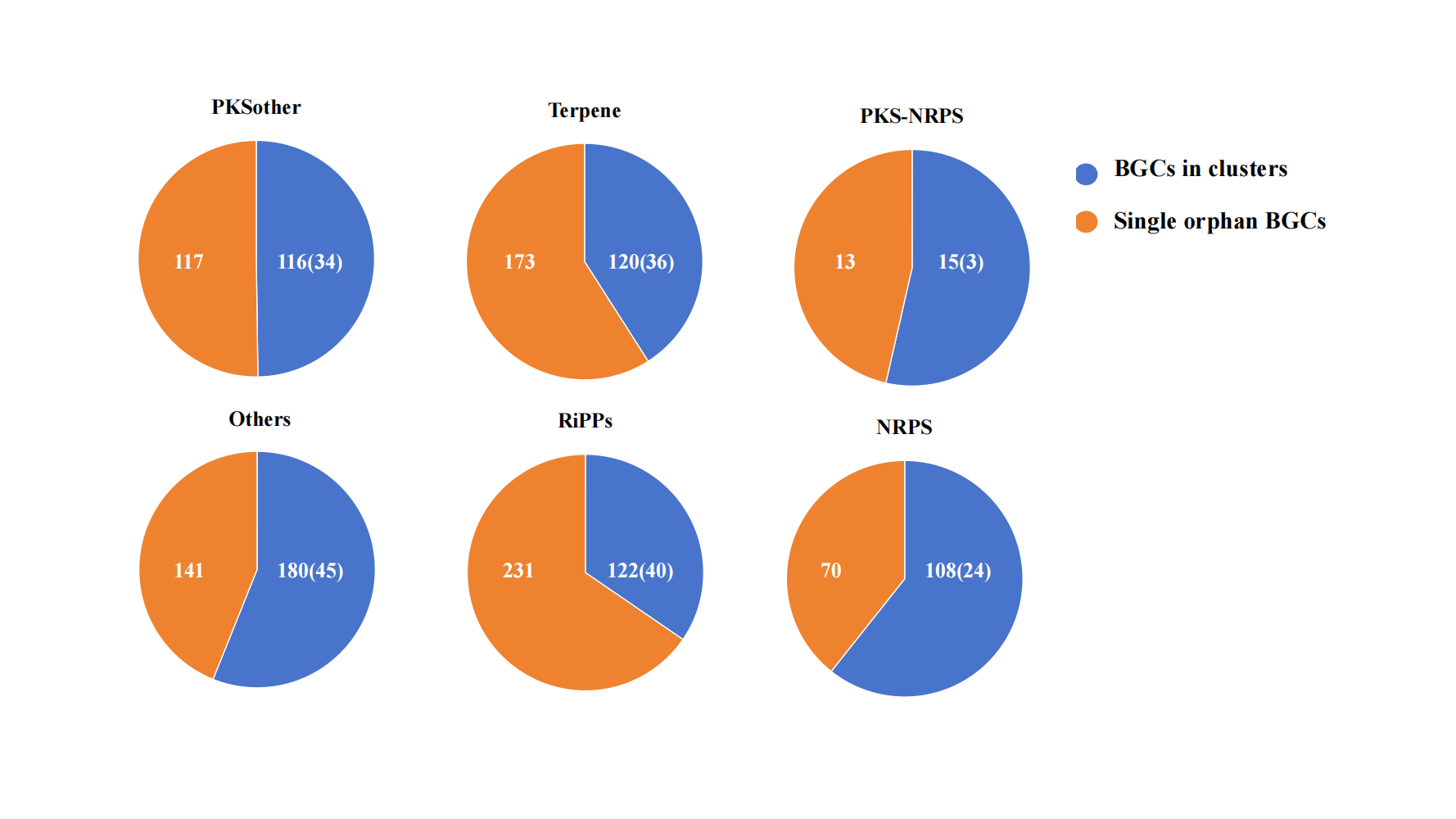


**Figure S9. The numbers of BGCs form clusters or single orphan one.**
